# Gram‐Scale Access to (3,11)‐Cyclotaxanes—Synthesis of 1‐Hydroxytaxuspine C

**DOI:** 10.1002/anie.202506245

**Published:** 2025-07-16

**Authors:** Philipp Schoch, Yulia Krivolapova, Fabian Schneider, Lu Pan, Tanja Gaich

**Affiliations:** ^1^ Department of Chemistry University of Konstanz Universitätsstrasse 10 78464 Konstanz Germany; ^2^ Research Center for (Bio‐)Synthesis of Active Small Molecules in Chinese Medicine Shanghai University of Traditional Chinese Medicine Shanghai 201203 China; ^3^ Longhua Hospital Shanghai University of Traditional Chinese Medicine 725 Wan‐Ping South Road Shanghai 200032 China

**Keywords:** Complex taxanes, Natural products, Photochemistry, Taxane diterpene, Total synthesis

## Abstract

Herein, we present the semisynthesis of complex taxane diterpenoid 1‐hydroxytaxuspine C. Starting from cheap and abundant 10‐deacetylbaccatin III, a scalable and robust route was developed, enabling an unprecedented gram‐scale access to the intricate (3,11)‐cyclotaxane scaffold. In addition, this represents the first synthetic access to C1‐hydroxylated cyclotaxanes. The natural product is synthesized in 17 steps, with the reactions being performed on decagram‐scale up to an advanced intermediate, establishing the scalability of this approach.

## Graphical abstract

Belonging to the family of complex taxanes, 1‐hydroxytaxuspine C is a densely functionalized natural product. The synthesis was realized via a semisynthetic approach, starting from commercially available 10‐deacetylbaccatin III. A late‐stage photocyclization was utilized to install the characteristic transannular bond of complex taxanes, transforming the classical framework into the intricate cyclotaxane skeleton on gram‐scale.

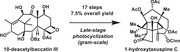

Taxanoid diterpenes or taxanes form a rich family of natural products, with more than 550 individual structures reported to date.^[^ ^1^ ^]^ They are produced as secondary metabolites in various slow growing evergreen shrubs of the genus Taxus (Taxus spp., Taxaceae). Many congeners exhibit significant bioactivity, in particular cytotoxicity, rendering them interesting candidates for pharmaceutical application.^[^ ^2^ ^]^ Among the numerous taxanes, Taxol (**3**) is the undisputed flagship in terms of its pharmaceutical exploration and considered to be one of the most important drugs used in cancer chemotherapy.^[^ ^3^, ^4^, ^5^ ^]^ Taxol (**3**) production was initially reliant on isolation of miniscule quantities from the bark of the Pacific yew, killing the tree in the process.^[^ ^6^ ^]^ Due to its scarcity and a limited, nonsustainable access via natural sources, the urgent need of alternative sourcing methods of Taxol (**3**) became obvious. Tremendous synthetic efforts resulted in 13 total and formal syntheses and countless synthetic studies that have been published to this date,^[^ ^7^ ^]^ advancing and enriching the field of organic chemistry along the way. Although academically appealing, none of these total syntheses could provide an economically viable access to the vast amounts of material needed sufficient for a pharmaceutical application. This challenge was overcome by applying the concept of semisynthesis, relying on 10‐deacetylbaccatin III (10‐DAB, **2**) as starting material.^[^ ^8^, ^9^ ^]^ Structurally closely related to Taxol (**3**), the high abundance of 10‐DAB (**2**) in the twigs and needles of the European yew and its concomitant accessibility via nondestructive isolation rendered it the ideal starting material for a sufficient large‐scale production of Taxol (**3**). For over 20 years, semisynthetic processes starting from 10‐DAB (**2**) supplied most of the global demand of Taxol (**3**). Only recently this approach was surpassed by plant cell fermentation techniques.^[^ ^10^, ^11^ ^]^

From a chemical point of view, the scientific interest in taxanoid diterpenes arises not only from the diverse pharmaceutical activity of many congeners but also from their remarkably high structural complexity due to the densely functionalized framework. Most of these compounds, including Taxol (**3**), feature a 6/8/6 tricyclic core structure and are commonly referred to as classical taxanes.^[^ ^12^ ^]^ Based on the classical 6/8/6‐motif, modifications of this carbon backbone lead to the so‐called nonclassical taxanes.^[^ ^12^ ^]^ With 11 unique scaffolds, the structural diversity of taxane natural products therefore extends far beyond the classical framework.^[^ ^1^, ^12^ ^]^ However, the landscape of nonclassical taxanes remains largely unexplored in terms of their biological, pharmaceutical, and chemical properties, partially owed to the scarcity, structural complexity, and a lack of synthetic accessibility (Scheme 1).

**Scheme 1:**
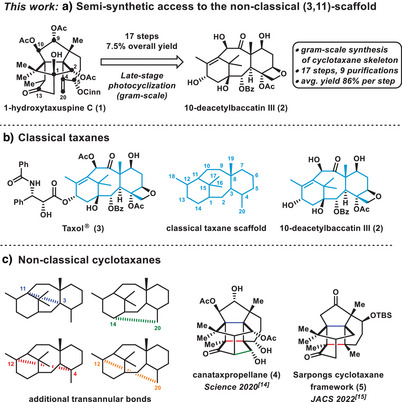
a) Semisynthetic access to (3,11)‐cyclotaxanes applied in the transformation from 10‐DAB (**2**) to 1‐hydroxytaxuspine C (**1**) (this work). b) Classical taxanes. c) Nonclassical cyclotaxane natural products.

Nonclassical taxane scaffolds constructed via formation of up to three out of four possible transannular bonds (C3–C11, C14–C20, C12–C4, C12–C20) embedded into the classical 6/8/6 core structure are classified as complex or cyclo‐taxanes. These compounds represent the most structurally complex subfamily of nonclassical taxanes, rendering them intriguing targets for the synthetic chemist. In 2020, we reported the total synthesis of canataxpropellane (**4**), representing the first ever synthesis of a cyclotaxane natural product.^[^ ^13^ ^]^ Sarpong and coworkers published their synthesis of a nonclassical cyclotaxane framework **5** in 2022.^[^ ^14^ ^]^ Recently, our report on a generalized strategy for the total synthesis of taxane diterpenes was published, unlocking access to an unprecedented amount of cyclotaxane scaffolds with vast structural diversity.^[^ ^15^ ^]^

Amongst all cyclotaxanes, with the exception of one scaffold,^[^ ^16^ ^]^ the C3–C11 transannular bond represents a common structural feature. Taxane congeners exhibiting exclusively this transannular C–C bond are termed (3,11)‐cyclotaxanes and represent the biggest subclass of cyclotaxanes, with 32 natural products isolated to date.^[^ ^1^, ^17^, ^18^, ^19^, ^20^ ^]^ Preliminary studies identified noncytotoxic congeners exhibiting biological activity as P‐glycoprotein (Pgp) modulators, rendering them interesting potential multidrug resistance (MDR) reversal agents.^[^ ^21^, ^22^, ^23^ ^]^ By establishing a synthetic access to these structures, we hope to enable first structure–activity relationship (SAR) studies, aiming for a better understanding of the functionalization pattern and its consequences for biological activity. For instance, it is known that the activity of Taxol (**3**) is enhanced by the presence of a C1‐hydroxyl group compared to the deoxygenated analogue.^[^ ^24^ ^]^ However, no synthesis of C1‐functionalized cyclotaxanes has been published to date. It became obvious that by extending our synthetic portfolio from C1‐unfunctionalized cyclotaxanes^[^ ^12^, ^13^, ^15^ ^]^ with their functionalized congeners, we would cover a broader range of cyclotaxanes, enabling more comprehensive SAR investigations. These studies are inherently tied to a potential medicinal application, however for this purpose, concerns about scalability and economic viability of accessing these intricate structures via total synthesis arise. Inspired by the implementation of semisynthesis in the large‐scale production of Taxol (**3**), we envisioned that a similar approach might be feasible toward complex taxanes. With natural product 1‐hydroxytaxuspine C (**1**) representing the most prominent (3,11)‐cyclotaxane scaffold and being hydroxylated at the C1‐position, we selected **1** as the ideal target to investigate such a semisynthetic approach. It was first isolated from the Japanese yew (*Taxus cuspidata*) in 1999 by Shi et al.^[^ ^25^ ^]^ and without recognizing the former report at the time by Kosugi et al. in 2000 (named therein as 3,11‐cyclotaxinine NN‐2).^[^ ^22^ ^]^ 1‐Hydroxytaxuspine C (**1**) exhibits remarkable bioactivity as MDR modulator, causing significantly increased cellular accumulation of vincristine in multidrug‐resistant human ovarian cancer 2780AD cells,^[^ ^22^ ^]^ with no concurrent signs of cytotoxicity.^[^ ^26^ ^]^ The structure features a C1‐hydroxylated (3,11)‐cyclotaxane scaffold with a C4(20)‐exomethylene moiety, a C13‐oxo group, three acetate groups at C2, C9, and C10, and a cinnamoyl side chain at C5.

In line with our goal of establishing a semisynthetic access to cyclotaxanes and in contrast to classical retrosynthetic analysis, we based our synthetic planning mainly on potential starting materials. With broad and reliable availability due to its pivotal role in the large‐scale production of Taxol (**3**), 10‐deacetylbaccatin III (**2**) presented the ideal starting point for such an endeavor. After screening the copious amount of literature on classical taxane reactivity and carefully orchestrating the necessary functional group interconversions, we developed a strategy to convert classical taxane 10‐DAB (**2**) into (3,11)‐cyclotaxane **1** with a minimal amount of protecting groups needed along the way.

Concerning the installation of the required C3–C11 bond, different strategies could be followed. Biosynthetically, this transannular bond is postulated to originate from a transannular Michael addition between an oxidized allylic system at C3 and an enone as Michael acceptor at C11.^[^ ^1^, ^12^, ^27^ ^]^ However, following a biomimetic approach did not seem feasible to us due to the required functionalization at C3. Instead, we opted to attempt the C3–C11 transannular bond formation under photochemical conditions from enone **19**, inspired by literature‐known photocyclizations of similar structures. Chiang et al. first discovered a photochemical transannular cyclization of a classical taxane in 1967.^[^ ^28^ ^]^ Subsequent reports by Kobayashi et al.^[^ ^23^ ^]^ and Sako et al.^[^ ^29^ ^]^ described a similar reactivity of their substrates. The (3,11)‐photocyclization of Taxol (**3**) itself has been reported by Chen et al.,^[^ ^30^ ^]^ albeit presumably occurring via a different reaction mechanism than the former examples. Cyclization precursor **19** should be obtainable through simple functional group interconversions (FGIs), including cleavage of the oxetane moiety, from inexpensive 10‐DAB (**2**).

Commencing from commercially available 10‐deacetylbaccatin III (**2**), the synthesis of 1‐hydroxytaxuspine C (**1**) proceeded as displayed in Scheme 2. Acetylation following a known procedure^[^ ^31^ ^]^ gave baccatin III (**6**). C7‐Defunctionalization was accomplished via a modified elimination–hydrogenation sequence,^[^ ^32^ ^]^ delivering literature‐known^[^ ^33^ ^]^ 7‐deoxy‐baccatin III (**9**). Notably, this provides a significantly improved access to 7‐deoxytaxanes compared to earlier procedures based on radical deoxygenation, often suffering from radical rearrangements.^[^ ^33^, ^34^ ^]^ Protection of the allylic alcohol gave silyl ether **10** in a remarkable yield of 61% over a 5‐step sequence with a single purification. Compound **10** was treated with Red‐Al^[^ ^35^ ^]^ to remove the C2‐benzoate yielding diol **11**, which was subsequently protected as a C1–C2 carbonate with triphosgene.^[^ ^36^ ^]^ Up to this point, all steps were conducted on decagram‐scale. We then turned our attention toward transformation of the oxetane moiety into the desired exomethylene motif. Screening the literature on d‐secotaxanes, an oxetane ring cleavage under Lewis acidic conditions emerged as the most promising strategy.^[^ ^37^, ^38^, ^39^, ^40^ ^]^ After careful optimization, opening of the oxetane d‐ring was realized with TiCl_4_.^[^ ^39^ ^]^ In line with literature,^[^ ^37^, ^39^, ^40^, ^41^, ^42^ ^]^ this transformation was found to be highly temperature sensitive (cf. Supporting Information). Conversion of the newly formed diol **13** to a thiocarbonate with TCDI and subsequent Corey–Winter elimination^[^ ^39^, ^43^, ^44^ ^]^ delivered **14** containing the desired exomethylene moiety. Deacetylation of the C10‐acetate with K_2_CO_3_ in MeOH provided α‐hydroxy ketone **15**, which was diastereoselectively reduced to *trans*‐diol **16** with sodium amalgam by adapting a procedure developed by the Baran group, following their seminal report on C9 reduction of taxanoid structures to the corresponding C9–C10 *trans*‐diol derivatives.^[^ ^45^ ^]^ Subsequent bisacetylation delivered triacetate **17**. The temporary deacetylation at C10 was necessary to prevent an otherwise occurring deacetoxylation when attempting the direct reduction of compound **14** with sodium amalgam, leading to the respective C10‐methylene derivative. Desilylation of **17** with TBAF and subsequent allylic oxidation with MnO_2_ gave enone **18,** with all steps up to this point being carried out on gram‐scale. To install the desired C2‐acetate, acetylation of a liberated C2‐hydroxyl group was envisioned. However, attempted carbonate cleavage under hydrolytic or reductive conditions failed to provide C2‐OH and led to complex mixtures. As opening of a C1–C2 carbonate on taxanoid structures with organolithium reagents to the corresponding C2‐esterification product is well precedented in literature,^[^ ^36^, ^46^ ^]^ a nucleophilic approach employing methyllithium was performed. This successfully led to cleavage of the carbonate, accompanied by partial deacetylation of the various C2/C5/C9/C10‐acetates and the obtained mixture was directly submitted to reacetylation conditions. As the C5‐hydroxyl group proved to be rather unreactive, allylic alcohol **19** could be obtained, combining carbonate cleavage toward the C2‐acetate and simultaneous C5‐deacetylation in a single step. With the correct acetylation pattern in place, compound **19** was submitted to photocyclization conditions. Irradiation at 254 nm under thorough exclusion of oxygen cleanly provided (3,11)‐cyclotaxane scaffold **20** with 1.04 g of product being obtained in a single batch, demonstrating the scalability of this approach.

**Scheme 2:**
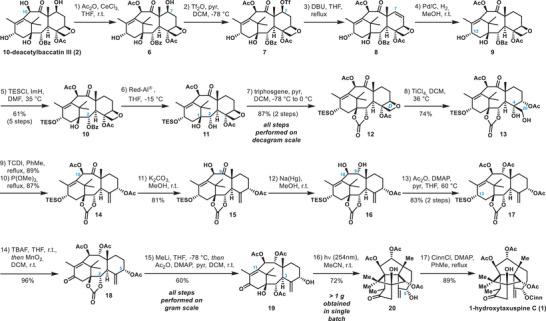
Semisynthesis of 1‐hydroxytaxuspine C (**1**). THF, tetrahydrofuran; pyr, pyridine; DCM, dichloromethane; DBU, 1,8‐diazabicyclo[5.4.0]undec‐7‐ene; TESCl, chlorotriethylsilane; ImH, imidazole; DMF, *N,N*‐dimethylformamide; Red‐Al, sodium bis(2‐methoxyethoxy)aluminum hydride; TCDI, thiocarbonyl diimidazole; DMAP, 4‐dimethylaminopyridine; TBAF, tetrabutylammonium fluoride; CinnCl, *trans*‐cinnamoyl chloride.

Notably, no other reactivity besides the desired C3–C11 transannular bond formation was observed, with compound **20** being the only obtained product. The exact mechanism of this transformation has not been elucidated yet. Investigations by Kobayashi et al. on similar substrates have detected the involvement of triplet states.^[^ ^47^ ^]^ One possible reaction mechanism^[^ ^12^, ^27^, ^47^ ^]^ for the cyclization is presented in Scheme 3 and might proceed as follows: excitation of the enone to the first excited singlet state followed by intersystem crossing to the first excited triplet state would provide intermediate **21**, exhibiting biradical character. Facilitated by the close spatial proximity between C3 and C12,^[^ ^28^, ^47^ ^]^ triplet intermediate **21** might undergo a 1,6‐HAT (hydrogen atom transfer), resulting in a tertiary, allylic radical in intermediate **22**. Recombination of this C3,C11‐diradical intermediate might then form the transannular C3–C11 bond. A reaction pathway via a concerted _σ_2_s_ + _π_2_s_ route has been proposed as well.^[^ ^47^ ^]^ Finally, acylation at C5 with cinnamoyl chloride delivered the corresponding natural product 1‐hydroxytaxuspine C (**1**) in an overall yield of 7.5% over 17 steps.

**Scheme 3:**
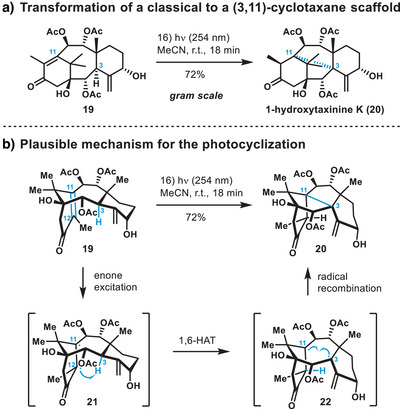
a) Scalable (3,11)‐cyclotaxane synthesis via late‐stage photochemical transannular bond formation. Over one gram of cyclotaxane **20** was synthesized in a single batch. b) Plausible mechanism for the photocyclization. HAT, hydrogen atom transfer.

In conclusion, we have developed a semisynthetic access to 1‐hydroxytaxuspine C (**1**) in 17 steps with an overall yield of 7.5%, starting from commercially available 10‐DAB (**2**). Due to the abundant starting material and extensive optimization by consciously orchestrating the required transformations, an unprecedented gram‐scale access to the (3,11)‐cyclotaxane framework in 16 steps is provided. Our findings therefore not only enable further chemical and pharmaceutical investigation of these intriguing molecules but also represent the first synthetic access into C1‐hydroxylated cyclotaxane scaffolds, thereby expanding the chemical space available for structure–activity relationship studies significantly.

## Supporting Information

The authors have cited additional references within the Supporting Information.^[^ ^48^, ^49^ ^]^

## Conflict of Interests

The authors declare no conflict of interest.

## Supporting information



Supporting Information

## Data Availability

The data that support the findings of this study are available in the Supporting Information of this article.
