# Supplementary material for: Gram‐Scale Access to (3,11)‐Cyclotaxanes—Synthesis of 1‐Hydroxytaxuspine C
Source: Angew Chem Int Ed Engl. 2025 Jul 16;64(35):e202506245. doi: 10.1002/anie.202506245 (PMC12377431; doi:10.1002/anie.202506245)

Gram-scale Access to (3,11)-Cyclotaxanes – Synthesis of 1-Hydroxytaxuspine C

Supporting Information

Philipp Schoch[a], Yulia Krivolapova[a], Fabian Schneider[b], Lu Pan[a]* and Tanja Gaich[a]*

[a] *Department of Chemistry, University of Konstanz*

Universitätsstrasse 10, 78464 Konstanz (Germany)

[b] *Research Center for (Bio-)Synthesis of Active Small Molecules in Chinese Medicine, Shanghai University of Traditional Chinese Medicine, Shanghai 201203, China;*

Longhua Hospital, Shanghai University of Traditional Chinese Medicine, 725 Wan-Ping South Road, 200032, Shanghai, China

Table of Contents

1. General Methods 1

2. Experimental procedures 2

3. Comparison of Natural and Synthetic 1-Hydroxytaxuspine C NMR Data 25

4. References 28

5. NMR Spectra 29

1. General Methods

Reactions sensitive to oxygen or moisture were carried out in flame-dried glassware under a slight nitrogen overpressure using standard Schlenk techniques. Sensitive solutions, solvents or reagents were transferred *via* cannula or syringe. All reactions were stirred magnetically. Solvent evaporations were conducted under reduced pressure at temperatures less than 40 °C, unless otherwise noted. Further drying of the residues was accomplished using a high vacuum pump. Dry solvents were purchased from Acros-Organics and used as received. Ethyl acetate and hexane for extraction and column chromatography were purchased in technical grade and distilled before usage. Commercially available reagents were used as supplied unless stated otherwise. 10-Deacetylbaccatin III was purchased from Suzhou Ryway Biotech Co., Ltd and used as received. Reactions were monitored by thin-layer chromatography (TLC) with silica gel 60-F254 aluminium plates from Merck. Detection of the spots was performed by using a UV lamp (254 nm) and subsequent visualization employing a cerium ammonium molybdate-based TLC stain. Column chromatography was performed on silica gel 60 M (0.040 - 0.063 mm) from Macherey-Nagel. NMR spectra were measured on a JEOL Lambda 400 spectrometer. Chemical shifts are given in ppm and referenced to residual solvent peaks. Data are reported as follows: chemical shift, multiplicity (s = singlet, bs = broad singlet, d = doublet, t = triplet, q = quartet, m = multiplet), coupling constant *J*, integration. Optical rotation was measured on a JASCO P-2000 digital polarimeter using the sodium D line (589 nm). Infrared spectra were recorded as thin films of pure product with a Smart iTX Diamond ATR unit on a ThermoFisher Scientific Nicolet iS20 spectrometer. High-resolution mass spectra (HRMS) were measured on a Fischer Scientific Orbitrap Velos Pro. Ionization was achieved by ESI, modes of ionization, calculated and found mass are given.

2. Experimental procedures

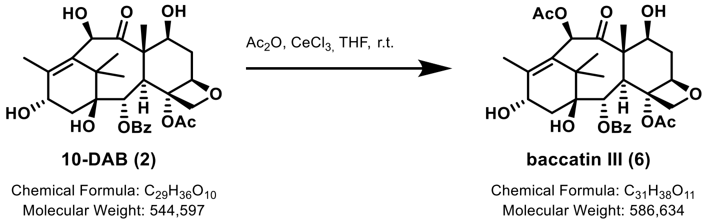

(2a*R*,4*S*,4a*S*,6*R*,9*S*,11*S*,12*S*,12a*R*,12b*S*)-12-(benzoyloxy)-4,9,11-trihydroxy-4a,8,13,13-tetramethyl-5-oxo-3,4,4a,5,6,9,10,11,12,12a-decahydro-1*H*-7,11-methanocyclodeca[3,4]benzo[1,2-*b*]oxete-6,12b(2a*H*)-diyl diacetate (baccatin III) (6)

10-Deacetylbaccatin III (**2**) (20.0 g, 36.7 mmol, 1.00 eq.) and anhydrous cerium trichloride (905 mg, 3.67 mmol, 0.10 eq.) were dissolved in dry THF (695 mL) under nitrogen atmosphere. Acetic anhydride (32.3 mL, 367 mmol, 10.0 eq.) was added and the reaction mixture was stirred for 4 h at room temperature. The reaction was quenched with aqueous $NaHCO_3$ and the phases were separated. The aqueous phase was extracted with ethyl acetate, the combined organic phases were washed with brine and dried over $MgSO_4$. The solvents were evaporated under reduced pressure, yielding 23.4 g of crude baccatin III (**6**) as a white powder, which was used in the next step without further purification and quantitative yield was assumed.

An analytically pure sample was obtained by column chromatography on silica (EtOAc/hexane, 4:1 v/v).

R_f = 0.29 (EtOAc/hexane = 3/1 (*v/v*), CAM).

$[\alpha]^D_{20}$: –104.47° (c=0.71, $CHCl_3$).

^{1}H NMR (400 MHz, $CDCl_3$): δ 8.13 – 8.06 (m, 2H), 7.65 – 7.56 (m, 1H), 7.52 – 7.44 (m, 2H), 6.32 (s, 1H), 5.62 (d, *J* = 7.0 Hz, 1H), 4.98 (dd, *J* = 9.7, 2.2 Hz, 1H), 4.90 – 4.86 (m, 1H), 4.51 – 4.41 (m, 1H), 4.30 (d, *J* = 7.9 Hz, 1H), 4.15 (d, *J* = 8.5 Hz, 1H), 3.88 (d, *J* = 7.1 Hz, 1H), 2.62 – 2.48 (m, 1H), 2.51 (d, *J* = 4.2 Hz, 1H), 2.32 – 2.21 (m, 2H), 2.28 (s, 3H), 2.24 (s, 3H), 2.22 – 2.14 (m, 1H), 2.05 (d, *J* = 1.5 Hz, 3H), 1.86 (ddd, *J* = 14.7, 10.9, 2.3 Hz, 1H), 1.67 (s, 3H), 1.65 (s, 1H), 1.11 (s, 6H).

^{13}C NMR (101 MHz, $CDCl_3$): δ 204.3, 171.5, 170.8, 167.2, 146.5, 133.8, 132.0, 130.2 (2C), 129.5, 128.8 (2C), 84.6, 80.9, 79.2, 76.6, 76.4, 75.1, 72.5, 68.1, 58.9, 46.3, 42.9, 38.8, 35.7, 27.1, 22.7, 21.0 (2C), 15.7, 9.6.

IR (film, cm^{-1}): 2925, 1710, 1452, 1371, 1313, 1239, 1176, 1070, 1022.

HRMS (ESI): *m/z* calcd for $C_{31}H_{38}O_{11}Na^+$ $[M+Na]^+$: 609.2306, found: 609.2300.

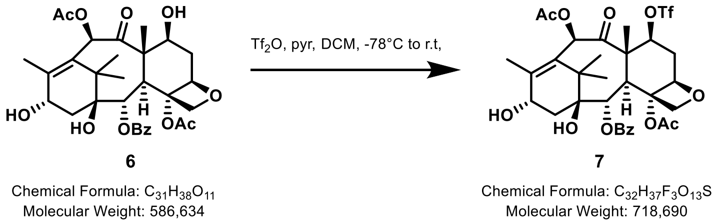

(2a*R*,4*S*,4a*S*,6*R*,9*S*,11*S*,12*S*,12a*R*,12b*S*)-12-(benzoyloxy)-9,11-dihydroxy-4a,8,13,13-tetramethyl-5-oxo-4-(((trifluoromethyl)sulfonyl)oxy)-3,4,4a,5,6,9,10,11,12,12a-decahydro-1*H*-7,11-methanocyclodeca[3,4]benzo[1,2-*b*]oxete-6,12b(2a*H*)-diyl diacetate (7)**

Crude baccatin III (**6**) (23.4 g, assumed 36.7 mmol, 1.00 eq.) was dissolved in dry dichloromethane (438 mL) under nitrogen atmosphere. Dry pyridine (17.9 mL, 222 mmol, 6.04 eq.) was added, the reaction mixture was cooled to –78 °C and trifluoromethanesulfonic acid anhydride (12.4 mL, 73.4 mmol , 2.00 eq.) was added dropwise. 30 min after complete addition, the cooling bath was removed. Upon warming to room temperature, the reaction mixture turned black. After stirring for 90 min, the reaction was quenched with aqueous $NaHCO_3$ and extracted with dichloromethane. The combined organic phases were washed with brine and dried over $MgSO_4$. The solvents were evaporated, resulting in 27.2 g of an orange solid. The crude product was used in the next step without further purification and quantitative yield was assumed.

An analytically pure sample was obtained by column chromatography on silica (EtOAc/hexane, 4:1 v/v).

R_f = 0.54 (EtOAc/hexane = 3/1 (*v/v*), CAM).

$[\alpha]^D_{20}$: –90.38° (c=0.71, $CHCl_3$).

^{1}H NMR (400 MHz, $CDCl_3$): δ 8.19 – 8.02 (m, 2H), 7.66 – 7.58 (m, 1H), 7.53 – 7.45 (m, 2H), 6.62 (s, 1H), 5.67 (d, *J* = 6.9 Hz, 1H), 5.52 (dd, *J* = 10.2, 7.5 Hz, 1H), 4.94 (dd, *J* = 9.4, 1.7 Hz, 1H), 4.87 – 4.82 (m, 1H), 4.34 (d, *J* = 8.3 Hz, 1H), 4.15 (d, *J* = 8.5 Hz, 1H), 4.01 (d, *J* = 6.9 Hz, 1H), 2.87 (ddd, *J* = 14.4, 9.4, 7.5 Hz, 1H), 2.30 (s, 3H), 2.22 (d, *J* = 1.4 Hz, 3H), 2.19 (s, 3H), 2.33 – 2.14 (m, 3H), 1.86 (s, 3H), 1.64 (s, 1H), 1.60 (s, 1H), 1.19 (s, 3H), 1.05 (s, 3H).

^{19}F NMR (377 MHz, $CDCl_3$): δ -74.51.

^{13}C NMR (101 MHz, $CDCl_3$): δ 200.8, 171.2, 169.0, 167.1, 145.1, 134.0, 132.0, 130.2 (2C), 129.2, 128.9 (2C), 118.3 (q, *J* = 319 Hz), 86.3, 83.3, 79.8, 78.8, 76.4, 76.1, 74.2, 67.9, 57.7, 47.5, 42.7, 38.4, 34.3, 26.6, 22.6, 20.9, 20.1, 15.0, 10.9.

IR (film, cm^{-1}): 2980, 2925, 1709, 1392, 1378, 1266, 1242, 1208, 1142, 1071.

HRMS (ESI): *m/z* calcd for $C_{32}H_{37}F_3O_{13}SNa^+$ $[M+Na]^+$: 741.1799, found: 741.1798.

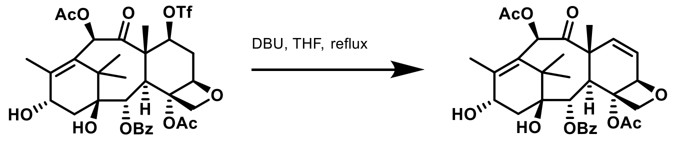

7 — Chemical Formula: $C_{32}H_{37}F_3O_{13}S$; Molecular Weight: 718,690

8 — Chemical Formula: $C_{31}H_{36}O_{10}$; Molecular Weight: 568,619

(2a*R*,4a*R*,6*R*,9*S*,11*S*,12*S*,12a*R*,12b*S*)-12-(benzoyloxy)-9,11-dihydroxy-4a,8,13,13-tetramethyl-5-oxo-4a,5,6,9,10,11,12,12a-octahydro-1*H*-7,11-methanocyclodeca[3,4]benzo[1,2-*b*]oxete-6,12b(2a*H*)-diyl diacetate (8)

Crude triflate **7** (27.2 g, assumed 36.7 mmol, 1.00 eq.) was dissolved in dry THF (700 mL) under nitrogen atmosphere. Dry DBU (27.6 mL, 178 mmol, 4.87 eq.) was added and the reaction mixture was heated to reflux for 14 h. After cooling to room temperature, aqueous NH_4Cl was added and the mixture was extracted with ethyl acetate. The combined organic phases were washed with brine, dried over $MgSO_4$ and the solvent was removed *in vacuo*, resulting in 26.1 g of a brown solid. The crude product was used in the next step without further purification and quantitative yield was assumed.

An analytically pure sample was obtained by column chromatography on silica (EtOAc/hexane, 4:1 v/v).

R_f = 0.49 (EtOAc/hexane = 3/1 (*v/v*), CAM).

$[\alpha]^D_{20}$: –141.96° (c=0.71, $CHCl_3$).

^{1}H NMR (400 MHz, $CDCl_3$): δ 8.17 – 8.11 (m, 2H), 7.66 – 7.58 (m, 1H), 7.53 – 7.45 (m, 2H), 6.24 (d, *J* = 2.2 Hz, 1H), 6.11 – 6.02 (m, 1H), 5.87 (dd, *J* = 10.0, 2.2 Hz, 1H), 5.80 (d, *J* = 6.7 Hz, 1H), 5.12 (d, *J* = 5.8 Hz, 1H), 4.89 – 4.85 (m, 1H), 4.43 (d, *J* = 8.4 Hz, 1H), 4.29 (d, *J* = 8.2 Hz, 1H), 4.10 (d, *J* = 6.7 Hz, 1H), 2.30 (s, 3H), 2.23 (s, 3H), 2.38 – 2.09 (m, 2H), 1.97 (s, 3H), 1.85 (s, 3H), 1.75 (s, 1H), 1.61 (s, 1H), 1.12 (s, 6H).

^{13}C NMR (101 MHz, $CDCl_3$): δ 205.8, 170.5, 169.8, 167.2, 145.6, 140.0, 133.9, 132.8, 130.3 (2C), 129.5, 128.8 (2C), 126.4, 81.4, 81.2, 78.9, 76.6, 76.6, 75.7, 68.1, 55.7, 42.8, 41.9, 39.2, 26.5, 22.9, 21.2, 21.0, 20.4, 15.1.

IR (film, cm^{-1}): 2980, 2927, 1716, 1451, 1372, 1269, 1230, 1177, 1150, 1109, 1071, 1050, 1025.

HRMS (ESI): *m/z* calcd for $C_{31}H_{37}O_{10}^+$ $[M+H]^+$: 569.2381, found: 569.2381.

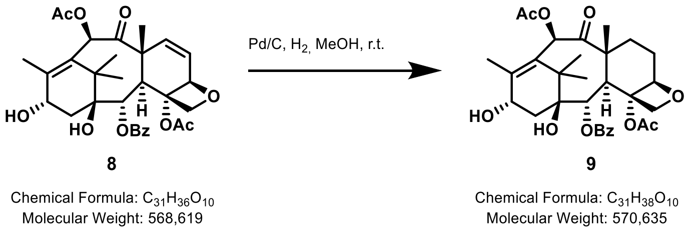

(2a*R*,4a*R*,6*R*,9*S*,11*S*,12*S*,12a*R*,12b*S*)-12-(benzoyloxy)-9,11-dihydroxy-4a,8,13,13-tetramethyl-5-oxo-3,4,4a,5,6,9,10,11,12,12a-decahydro-1*H*-7,11-methanocyclodeca[3,4]benzo[1,2-*b*]oxete-6,12b(2a*H*)-diyl diacetate (9)

Crude olefin **8** (26.1 g, assumed 36.7 mmol, 1.00 eq.) was dissolved in methanol (800 mL) under nitrogen atmosphere at room temperature. Palladium on charcoal (10% Pd/C, 2.45 g) was added and the atmosphere was exchanged to hydrogen (ballon pressure). Reaction progress was monitored *via* ^{1}H-NMR aliquots. After 24 h, the atmosphere was exchanged to nitrogen. The reaction mixture was diluted with ethyl acetate, filtered over celite and the solvent was evaporated. The crude product (25.8 g) was obtained as white foam and used in the next step without further purification. Quantitative yield was assumed.

An analytically pure sample was obtained by column chromatography on silica (EtOAc/hexane, 1:1 v/v to 3:1 v/v) .

R_f = 0.50 (EtOAc/hexane = 3/1 (*v/v*), CAM).

$[\alpha]^{D}_{20}$: –107.36° (c=0.71, $CHCl_3$).

^{1}H NMR (400 MHz, $CDCl_3$): δ 8.15 – 8.08 (m, 2H), 7.65 – 7.56 (m, 1H), 7.52 – 7.44 (m, 2H), 6.48 (s, 1H), 5.62 (d, *J* = 7.3 Hz, 1H), 4.96 (dd, *J* = 9.4, 2.6 Hz, 1H), 4.87 – 4.83 (m, 1H), 4.31 (d, *J* = 8.8 Hz, 1H), 4.19 (d, *J* = 8.3 Hz, 1H), 3.85 (d, *J* = 7.3 Hz, 1H), 2.36 – 2.13 (m, 4H), 2.28 (s, 3H), 2.21 (s, 3H), 2.05 (d, *J* = 1.4 Hz, 3H), 1.99 – 1.86 (m, 1H), 1.73 (s, 3H), 1.69 (s, 1H), 1.61 (s, 1H), 1.60 – 1.52 (m, 1H), 1.11 (s, 3H), 1.08 (s, 3H).

^{13}C NMR (101 MHz, $CDCl_3$): δ 206.7, 170.7, 169.9, 167.4, 144.6, 133.8, 132.4, 130.3 (2C), 129.7, 128.8 (2C), 84.7, 82.0, 79.2, 76.7, 75.9, 75.0, 68.0, 53.1, 45.6, 42.7, 38.9, 35.2, 27.2, 26.6, 22.8, 21.0, 20.7, 15.2, 14.8.

IR (film, cm^{-1}): 2980, 2970, 2927, 1713, 1462, 1380, 1239, 1150, 1070, 1021.

HRMS (ESI): *m/z* calcd for $C_{31}H_{39}O_{10}^{+}$ [*M*+H]$^{+}$: 571.2538, found: 571.2537.

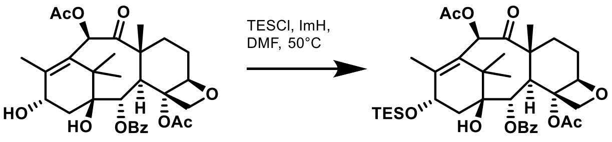

9

Chemical Formula: $C_{31}H_{38}O_{10}$
Molecular Weight: 570,635

10

Chemical Formula: $C_{37}H_{52}O_{10}Si$
Molecular Weight: 684,898

(2a*R*,4a*R*,6*R*,9*S*,11*S*,12*S*,12a*R*,12b*S*)-12-(benzoyloxy)-11-hydroxy-4a,8,13,13-tetramethyl-5-oxo-9-((triethylsilyl)oxy)-3,4,4a,5,6,9,10,11,12,12a-decahydro-1*H*-7,11-methanocyclodeca[3,4]benzo[1,2-*b*]oxete-6,12b(2a*H*)-diyl diacetate (10)

Crude compound **9** (25.8 g, assumed 36.7 mmol, 1.00 eq.) and imidazole (12.5 g, 184 mmol, 5.00 eq.) were dissolved in dry DMF (150 mL) at room temperature. TESCl (24.7 mL, 147 mmol, 4.00 eq.) was added and the mixture was heated to 50 °C for 16 h. The reaction mixture was diluted with ethyl acetate and washed with brine. The aqueous phase was extracted with ethyl acetate and the combined organic phases were washed with brine, dried over $MgSO_4$ and the solvent was removed *in vacuo*. The residue was recrystallized from dichloromethane/hexane, yielding the desired product as a white solid in 61% yield over five steps (15.4 g, 22.5 mmol).

R_f = 0.79 (hexane/EtOAc = 1/1 (*v/v*), CAM).

$[\alpha]^D_{20}$: –57.53° (c=0.69, $CHCl_3$).

^{1}H NMR (400 MHz, $CDCl_3$): δ 8.13 – 8.05 (m, 2H), 7.64 – 7.56 (m, 1H), 7.51 – 7.43 (m, 2H), 6.46 (s, 1H), 5.65 – 5.55 (m, 1H), 4.96 (dd, *J* = 9.4, 2.8 Hz, 1H), 4.95 – 4.87 (m, 1H), 4.30 (d, *J* = 8.5 Hz, 1H), 4.18 (d, *J* = 8.1 Hz, 1H), 3.76 (d, *J* = 7.3 Hz, 1H), 2.28 (s, 3H), 2.20 (s, 3H), 2.33 – 2.08 (m, 4H), 1.99 (s, 3H), 1.96 – 1.87 (m, 1H), 1.72 (s, 3H), 1.71 (s, 1H), 1.59 – 1.49 (m, 1H), 1.13 (s, 3H), 1.10 (s, 3H), 1.01 (t, *J* = 7.9 Hz, 9H), 0.67 (q, *J* = 7.6 Hz, 6H).

^{13}C NMR (101 MHz, $CDCl_3$): δ 207.0, 170.1, 169.8, 167.3, 146.3, 133.7, 131.4, 130.2 (2C), 129.7, 128.8, 128.7, 84.6, 81.9, 79.9, 76.6, 76.4, 75.0, 68.5, 52.7, 45.3, 42.8, 40.6, 35.4, 27.1, 26.3, 22.5, 21.7, 21.0, 15.1, 14.9, 7.0 (3C), 5.0, 4.9 (2C).

IR (film, cm^{-1}): 2980, 1732, 1713, 1451, 1368, 1269, 1236, 1176, 1110, 1084, 1069, 1017.

HRMS (ESI): *m/z* calcd for $C_{37}H_{53}O_{10}Si^+$ $[M+H]^+$: 685.3403, found: 685.3402.

Notes:

*Crude compound **9** contains a minor impurity, which reacts similarly to the main compound and stays inseparable in compound **10**. This is the case for the next 4 steps. This impurity can finally be removed after the Corey-Winter elimination, which delivers pure compound **14** (vide infra).*

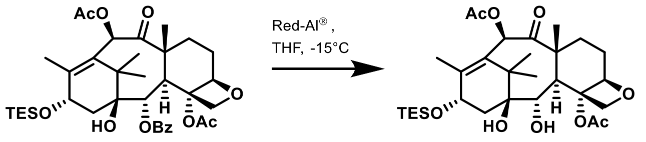

10 | **11**

Chemical Formula: $C_{37}H_{52}O_{10}Si$
Molecular Weight: 684,898

Chemical Formula: $C_{30}H_{48}O_9Si$
Molecular Weight: 580,790

(2a*R*,4a*R*,6*R*,9*S*,11*S*,12*S*,12a*R*,12b*S*)-11,12-dihydroxy-4a,8,13,13-tetramethyl-5-oxo-9-((triethylsilyl)oxy)-3,4,4a,5,6,9,10,11,12,12a-decahydro-1*H*-7,11-methanocyclodeca[3,4]benzo[1,2-*b*]oxete-6,12b(2a*H*)-diyl diacetate (11)

Benzoate **10** (24.3 g, 35.5 mmol, 1.00 eq.) was dissolved in dry THF (712 mL) and cooled to –15 °C. Red-Al® (3M in toluene, 26.4 mL, 79.2 mmol, 2.23 eq.) was added dropwise and the reaction was stirred for 90 min. The reaction was quenched with aqueous Rochelle salt solution at –15 °C under vigorous stirring and the cooling bath was removed. The mixture was extracted with ethyl acetate, the combined organic phases were washed with brine and dried over $MgSO_4$. The crude product (24.4 g) was used in the next step without further purification and quantitative yield was assumed.

An analytically pure sample was obtained by column chromatography on silica (hexane/EtOAc, 2:1 v/v to 1:2 v/v).

R_f = 0.34 (hexane/EtOAc = 1/1 (*v/v*), CAM).

$[\alpha]^D_{20}$: –68.95° (c=0.57, $CHCl_3$).

^{1}H NMR (400 MHz, $CDCl_3$): δ 6.39 (s, 1H), 4.98 (d, *J* = 9.1 Hz, 1H), 4.96 – 4.91 (m, 1H), 4.63 (d, *J* = 9.1 Hz, 1H), 4.58 (d, *J* = 9.0 Hz, 1H), 3.89 – 3.81 (m, 1H), 3.34 (d, *J* = 7.1 Hz, 1H), 2.56 (s, 1H), 2.43 – 2.38 (m, 1H), 2.34 – 2.22 (m, 1H), 2.18 (s, 3H), 2.16 (s, 3H), 2.18 – 1.97 (m, 4H), 1.93 (s, 3H), 1.67 (s, 3H), 1.56 – 1.46 (m, 1H), 1.17 (s, 3H), 1.00 (s, 3H), 0.99 (t, *J* = 8.0 Hz, 9H), 0.65 (q, *J* = 7.7 Hz, 6H).

^{13}C NMR (101 MHz, $CDCl_3$): δ 207.5, 169.9, 169.6, 145.8, 131.7, 84.1, 83.4, 78.8, 78.0, 76.1, 75.1, 68.6, 52.7, 45.3, 42.2, 41.3, 35.5, 27.2, 25.9, 22.4, 21.7, 21.0, 15.1, 15.0, 7.1 (3C), 5.0 (3C).

IR (film, cm^{-1}): 2980, 2878, 1728, 1708, 1456, 1369, 1227, 1145, 1083, 1063, 1016.

HRMS (ESI): *m/z* calcd for $C_{30}H_{49}O_9Si^+$ $[M+H]^+$: 581.3140, found: 581.3137.

Notes:

*Compound **10** contains a minor inseparable impurity, which reacts similarly to the main compound and stays inseparable in compound **11**. This is the case for the next 3 steps. This impurity can finally be removed after the Corey-Winter elimination, which delivers pure compound **14** (vide infra).*

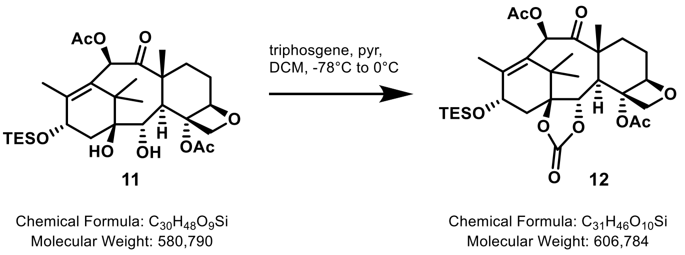

(3a*S*,5*S*,8*R*,9a*R*,11a*R*,13a*S*,13b*R*,13c*S*)-6,9a,14,14-tetramethyl-2,9-dioxo-5-((triethylsilyl)oxy)-4,5,8,9,9a,10,11,11a,13b,13c-decahydro-3a,7-methanooxeto[2'',3'':5',6']benzo[1',2':3,4]-cyclodeca[1,2-*d*][1,3]dioxole-8,13a(13*H*)-diyl diacetate (12)

Crude diol **11** (24.4 g, assumed 35.5 mmol, 1.00 eq.) was dissolved in dry dichloromethane (838 mL) under nitrogen atmosphere, dry pyridine (33.6 mL, 416 mmol, 11.7 eq.) was added and the reaction mixture was cooled to –78 °C. Triphosgene (16.3 g, 54.9 mmol, 1.55 eq.) was added and the temperature was subsequently raised to 0 °C. The reaction was stirred for 3 h, quenched with aqueous $NaHCO_3$ and extracted with dichloromethane. The combined organic phases were washed with brine, dried over $MgSO_4$, the solvents were removed *in vacuo* and the crude product was coevaporated twice with toluene to remove residual pyridine. Column chromatography on silica gel (hexane/EtOAc, 3:1 *v*/*v* to 1:1 *v/v*) gave the desired product as white powder in 87% yield over two steps (18.6 g, 30.7 mmol).

R_f = 0.81 (hexane/EtOAc = 1/1 (*v/v*), CAM).

$[\alpha]^D_{20}$: –66.69° (c=1.14, $CHCl_3$).

^{1}H NMR (400 MHz, $CDCl_3$): δ 6.43 (s, 1H), 5.03 – 4.93 (m, 2H), 4.62 (d, *J* = 9.0 Hz, 1H), 4.50 (d, *J* = 6.1 Hz, 1H), 4.44 (d, *J* = 8.9 Hz, 1H), 3.37 (d, *J* = 6.0 Hz, 1H), 2.41 (dd, *J* = 15.3, 8.9 Hz, 1H), 2.35 – 2.25 (m, 1H), 2.18 (s, 3H), 2.14 (s, 3H), 2.02 (s, 3H), 2.21 – 1.90 (m, 3H), 1.74 (s, 3H), 1.67 – 1.56 (m, 1H), 1.29 (s, 3H), 1.14 (s, 3H), 0.99 (t, *J* = 7.3 Hz, 9H), 0.65 (q, *J* = 8.0 Hz, 6H).

^{13}C NMR (101 MHz, $CDCl_3$): δ 206.4, 170.2, 169.6, 153.4, 149.5, 129.4, 90.6, 83.7, 82.7, 80.4, 76.4, 75.8, 67.6, 54.3, 42.8, 40.9, 37.4, 33.1, 27.2, 25.2, 22.4, 21.4, 20.9, 15.7, 14.7, 7.0 (3C), 4.9 (3C).

IR (film, cm^{-1}): 2980, 2880, 1812, 1736, 1706, 1369, 1225, 1201, 1151, 1073, 1014, 1002.

HRMS (ESI): *m/z* calcd for $C_{31}H_{47}O_{10}Si^+$ [*M*+H]$^+$: 607.2933, found: 607.2927.

Notes:

*Compound **11** contains a minor inseparable impurity, which reacts similarly to the main compound and stays inseparable in compound **12**. This is the case for the next 2 steps. This impurity can finally be removed after the Corey-Winter elimination, which delivers pure compound **14** (vide infra).*

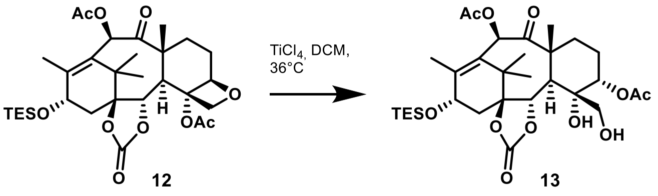

Chemical Formula: $C_{31}H_{46}O_{10}Si$
Molecular Weight: 606,784

Chemical Formula: $C_{31}H_{48}O_{11}Si$
Molecular Weight: 624,799

(3a*S*,5*S*,8*R*,9a*R*,12*S*,13*S*,13a*R*,13b*S*)-13-hydroxy-13-(hydroxymethyl)-6,9a,14,14-tetramethyl-2,9-dioxo-5-((triethylsilyl)oxy)-5,8,9,9a,10,11,12,13,13a,13b-decahydro-4*H*-3a,7-methanobenzo[3,4]cyclodeca[1,2-*d*][1,3]dioxole-8,12-diyl diacetate (13)

Carbonate **12** (1.08 g, 1.78 mmol, 1.00 eq.) was dissolved in dry dichloromethane (2.70 L) and warmed to 36 °C. Titanium tetrachloride (1M in DCM, 1.78 mL, 1.78 mmol, 1.00 eq.) was added in one portion under vigorous stirring and the reaction was stirred for 10 min. The reaction was quenched with a mixture of aqueous $NaHCO_3$ (800 mL) and Rochelle salt solution (100 ml). The phases were separated and the organic phase was washed with brine and dried over $MgSO_4$. The solvent was evaporated and the crude product was purified *via* column chromatography on silica gel (hexane/EtOAc, 3:1 *v/v* to 1:1 *v/v*) to yield the desired product as a white solid (818 mg, 1.31 mmol, 74%).

R_f = 0.46 (hexane/EtOAc = 1/1 (*v/v*), CAM).

$[\alpha]^D_{20}$: –36.48° (c=0.59, $CHCl_3$).

^{1}H NMR (400 MHz, $CDCl_3$): δ 6.55 (s, 1H), 5.25 (d, *J* = 3.0 Hz, 1H), 5.01 – 4.94 (m, 1H), 4.29 (d, *J* = 4.9 Hz, 1H), 4.07 (dd, *J* = 10.9, 4.6 Hz, 1H), 3.57 (dd, *J* = 11.2, 7.4 Hz, 1H), 3.40 (d, *J* = 4.9 Hz, 1H), 3.19 (s, 1H), 3.19 (dd, *J* = 15.2, 6.5 Hz, 1H), 2.37 (dd, *J* = 15.0, 9.4 Hz, 1H), 2.21 (d, *J* = 1.5 Hz, 3H), 2.20 (s, 3H), 2.18 (s, 3H), 1.94 – 1.86 (m, 3H), 1.26 (s, 3H), 1.26 (s, 3H), 1.14 (s, 1H), 1.11 (s, 3H), 0.98 (t, *J* = 7.9 Hz, 9H), 0.66 (q, *J* = 8.2 Hz, 6H).

^{13}C NMR (101 MHz, $CDCl_3$): δ 205.9, 170.9, 169.8, 153.7, 148.9, 130.0, 90.9, 83.5, 75.5, 74.6, 71.6, 68.5, 63.3, 55.6, 44.7, 41.1, 36.7, 31.1, 25.7, 23.9, 21.7, 20.9, 20.7, 17.3, 16.1, 6.9 (3C), 4.9 (3C).

IR (film, cm^{-1}): 2979, 2879, 1805, 1744, 1705, 1370, 1229, 1093, 1046, 1009.

HRMS (ESI): *m/z* calcd for $C_{31}H_{48}O_{11}SiNa^+$ $[M+Na]^+$: 647.2858, found: 647.2856.

Notes:

*Compound **12** contains a minor inseparable impurity, which reacts similarly to the main compound and stays inseparable in compound **13**. This is also the case for the next step. This impurity can finally be removed after the Corey-Winter elimination, which delivers pure compound **14** (vide infra).*

Notes:

While best results are obtained in extreme dilution (e.g. 0.00066 M as described above), it is possible to conduct the reaction in a higher concentration at the cost of decreased yields. If the reaction was conducted in tenfold concentration (~0.006 m) on decagram scale, yields around 45-50% were observed.

*The $TiCl_4$-mediated oxetane opening is highly temperature sensitive. Strict temperature control with an internal thermometer is crucial to ensure reproducible results and to supress the formation of regioisomeric side product **S1** (see below).*

*Prolonged reaction times lead to increased decomposition, as well as formation of **S1**.*

Due to a high dilution and short reaction times, the temperature was assumed to be constant on larger scales throughout the reaction. In practice, the solvent was heated to reflux (40 °C) and the heat source was subsequently removed. When the internal temperature fell to 36 °C, $TiCl_4$ was added in one portion and a timer was started.

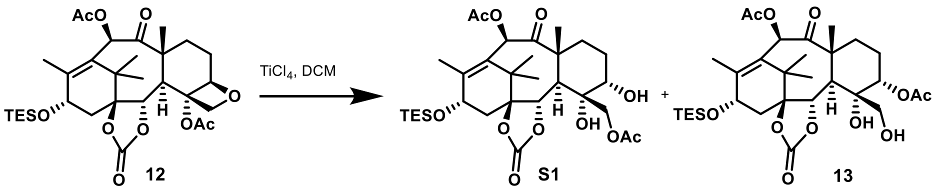

	temperature	**S1** : **13**
1	0°C	4 : 1
2	r.t.	2 : 1
3	30°C	1 : 5
4	36°C	1: 27
5	reflux (40°C)	decomposition

((3aS,5S,8R,9aR,12S,13S,13aR,13bS)-8-acetoxy-12,13-dihydroxy-6,9a,14,14-tetramethyl-2,9-dioxo-5-((triethylsilyl)oxy)-5,8,9,9a,10,11,12,13,13a,13b-decahydro-4H-3a,7-methanobenzo[3,4]cyclodeca [1,2-d][1,3]dioxol-13-yl)methyl acetate (S1)

***R_f** = 0.69 (hexane/EtOAc = 1/1 (v/v), CAM).*

***^{1}H NMR** (400 MHz, $CDCl_3$): δ 6.61 (s, 1H), 4.91 – 4.76 (m, 1H), 4.54 (d, J = 11.8 Hz, 1H), 4.36 (d, J = 11.8 Hz, 1H), 4.23 (d, J = 4.9 Hz, 1H), 3.73 – 3.67 (m, 2H), 3.20 (dd, J = 15.3, 4.6 Hz, 1H), 3.08 (s, 1H), 2.97 (d, J = 2.0 Hz, 1H), 2.42 (dd, J = 15.2, 9.7 Hz, 1H), 2.18 (d, J = 1.5 Hz, 3H), 2.17 (s, 3H), 2.13 (s, 3H), 2.10 (s, 1H), 1.98 – 1.89 (m, 1H), 1.83 – 1.72 (m, 1H), 1.30 (s, 3H), 1.19 (s, 3H), 1.21 – 1.14 (m, 1H), 1.11 (s, 3H), 1.00 (t, J = 7.9 Hz, 9H), 0.67 (q, J = 7.9 Hz, 6H).*

***^{13}C NMR** (101 MHz, $CDCl_3$): δ 205.4, 170.6, 169.7, 153.4, 149.1, 129.9, 91.0, 82.9, 75.8, 75.8, 69.4, 68.6, 64.9, 56.0, 42.9, 40.8, 36.4, 29.4, 26.5, 24.7, 20.9 (2C), 19.7, 17.1, 16.9, 7.0 (3C), 5.0 (3C).*

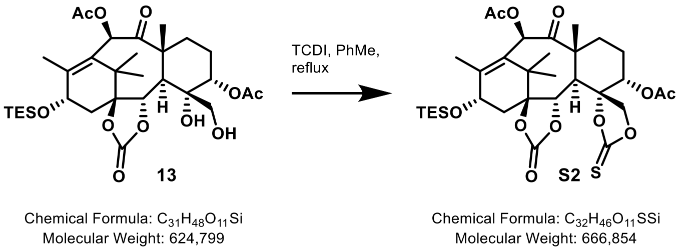

(3a'*S*,4*S*,5'*S*,8'*R*,9a'*R*,12'*S*,13a'*R*,13b'*S*)-6',9a',14',14'-tetramethyl-2',9'-dioxo-2-thioxo-5'-((triethylsilyl)oxy)-4',5',8',9',9a',10',11',12',13a',13b'-decahydrospiro[[1,3]dioxolane-4,13'-[3a,7]methanobenzo[3,4]cyclodeca[1,2-*d*][1,3]dioxole]-8',12'-diyl diacetate (S2)

Diol **13** (5.23 g, 8.36 mmol. 1.00 eq.) and thiocarbonyl diimidazole (5.96 g, 33.4 mmol, 4.00 eq.) were dissolved in dry toluene (330 mL) and the reaction mixture was heated to reflux for 2 h. After letting the mixture cool to room temperature, the solvent was evaporated and the residue was subjected to column chromatography on silica gel (hexane/EtOAc, 1:1 *v/v*) to yield the desired product as a light-yellow solid (4.99 g, 7.48 mmol, 89%).

R_f = 0.48 (hexane/EtOAc = 1/1 (*v/v*), CAM).

$[\alpha]^D_{20}$: –33.06° (c=0.57, $CHCl_3$).

^{1}H NMR (400 MHz, $CDCl_3$): δ 6.56 (s, 1H), 5.28 – 5.23 (m, 1H), 5.08 – 4.99 (m, 1H), 4.73 (d, *J* = 9.0 Hz, 1H), 4.37 (d, *J* = 5.4 Hz, 1H), 4.30 (d, *J* = 9.1 Hz, 1H), 3.74 (d, *J* = 5.4 Hz, 1H), 2.80 (dd, *J* = 15.5, 6.9 Hz, 1H), 2.50 (dd, *J* = 15.4, 9.1 Hz, 1H), 2.24 (s, 6H), 2.19 (s, 3H), 2.02 – 1.85 (m, 3H), 1.41 – 1.33 (m, 1H), 1.30 (s, 3H), 1.19 (s, 3H), 1.14 (s, 3H), 1.01 (t, *J* = 8.0 Hz, 9H), 0.69 (q, *J* = 7.7 Hz, 6H).

^{13}C NMR (101 MHz, $CDCl_3$): δ 204.2, 188.1, 169.7, 169.3, 152.1, 149.9, 129.5, 90.5, 87.7, 81.7, 75.5, 74.0, 70.4, 68.2, 55.0, 41.4, 41.2, 36.9, 30.1, 25.4, 23.7, 21.2, 20.9, 20.8, 16.5, 16.1, 7.1 (3C), 4.9 (3C).

IR (film, cm^{-1}): 2980, 2970, 2925, 1462, 1381, 1250, 1151, 1072.

HRMS (ESI): *m/z* calcd for $C_{32}H_{46}O_{11}SSiNa^+$ $[M+Na]^+$: 689.2422, found: 689.2425.

Notes:

*Compound **13** contains a minor inseparable impurity, which reacts similarly to the main compound and stays inseparable in compound **S2**. This impurity can finally be removed after the Corey-Winter elimination, which delivers pure compound **14** (vide infra).*

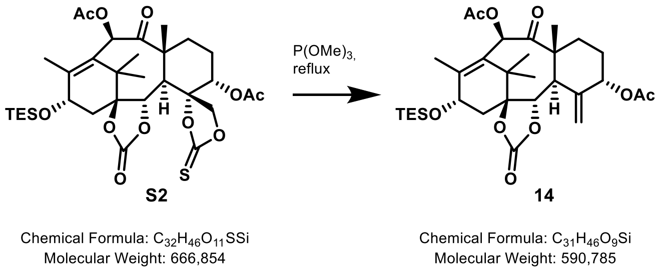

(3a*S*,5*S*,8*R*,9a*R*,12*S*,13a*R*,13b*S*)-6,9a,14,14-tetramethyl-13-methylene-2,9-dioxo-5-((triethylsilyl)oxy)-5,8,9,9a,10,11,12,13,13a,13b-decahydro-4*H*-3a,7-methanobenzo[3,4]cyclodeca[1,2-*d*][1,3]dioxole-8,12-diyl diacetate (14)

Thiocarbonate **S2** (4.99 g, 7.48 mmol, 1.00 eq.) was dissolved in trimethyl phosphite (88.4 mL, 748 mmol, 100 eq.) under nitrogen atmosphere and the reaction mixture was heated to reflux for 66 h. The solvent was evaporated at 60 °C under reduced pressure and the residue was subjected to column chromatography on silica gel (hexane/EtOAc, 5:1 *v/v* to 2:1 *v/v*) to yield the desired product as a white solid (3.84 g, 6.49 mmol, 87%).

R_f = 0.64 (hexane/EtOAc = 2/1 (*v/v*), CAM).

$[\alpha]^D_{20}$: –66.13° (c=0.57, $CHCl_3$).

^{1}H NMR (400 MHz, $CDCl_3$): δ 6.61 (s, 1H), 5.67 (s, 1H), 5.35 (s, 1H), 5.34 (d, *J* = 2.9 Hz, 1H), 5.09 (t, *J* = 7.9 Hz, 1H), 4.38 (d, *J* = 5.7 Hz, 1H), 3.74 (d, *J* = 5.6 Hz, 1H), 2.47 (dd, *J* = 15.1, 8.9 Hz, 1H), 2.23 (s, 3H), 2.20 (s, 3H), 2.25 – 2.14 (m, 1H), 2.16 (s, 3H), 2.13 – 2.01 (m, 1H), 1.95 – 1.81 (m, 2H), 1.43 – 1.33 (m, 1H), 1.31 (s, 3H), 1.25 (s, 3H), 1.18 (s, 3H), 0.97 (t, *J* = 7.9 Hz, 9H), 0.65 (q, *J* = 8.0 Hz, 6H).

^{13}C NMR (101 MHz, $CDCl_3$): δ 206.3, 169.9, 169.8, 153.3, 148.5, 139.3, 129.0, 119.6, 90.8, 83.6, 76.7, 75.6, 68.3, 56.6, 44.2, 41.1, 38.3, 32.4, 27.7, 25.9, 21.7, 21.0, 21.0, 16.0, 15.9, 7.0 (3C), 4.9 (3C).

IR (film, cm^{-1}): 2955, 2876, 1810, 1742, 1707, 1370, 1226, 1198, 1112, 1047, 1015.

HRMS (ESI): *m/z* calcd for $C_{31}H_{47}O_9Si^+$ $[M+H]^+$: 591.2984, found: 591.2985.

Notes:

*C7-acetate **S3** was obtained as a minor side product. Careful analysis of the impure spectra of starting material **S2** as well as precursors **9**, **10**, **11**, **12** and **13** hints towards the corresponding C7-acetates as the respective contaminants in each of these steps. Presumably, this is a result of minor C7,C10-bisacetylation leading to bisacetate **S4** in the large-scale C10-acetylation of 10-DAB (**2**) in the very first step. All chromatographic separations which followed failed to separate this impurity, due to*

*indistinguishable R_f-values of the desired C7-methylene derivatives and the respective C7-acetate. The chromatographic purification of compound **14** finally enables the removal of this side product.*

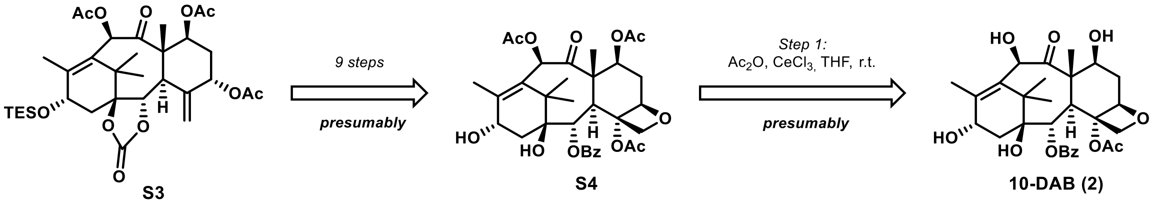

S3 S4 10-DAB (2)

*Impurity **S3** can be easily identified and integrated in the product mixture via the corresponding C7-H NMR signal: 5.54 (dd, J = 11.5, 4.9 Hz, 1H). Similar signals exist in the precursor impurities.*

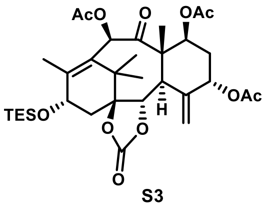

S3

Chemical Formula: $C_{33}H_{48}O_{11}Si$
Molecular Weight: 648,821

(3aS,5S,8R,9aS,10S,12S,13aR,13bS)-6,9a,14,14-tetramethyl-13-methylene-2,9-dioxo-5-((triethyl silyl)oxy)-5,8,9,9a,10,11,12,13,13a,13b-decahydro-4H-3a,7-methanobenzo[3,4]cyclodeca[1,2-d][1,3]dioxole-8,10,12-triyl triacetate (S3)

***R_f** = 0.58 (hexane/EtOAc = 2/1 (v/v), CAM).*

***^{1}H NMR** (400 MHz, $CDCl_3$): δ 6.34 (s, 1H), 5.73 (d, J = 1.7 Hz, 1H), 5.54 (dd, J = 11.5, 4.9 Hz, 1H), 5.40 (dd, J = 5.5, 2.2 Hz, 2H), 5.17 – 5.08 (m, 1H), 4.33 (d, J = 5.8 Hz, 1H), 3.70 (d, J = 5.7 Hz, 1H), 2.46 (dd, J = 15.2, 8.8 Hz, 1H), 2.27 (s, 3H), 2.19 (s, 3H), 2.15 (s, 3H), 2.22 – 2.07 (m, 2H), 2.03 (s, 3H), 1.77 (ddd, J = 14.0, 11.6, 3.7 Hz, 1H), 1.33 (s, 3H), 1.25 (s, 3H), 1.18 (s, 3H), 0.98 (t, J = 7.9 Hz, 9H), 0.65 (q, J = 7.9 Hz, 6H).*

***^{13}C NMR** (101 MHz, $CDCl_3$): δ 203.5, 170.7, 169.8, 169.2, 153.0, 149.0, 137.6, 127.9, 121.2, 90.9, 82.1, 76.6, 75.7, 69.7, 68.4, 59.1, 44.8, 41.4, 38.2, 33.2, 26.3, 21.7, 21.0, 20.9, 20.8, 16.3, 11.7, 7.0 (3C), 4.9 (3C).*

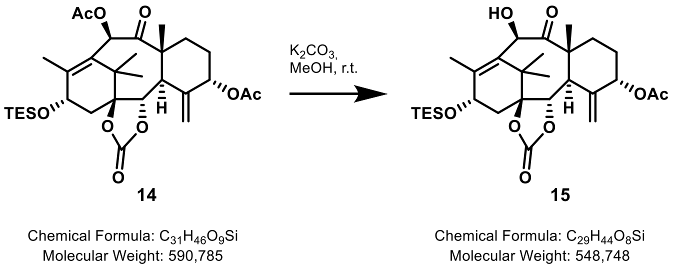

(3a*S*,5*S*,8*R*,9a*R*,12*S*,13a*R*,13b*S*)-8-hydroxy-6,9a,14,14-tetramethyl-13-methylene-2,9-dioxo-5-((triethylsilyl)oxy)-5,8,9,9a,10,11,12,13,13a,13b-decahydro-4*H*-3a,7-methanobenzo[3,4]-cyclodeca[1,2-*d*][1,3]dioxol-12-yl acetate (15)

Compound **14** (2.01 g, 3.40 mmol, 1.00 eq.) was dissolved in dry methanol (200 mL) under nitrogen atmosphere. Potassium carbonate (494 mg, 3.57 mmol, 1.05 eq.) was added and the reaction was stirred for 45 min at room temperature. The reaction mixture was diluted with dichloromethane and quenched with aqueous NH_4Cl. The phases were separated, and the aqueous phase was extracted with dichloromethane. The combined organic phases were washed with brine, dried over $MgSO_4$ and the solvent was removed under reduced pressure. Column chromatography on silica gel (hexane/EtOAc, 4:1 *v/v* to 2:1 *v/v*) gave the desired product as a white solid (1.52 g, 2.77 mmol) in 81% yield.

R_f = 0.49 (hexane/EtOAc = 2/1 (*v/v*), CAM).

$[\alpha]^D_{20}$: –39.60° (c=0.50, $CHCl_3$).

^{1}H NMR (400 MHz, $CDCl_3$): δ 5.68 (s, 1H), 5.38 (d, *J* = 1.9 Hz, 1H), 5.36 (s, 1H), 5.34 – 5.31 (m, 1H), 5.10 (t, *J* = 8.2 Hz, 1H), 4.36 (d, *J* = 5.7 Hz, 1H), 4.07 (d, *J* = 1.9 Hz, 1H), 3.81 (d, *J* = 5.7 Hz, 1H), 2.44 (dd, *J* = 14.9, 8.9 Hz, 1H), 2.18 (s, 3H), 2.21 – 2.16 (m, 1H), 2.14 (s, 3H), 2.05 – 1.93 (m, 1H), 1.92 – 1.84 (m, 2H), 1.37 – 1.32 (m, 1H), 1.32 (s, 3H), 1.31 (s, 3H), 1.10 (s, 3H), 0.97 (t, *J* = 7.9 Hz, 9H), 0.64 (q, *J* = 8.5, 8.1 Hz, 6H).

^{13}C NMR (101 MHz, $CDCl_3$): δ 214.7, 169.9, 153.3, 146.1, 139.1, 131.5, 119.8, 91.0, 83.6, 76.6, 75.0, 68.2, 56.2, 44.2, 40.9, 38.4, 33.2, 27.7, 25.7, 21.7, 20.6, 15.9, 15.7, 7.0 (3C), 4.9 (3C).

IR (film, cm^{-1}): 2954, 2876, 1808, 1740, 1684, 1368, 1231, 1198, 1086, 1045, 1010.

HRMS (ESI): *m/z* calcd for $C_{29}H_{45}O_8Si^+$ $[M+H]^+$: 549.2878, found: 549.2880.

Notes:

*Occasionally, formation of diketone **S5** was observed. While usually obtained in negligible amounts (0%-5%), sporadically over 50% of the material was converted to **S5**. Although the mechanism of this oxidation is unknown, the high variance in the amount of **S5** formed and the lack of oxidative species in the reaction mixture hint towards molecular oxygen as oxidant. To supress **S5**-formation, the reaction time should be kept to a minimum. Monitoring via TLC is possible, however it should be noted that the R_f-values of starting material **14** and oxidized product **S5** are quite similar. After workup, the desired hydroxy ketone **15** is stable and can be purified and stored without any signs of further oxidation.*

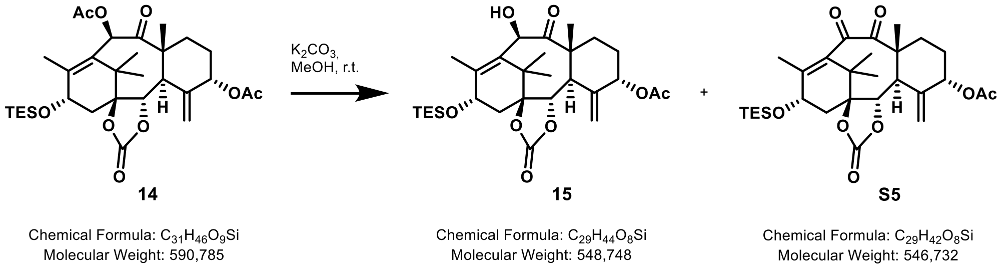

(3aS,5S,9aR,12S,13aR,13bS)-6,9a,14,14-tetramethyl-13-methylene-2,8,9-trioxo-5-(triethylsilyloxy)-5,8,9,9a,10,11,12,13,13a,13b-decahydro-4H-3a,7-methanobenzo[3,4]cyclodeca[1,2-d][1,3]dioxol-12-yl acetate (S5):

***R_f** = 0.67 (hexane/EtOAc = 2/1 (v/v), CAM).*

***^{1}H NMR** (400 MHz, $CDCl_3$): δ 5.69 (d, J = 1.7 Hz, 1H), 5.40 (bs, J = 1.4 Hz, 1H), 5.34 – 5.31 (m, 1H), 5.16 – 5.09 (m, 1H), 4.57 (d, J = 5.8 Hz, 1H), 3.67 (d, J = 5.8 Hz, 1H), 2.51 (dd, J = 15.2, 8.8 Hz, 1H), 2.26 (dd, J = 15.2, 7.7 Hz, 1H), 2.12 (s, 3H), 2.11 (d, J = 1.5 Hz, 3H), 1.93 – 1.82 (m, 2H), 1.82 – 1.74 (m, 1H), 1.35 (s, 3H), 1.30 (s, 3H), 1.25 – 1.20 (m, 1H), 1.19 (s, 3H), 0.97 (t, J = 7.9 Hz, 9H), 0.65 (q, J = 7.8 Hz, 6H).*

***^{13}C NMR** (101 MHz, $CDCl_3$): δ 207.2, 194.2, 169.5, 152.8, 150.9, 138.9, 138.4, 120.1, 90.6, 83.3, 76.3, 68.4, 56.3, 43.6, 39.0, 38.4, 30.9, 27.5, 26.1, 22.0, 21.7, 15.3, 14.5, 6.9 (3C), 4.8 (3C).*

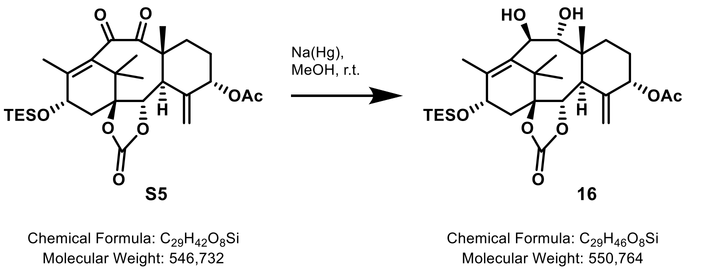

*To our delight, diketone **S5** can be converted to the desired trans-diol **16** by treatment with excess Na(Hg) via the same procedure as used for the reduction of hydroxy-ketone **15** (vide infra). Notably, this reaction proceeds with full diastereoselectivity and >90% yield, enabling a convergent synthesis of **16** by parallel or combined reduction of **15** and **S5**.*

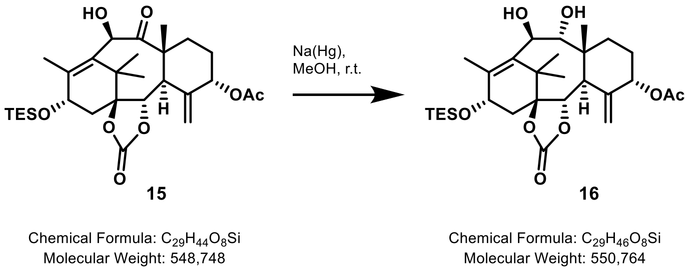

(3a*S*,5*S*,8*R*,9*R*,9a*R*,12*S*,13a*R*,13b*S*)-8,9-dihydroxy-6,9a,14,14-tetramethyl-13-methylene-2-oxo-5-((triethylsilyl)oxy)-5,8,9,9a,10,11,12,13,13a,13b-decahydro-4*H*-3a,7-methanobenzo[3,4]-cyclodeca[1,2-*d*][1,3]dioxol-12-yl acetate (16)

Compound **15** (1.28 g, 2.33 mmol, 1.00 eq.) was dissolved in dry methanol (139 mL) and sodium amalgam (2.5 wt.% Na/Hg, 21.4 g, 23.3 mmol, 10.0 eq.) was added. The reaction mixture was stirred vigorously at room temperature for 85 min and the organic phase was decanted off of the amalgam residue, which was subsequently washed with ethyl acetate. The organic phases were diluted with aqueous NH_4Cl and the mixture was extracted with ethyl acetate. The combined organic phases were washed with brine, dried over $MgSO_4$ and the solvent was evaporated. The crude product was obtained as white foam (1.30 g) and used in the next step without further purification.

Sodium amalgam was prepared as described below.

An analytically pure sample was obtained by column chromatography on silica (hexane/EtOAc, 1:1 v/v).

R_f = 0.42 (hexane/EtOAc = 1/1 (*v/v*), CAM).

$[\alpha]^D_{20}$: +76.75° (c=0.29, $CHCl_3$).

^{1}H NMR (400 MHz, $CDCl_3$): δ 5.57 (s, 1H), 5.31 (s, 1H), 5.28 – 5.23 (m, 1H), 5.08 (ddd, *J* = 9.0, 7.6, 1.7 Hz, 1H), 4.79 (d, *J* = 9.5 Hz, 1H), 4.63 (d, *J* = 5.7 Hz, 1H), 3.95 (d, *J* = 9.5 Hz, 1H), 3.22 (d, *J* = 5.6 Hz, 1H), 2.68 (bs, 1H), 2.39 (dd, *J* = 15.0, 8.8 Hz, 1H), 2.35 (bs, 1H), 2.17 (dd, *J* = 14.5, 7.2 Hz, 1H), 2.13 (s, 3H), 2.05 (d, *J* = 1.5 Hz, 3H), 1.91 – 1.80 (m, 2H), 1.80 – 1.62 (m, 1H), 1.59 (s, 3H), 1.51 – 1.41 (m, 1H), 1.38 (s, 3H), 1.12 (s, 3H), 0.96 (t, *J* = 7.9 Hz, 9H), 0.63 (q, *J* = 7.9 Hz, 6H).

^{13}C NMR (101 MHz, $CDCl_3$): δ 170.3, 153.8, 144.7, 140.4, 133.3, 119.0, 91.4, 82.0, 79.0, 77.9, 72.4, 68.6, 44.4, 43.9, 41.1, 38.7, 28.0, 27.7, 25.8, 23.0, 21.9, 18.5, 15.7, 7.0 (3C), 4.9 (3C).

IR (film, cm^{-1}): 2954, 2876, 1804, 1733, 1370, 1240, 1199, 1108, 1081, 1024, 1000.

HRMS (ESI): *m/z* calcd for $C_{29}H_{46}O_8SiNa^+$ $[M+Na]^+$: 573.2854, found: 573.2855.

Large-scale preparation of sodium amalgam (2.5 wt.%):

Different methods for the preparation of sodium amalgam have been described in literature[1, 2]. The following procedure is roughly based on these preparations. It should be noted that for small scale reactions, it is usually sufficient to prepare the desired quantity of amalgam *in situ*, but in order to enable an exact dosing and reproducibility, we decided to prepare a large batch of amalgam, since it can be stored indefinitely under nitrogen atmosphere.

Pieces of freshly cleaned sodium (8.72 g) were placed in a 250 mL round-bottom flask without a stirring bar under nitrogen atmosphere. A dropping funnel with pressure equalization was attached, equipped with a nitrogen inlet and additional gas outlet on top. The funnel was filled with mercury (25.1 mL, 340 g). All connections were secured with metal clamps. A mercury vapor trap consisting of iodine-impregnated activated charcoal, filled in a one-meter-long glass tube, was attached to the gas outlet. A steady stream of nitrogen into the apparatus was used to carry evolving mercury vapors into the trap. A few drops of mercury were added to the sodium chunks, which resulted in a heavy exothermal reaction and significant splashing. The mixture first liquified, then solidified again. The remaining mercury was added dropwise, while simultaneously heating the flask with a heatgun and swirling the liquid metal around to ensure proper mixing. After complete addition, the mixture was cooled to room temperature, which resulted in solidification of the mixture. The flask was shattered with a hammer to access the solid chunk of amalgam, which was grinded into a fine powder using a mortar and pestle. The resulting gray powder is stable to air for short periods of time and can be stored indefinitely under nitrogen atmosphere.

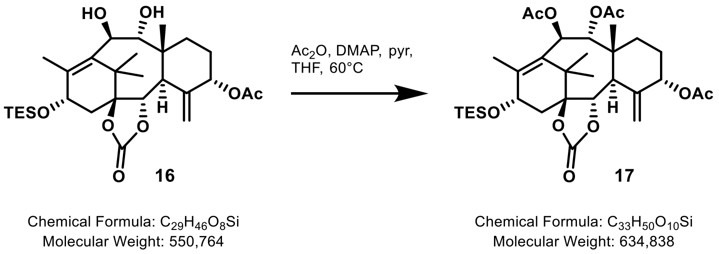

(3a*S*,5*S*,8*R*,9*R*,9a*R*,12*S*,13a*R*,13b*S*)-6,9a,14,14-tetramethyl-13-methylene-2-oxo-5-((triethylsilyl)oxy)-5,8,9,9a,10,11,12,13,13a,13b-decahydro-4*H*-3a,7-methanobenzo[3,4]-cyclodeca[1,2-*d*][1,3]dioxole-8,9,12-triyl triacetate (17)

Crude diol **16** (1.30 g, assumed 2.33 mmol, 1.00 eq.) was dissolved in dry THF (70 mL) under nitrogen atmosphere. Dry pyridine (1.88 mL, 23.3 mmol, 10.0 eq.), DMAP (142 mg, 1.17 mmol, 0.50 eq.) and acetic anhydride (2.20 mL, 23.3 mmol, 10.0 eq.) were added and the reaction mixture was heated to 60 °C. After stirring for 16 h, the mixture was cooled to room temperature and quenched with aqueous $NaHCO_3$. The phases were separated and the aqueous phase was extracted with ethyl acetate. The combined organic phases were washed with brine, dried over $MgSO_4$ and the solvent was evaporated. The crude product was purified *via* column chromatography on silica gel (hexane/EtOAc, 3:1 *v*/*v*), yielding the desired product as a white solid in (1.23 g, 1.94 mmol, 83% over two steps).

R_f = 0.54 (hexane/EtOAc = 2/1 (*v/v*), CAM).

$[\alpha]^D_{20}$: +81.62° (c=0.36, $CHCl_3$).

^{1}H NMR (400 MHz, $CDCl_3$): δ 6.05 (d, *J* = 10.6 Hz, 1H), 5.69 (d, *J* = 10.7 Hz, 1H), 5.60 (d, *J* = 1.6 Hz, 1H), 5.34 (s, 1H), 5.29 – 5.26 (m, 1H), 5.05 (t, *J* = 8.1 Hz, 1H), 4.73 (d, *J* = 5.8 Hz, 1H), 3.27 (d, *J* = 5.7 Hz, 1H), 2.41 (dd, *J* = 15.0, 8.9 Hz, 1H), 2.21 (d, *J* = 1.5 Hz, 3H), 2.17 (dd, *J* = 15.0, 8.9 Hz, 1H), 2.15 (s, 3H), 2.06 (s, 3H), 2.01 (s, 3H), 1.92 – 1.62 (m, 3H), 1.67 (s, 3H), 1.32 (s, 3H), 1.36 – 1.24 (m, 1H), 0.96 (t, *J* = 7.9 Hz, 9H), 0.95 (s, 3H), 0.63 (q, *J* = 7.9 Hz, 6H).

^{13}C NMR (101 MHz, $CDCl_3$): δ 170.2, 169.9, 153.5, 148.0, 139.9, 129.6, 119.6, 91.0, 81.7, 76.8, 72.2, 68.6, 44.5, 43.9, 40.9, 38.5, 27.7, 27.4, 27.0, 22.8, 21.9, 21.1, 20.8, 18.1, 15.6, 7.0 (3C), 4.9 (3C).

IR (film, cm^{-1}): 2954, 2876, 1807, 1739, 1370, 1226, 1201, 1080, 1021.

HRMS (ESI): *m/z* calcd for $C_{33}H_{51}O_{10}Si^+$ $[M+H]^+$: 635.3246, found: 635.3246.

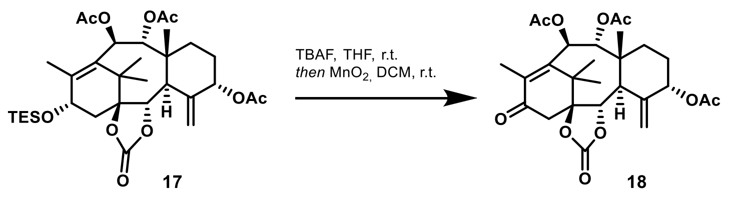

Chemical Formula: $C_{33}H_{50}O_{10}Si$
Molecular Weight: 634,838

Chemical Formula: $C_{27}H_{34}O_{10}$
Molecular Weight: 518,559

(3a*S*,8*R*,9*R*,9a*R*,12*S*,13a*R*,13b*S*)-6,9a,14,14-tetramethyl-13-methylene-2,5-dioxo-5,8,9,9a,10,11,12,13,13a,13b-decahydro-4*H*-3a,7-methanobenzo[3,4]cyclodeca[1,2-*d*][1,3]dioxole-8,9,12-triyl triacetate (18)

Silyl ether **17** (1.30 g, 2.05 mmol, 1.00 eq.) was dissolved in dry THF (128 mL), TBAF (1M in THF, 2.25 mL, 2.25 mmol, 1.10 eq.) was added and the reaction was stirred for 10 min at room temperature. The reaction mixture was quenched with aqueous NH_4Cl and extracted with ethyl acetate. The combined organic phases were washed with brine and dried over $MgSO_4$. After evaporation of the solvents, the residue was dissolved in dry dichloromethane (128 mL) and activated manganese dioxide (1.78 g, 20.5 mmol, 10.0 eq.) was added. After 6 h, additional manganese dioxide (1.78 g, 20.5 mmol, 10.0 eq.) was added. After further stirring for 15 h, the mixture was filtered over celite and the solvent was removed under reduced pressure. The residue was subjected to column chromatography on silica gel (hexane/EtOAc, 2:1 *v*/*v*) to yield the desired product as a white foam (1.02 g, 1.97 mmol, 96%).

R_f = 0.61 (hexane/EtOAc = 1/1 (*v/v*), CAM).

$[\alpha]^D_{20}$: +188.93° (c=0.43, $CHCl_3$).

^{1}H NMR (400 MHz, $CDCl_3$): δ 6.05 (d, *J* = 10.3 Hz, 1H), 5.73 (d, *J* = 10.3 Hz, 1H), 5.55 (s, 1H), 5.35 (s, 1H), 5.28 – 5.24 (m, 1H), 4.91 (d, *J* = 5.8 Hz, 1H), 3.24 (d, *J* = 5.7 Hz, 1H), 2.92 (d, *J* = 19.5 Hz, 1H), 2.83 (d, *J* = 19.3 Hz, 1H), 2.27 (s, 3H), 2.10 (s, 3H), 2.06 (s, 3H), 1.94 (s, 3H), 1.90 – 1.55 (m, 3H), 1.68 (s, 3H), 1.32 (s, 3H), 1.36 – 1.23 (m, 1H), 0.98 (s, 3H).

^{13}C NMR (101 MHz, $CDCl_3$): δ 196.3, 170.1, 169.8, 169.7, 152.4, 150.1, 142.2, 139.5, 119.3, 88.8, 79.4, 77.0, 75.5, 72.9, 44.8, 43.2, 41.2 (2C), 32.9, 27.7, 27.3, 21.4, 20.9, 20.8, 20.3, 17.5, 14.5.

IR (film, cm^{-1}): 1815, 1741, 1686, 1372, 1268, 1224, 1201, 1111, 1028.

HRMS (ESI): *m/z* calcd for $C_{27}H_{35}O_{10}^+$ [*M*+H]$^+$: 519.2225, found: 519.2225.

Notes:

R_f = 0.38 (hexane/EtOAc = 1/1 (v/v), CAM). *(C13-OH intermediate)*

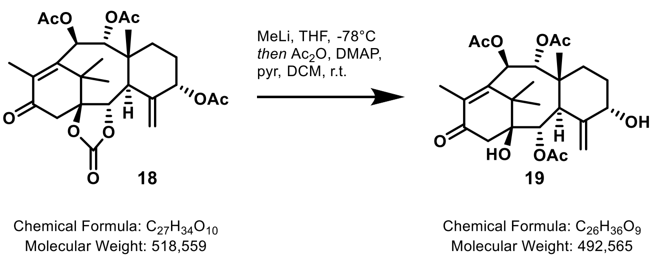

(3*S*,4a*R*,5*S*,6*S*,11*R*,12*R*,12a*R*)-3,6-dihydroxy-9,12a,13,13-tetramethyl-4-methylene-8-oxo-1,2,3,4,4a,5,6,7,8,11,12,12a-dodecahydro-6,10-methanobenzo[10]annulene-5,11,12-triyl triacetate (19)

Carbonate **18** (795 mg, 1.53 mmol, 1.00 eq.) was dissolved in dry THF (80 mL) and cooled to –78 °C. MeLi (1.6M in Et_2O, 19.1 mL, 30.6 mmol, 20.0 eq.) was added dropwise. After 3 h, the reaction was quenched with aqueous NH_4Cl at –78 °C and subsequently warmed to room temperature. The aqueous phase was extracted with ethyl acetate, the combined organic phases were washed with brine, dried over $MgSO_4$ and the solvent was removed *in vacuo*. The residue was dissolved in dry DCM (80 mL), followed by addition of dry pyridine (0.62 mL, 7.65 mmol, 5.00 eq.), DMAP (187 mg, 1.53 mmol, 1.00 eq.) and acetic anhydride (0.47 mL, 4.97 mmol, 3.25 eq.). The reaction was quenched after 30 min with aqueous $NaHCO_3$, extracted with DCM and the combined organic phases were washed with brine, dried over $MgSO_4$ and the solvent was removed in vacuo. Column chromatography on silica gel (hexane/EtOAc, 2:1 *v*/*v*) gave 408 mg of the desired product and 180 mg of more polar compounds (presumably a mixture of regioisomeric diacetates), which were resubmitted to acetylation conditions at room temperature in 20 mL of dry DCM with dry pyridine (0.14 mL), DMAP (21.4 mg) and acetic anhydride (0.07 mL). After 3 h, workup as before gave additional 47.0 mg of product. In total, 455 mg of the desired product were obtained as an impure white solid (0.92 mmol, 60%).

R_f = 0.40 (EtOAc/hexane = 2/1 (*v/v*), CAM).

$[\alpha]^D_{20}$: +144.95° (c=0.64, $CHCl_3$).

^{1}H NMR (400 MHz, $CDCl_3$): δ 6.15 (d, *J* = 10.4 Hz, 1H), 5.89 (d, *J* = 10.3 Hz, 1H), 5.60 (d, *J* = 6.5 Hz, 1H), 5.15 (s, 1H), 4.64 (s, 1H), 4.17 (s, 1H), 3.73 (d, *J* = 6.4 Hz, 1H), 2.81 (d, *J* = 19.6 Hz, 1H), 2.59 (d, *J* = 19.5 Hz, 1H), 2.22 (s, 3H), 2.19 – 2.13 (m, 1H), 2.11 (s, 3H), 2.07 (s, 3H), 2.05 (s, 3H), 1.94 – 1.53 (m, 4H), 1.68 (s, 3H), 1.20 (s, 3H), 0.89 (s, 3H).

^{13}C NMR (101 MHz, $CDCl_3$): δ 199.8, 171.9, 170.2, 169.8, 151.6, 147.3, 140.7, 114.0, 78.8, 76.2, 75.8, 73.1, 72.5, 45.1, 44.0, 43.8, 43.0, 34.4, 30.8, 26.8, 21.3, 21.0, 20.8, 20.0, 17.4, 14.0.

IR (film, cm^{-1}): 3457, 2936, 2854, 1737, 1665, 1371, 1224, 1058, 1017.

HRMS (ESI): *m/z* calcd for $C_{26}H_{35}O_8^+$ $[M\text{-}H_2O+H]^+$: 475.2326, found: 475.2325.

Notes:

*Compound **19** contains a minor inseparable impurity, which can only be removed after the next step (photocyclization towards compound 20). This impurity is presumably the regioisomeric triacetate **S7**, as indicated by the presence of regioisomeric cyclotaxane **S6** after the next step (vide infra).*

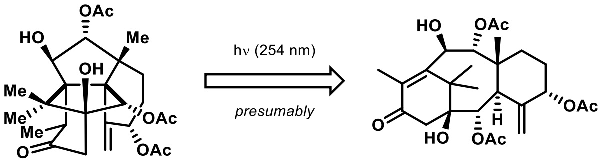

S6 **S7**

*All attempts towards cleavage of C1-C2 carbonate via hydrolysis or treatment with K_2CO_3/MeOH failed, therefore a nucleophilic approach with MeLi was employed. Due to concomitant partial cleavage of various acetates, a regioisomeric mixture of polyols (**R_f** = 0.00-0.30 (EtOAc/hexane = 2/1 (v/v)) was obtained. By leveraging the relatively low nucleophilicity of the C5 hydroxyl group, this mixture can be converted to the desired product by careful reacetylation. Occasionally, partial overacetylation towards tetraacetate **S8** is observed. However, **S8** can be recycled by employing the same deacetylation/reacetylation sequence as described for carbonate **18**.*

Multiple cycles of carbonate cleavage and reacetylation/deacetylation of the corresponding side products were performed, delivering the desired product on multigram-scale.

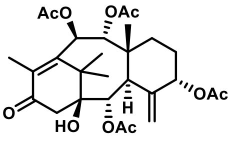

S8

Chemical Formula: $C_{28}H_{38}O_{10}$
Molecular Weight: 534,602

(3S,4aR,5S,6S,11R,12R,12aR)-6-hydroxy-9,12a,13,13-tetramethyl-4-methylene-8-oxo-1,2,3,4,4a,5,6,7,8,11,12,12a-dodecahydro-6,10-methanobenzo[10]annulene-3,5,11,12-tetrayl tetraacetate (S8)

R_f *= 0.60 (EtOAc/hexane = 2/1 (v/v), CAM).z*

^{1}H NMR *(400 MHz, $CDCl_3$): δ 6.11 (d, J = 10.4 Hz, 1H), 5.93 (d, J = 10.4 Hz, 1H), 5.60 (d, J = 6.8 Hz, 1H), 5.34 (s, 1H), 5.24 (s, 1H), 4.71 (s, 1H), 3.38 (d, J = 6.8 Hz, 1H), 2.78 (d, J = 19.7 Hz, 1H), 2.63 (d, J = 19.8 Hz, 1H), 2.25 (s, 3H), 2.12 (s, 3H), 2.09 (s, 3H), 2.07 (s, 3H), 1.99 (s, 3H), 1.86 – 1.61 (m, 4H), 1.69 (s, 3H), 1.22 (s, 3H), 0.93 (s, 3H).*

^{13}C NMR *(101 MHz, $CDCl_3$): δ 199.0, 172.0, 170.3, 170.1, 169.8, 152.0, 142.1, 140.0, 117.4, 78.9, 78.0, 75.5, 73.1, 72.1, 46.0, 44.9, 43.8, 42.9, 34.5, 28.7, 27.7, 21.5, 21.3, 21.0, 20.9, 20.0, 17.6, 13.9.*

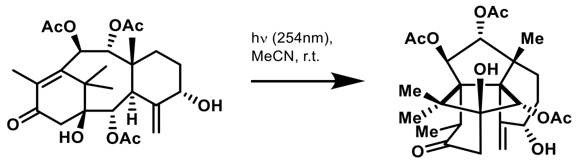

19

Chemical Formula: $C_{26}H_{36}O_9$
Molecular Weight: 492,565

20

Chemical Formula: $C_{26}H_{36}O_9$
Molecular Weight: 492,565

(2*S*,4a*R*,5*R*,6*R*,6a*S*,10*S*,11a*R*)-2,10-dihydroxy-4a,7,12,12-tetramethyl-1-methylene-8-oxodecahydro-1*H*,7*H*-6a,10-methanobenzo[c]azulene-5,6,11-triyl triacetate (20)

Impure cyclization precursor **19** (1.45 g, 2.94 mmol, 1.00 eq.) was dissolved in degassed acetonitrile (450 mL) in a quartz flask under nitrogen atmosphere. The reaction mixture was irradiated under vigorous stirring at 254 nm for 6x3 min (8x OSRAM PURITEC HNS L 18W, 5.5 W emission around 254 nm, irradiation from the outside from a distance of around 8 cm) with a 5 min cooldown period between each cycle. The solvent was evaporated and the residue was subjected to column chromatography on silica gel (EtOAc/hexane, 2:1 *v*/*v*) to yield the desired product as a white solid (1.04 g, 2.10 mmol, 72%) accompanied by regioisomeric compound **S6** (73.7 mg, 0.15 mmol, 5%, cf. notes).

R_f = 0.34 (EtOAc/hexane = 3/1 (*v/v*), CAM).

$[\alpha]^D_{20}$: +4.36° (c=0.56, $CHCl_3$).

^{1}H NMR (400 MHz, $CDCl_3$): δ 6.09 (s, 1H), 5.77 (s, 1H), 5.70 (d, *J* = 9.7 Hz, 1H), 5.67 (d, *J* = 9.7 Hz, 1H), 5.56 (s, 1H), 4.42 (t, *J* = 8.9 Hz, 1H), 3.78 (q, *J* = 7.2, 1H), 2.88 (d, *J* = 20.2 Hz, 1H), 2.71 – 2.55 (m, 1H), 2.38 (d, *J* = 20.3 Hz, 1H), 2.12 (s, 3H), 2.04 (s, 3H), 2.03 (s, 3H), 2.02 – 1.94 (m, 1H), 1.79 – 1.66 (m, 2H), 1.60 (s, 3H), 1.23 (s, 3H), 1.20 (d, *J* = 7.1 Hz, 3H), 1.07 (s, 3H), 1.09 – 1.00 (m, 1H).

^{13}C NMR (101 MHz, $CDCl_3$): δ 214.8, 172.0, 171.2, 170.0, 146.1, 126.6, 82.6, 79.9, 79.5, 79.2, 75.0, 61.8, 56.2, 52.1, 46.4, 45.4, 45.1, 31.4, 28.0, 26.5, 23.4, 22.5, 21.5, 21.2, 21.1, 15.8.

IR (film, cm^{-1}): 3466, 2921, 2850, 1740, 1438, 1368, 1229, 1102, 1046.

HRMS (ESI): *m/z* calcd for $C_{26}H_{35}O_8^+$ [*M*-H_2O+H]$^+$: 475.2326, found: 475.2326.

Notes:

*The formation of regioisomeric cyclotaxane triacetate **S6** is most likely originating from the uncharacterized, inseparable impurity in the cyclization precursor **19**, which therefore might be the corresponding regioisomeric classical taxane triacetate **S7**.*

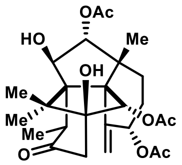

S6

Chemical Formula: $C_{26}H_{36}O_9$
Molecular Weight: 492,565

(2S,4aR,5R,6R,6aS,10S,11aR)-5,10-dihydroxy-4a,7,12,12-tetramethyl-1-methylene-8-oxodecahydro-1H,7H-6a,10-methanobenzo[c]azulene-2,6,11-triyl triacetate (S6)

R_f *= 0.40 (EtOAc/PE = 3/1 (v/v), CAM).*

^{1}H NMR *(400 MHz, $CDCl_3$): δ = 6.03 (d, J = 2.0 Hz, 1H), 5.77 (s, 1H), 5.57 (s, 1H), 5.49 (d, J = 8.2 Hz, 1H), 5.44 (d, J = 13.0 Hz, 1H), 4.36 (d, J = 9.6 Hz, 1H), 3.32 (d, J = 7.2 Hz, 1H), 3.07 (d, J = 3.1 Hz, 1H), 2.80 (d, J = 20.5 Hz, 1H), 2.64 (bs, 1H), 2.34 (d, J = 20.3 Hz, 1H), 2.11 (s, 3H), 2.08 (s, 3H), 2.02 (s, 3H), 2.03 – 1.98 (m, 1H), 1.66 – 1.57 (m, 1H), 1.52 (s, 3H), 1.50 – 1.45 (m, 1H), 1.42 (d, J = 7.1 Hz, 3H), 1.27 (s, 3H), 1.07 (s, 3H), 1.08 – 1.00 (m, 1H).*

^{13}C NMR *(101 MHz, $CDCl_3$): δ = 214.4, 172.4, 171.6, 169.7, 141.5, 129.3, 86.7, 80.6, 79.5, 79.2, 76.3, 62.0, 57.6, 52.0, 46.3, 45.3, 44.8, 31.3, 27.2, 25.7, 23.3, 22.2, 21.5, 21.5, 21.2, 16.3.*

Experimental setup for step 16:

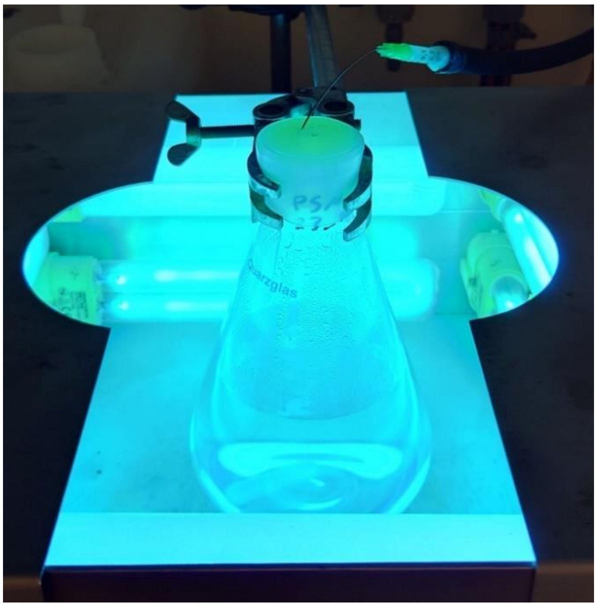

Figure S1: Experimental setup for the large-scale photocyclization.

- *8x OSRAM PURITEC HNS L 18W, 5.5 W emission around 254 nm,*
- *irradiation from the outside from a distance of around 8 cm*

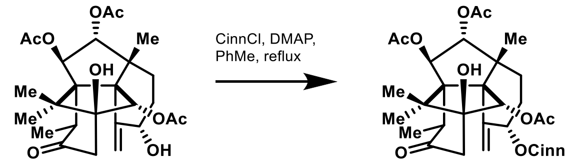

20 — Chemical Formula: $C_{26}H_{36}O_9$; Molecular Weight: 492,565

1-hydroxytaxuspine C (1) — Chemical Formula: $C_{26}H_{35}O_8$; Molecular Weight: 475,558

1-Hydroxytaxuspine C (1)

Allylic alcohol **20** (6.00 mg, 12.2 µmol, 1.00 eq.) was dissolved in dry toluene (1 mL) under nitrogen atmosphere. DMAP (17.9 mg, 0.15 mmol, 12.0 eq.) and *trans*-cinnamoyl chloride (20.3 mg, 0.12 mmol, 10.0 eq.) were added and the mixture was heated to reflux for 1 h. The reaction was cooled to room temperature, quenched with aqueous $NaHCO_3$ and extracted with ethyl acetate. The combined organic phases were washed with brine, dried over $MgSO_4$ and the solvent was evaporated. Purification *via* column chromatography on silica gel (EtOAc/hexane, 2:1 *v/v*) gave the desired product in 89% yield (6.72 mg, 10.8 µmol).

R_f = 0.47 (EtOAc/hexane = 2/1 (*v/v*), CAM).

$[\alpha]^D_{20}$: +3.65° (c=0.76, $CHCl_3$).

^{1}H NMR (400 MHz, $CDCl_3$): δ 7.68 (d, *J* = 16.0 Hz, 1H), 7.59 – 7.53 (m, 2H), 7.41 – 7.35 (m, 3H), 6.39 (d, *J* = 16.0 Hz, 1H), 6.14 (d, *J* = 2.0 Hz, 1H), 5.88 (s, 1H), 5.75 (s, 1H), 5.74 (s, 1H), 5.72 (s, 1H), 5.64 (t, *J* = 8.7 Hz, 1H), 3.49 (q, *J* = 7.2 Hz, 1H), 2.88 (d, *J* = 20.3 Hz, 1H), 2.42 (d, *J* = 20.3 Hz, 1H), 2.25 – 2.16 (m, 1H), 2.15 (s, 3H), 2.06 (s, 3H), 2.06 (s, 3H), 1.90 – 1.71 (m, 2H), 1.63 (s, 3H), 1.29 (d, *J* = 7.0 Hz, 3H), 1.28 (s, 3H), 1.16 – 1.12 (m, 1H), 1.10 (s, 3H).

^{13}C NMR (101 MHz, $CDCl_3$): δ 213.1, 172.1, 171.1, 170.1, 165.8, 145.7, 141.1, 134.4, 130.6, 129.9, 129.1 (2C), 128.4 (2C), 117.9, 82.4, 79.8, 79.4, 79.3, 76.5, 61.6, 56.3, 51.7, 46.4, 45.4, 45.3, 31.3, 26.6, 25.9, 23.5, 22.7, 21.5, 21.3, 21.1, 15.9.

IR (film, cm^{-1}): 2926, 2855, 1745, 1706, 1635, 1449, 1366, 1224, 1154, 1109, 1047.

HRMS (ESI): *m/z* calcd for $C_{35}H_{42}O_{10}Na^+$ $[M+Na]^+$: 645.2670, found: 645.2668.

3. Comparison of Natural and Synthetic 1-Hydroxytaxuspine C NMR Data

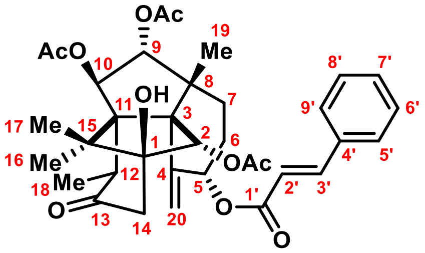

*Table S1: ¹H-NMR Chemical shifts comparison of isolated and synthetic 1-hydroxytaxuspine C (**1**).*

Position	Synthetic (this work) (400 MHz, $CDCl_3$)	Shi *et al.*[3] (300 MHz, $CDCl_3$)	Kosugi *et al.*[4] (500 MHz, $CDCl_3$)
**1**	–	–	–
**2**	6.14 d (*J* = 2.0 Hz)	6.14 bs	6.14 d (*J* = 2.0 Hz)
**3**	–	–	–
**4**	–	–	–
**5**	5.64 t (*J* = 8.7 Hz)	5.65 bt (*J* = 8.8 Hz)	5.64 bt (*J* = 9.0 Hz)
**6α**	1.90-1.70 m	1.80 m	1.82 m
**6β**	2.25-2.16 m	2.18 m	2.20 m
**7α**	1.90-1.70 m	1.85 m	1.78 m
**7β**	1.16-1.12 m	1.12 m	1.16 m
**8**	–	–	–
**9**	5.74 s	5.75 s	5.76 d (*J* = 10.0 Hz)
**10**	5.75 s	5.75 s	5.74 d (*J* = 10.0 Hz)
**11**	–	–	–
**12**	3.49 q (*J* = 7.2 Hz)	3.50 q (*J* = 7.1 Hz)	3.35 q (*J* = 7.1 Hz) (*)
**13**	–	–	–
**14α**	2.88 d (*J* = 20.3 Hz)	2.89 d (*J* = 20.3 Hz)	2.89 d (*J* = 20.5 Hz)
**14β**	2.42 d (*J* = 20.3 Hz)	2.43 d (*J* = 20.3 Hz)	2.43 d (*J* = 20.5 Hz)
**15**	–	–	–

**16**	1.10 s	1.10 s	1.11 s
**17**	1.63 s	1.64 s	1.64 s
**18**	1.29 d (*J* = 7.0 Hz)	1.30 d (*J* = 7.2 Hz)	1.30 d (*J* = 7.1 Hz)
**19**	1.28 s	1.29 s	1.29 s
**20α**	5.72 s	5.72 s	5.72 d (*J* = 0.7 Hz)
**20β**	5.88 s	5.89 s	5.88 s
**1-OH**	–	–	2.54 s
**1'**	–	–	–
**2'**	6.39 d (*J* = 16.0 Hz)	6.40 d (*J* = 15.9 Hz)	6.39 d (*J* = 16.1 Hz)
**3'**	7.68 d (*J* = 16.0 Hz)	7.69 d (*J* = 16.0 Hz)	7.68 d (*J* = 16.1 Hz)
**4'**	–	–	–
**5', 9'**	7.59-7.53 m	7.56 m	7.57 m
**6', 8'**	7.41-7.35 m	7.40 m	7.39 m
**7'**	7.41-7.35 m	7.40 m	7.39 m
**2-OAc**	2.15 s	2.17 s	2.16 s, 2.07 s, 2.06 s
**9-OAc**	2.06 s	2.06 s	2.16 s, 2.07 s, 2.06 s
**10-OAc**	2.06 s	2.16 s (*)	2.16 s, 2.07 s, 2.06 s

(*) Note: The spectral data of the synthetic material is largely in accordance with the reported data, besides from the signals for C10-OAc (2.16 vs. 2.06 ppm in [3]) and C12-H (3.49 vs. 3.35 ppm in [4]). However, both discrepancies are in turn congruent with the other literature reference, respectively. Unfortunately, no original spectra are available, thereby no reevaluation of the literature was possible.

The synthetic material was fully characterized *via* HSQC, HMBC and NOESY experiments.

*Table S2: ^{13}C-NMR Chemical shifts comparison of isolated and synthetic 1-hydroxytaxuspine C (**1**).*

Position	Synthetic (this work) (101 MHz, $CDCl_3$)	Shi *et al.*[3] (125 MHz, $CDCl_3$)	Kosugi *et al.*[4] (125 MHz, $CDCl_3$)
**1**	79.27	79.08	79.12
**2**	79.37	79.12	79.19
**3**	64.64	61.38	61.45
**4**	141.13	140.86	140.92
**5**	76.48	76.29	76.31
**6**	26.55	26.41	25.77
**7**	31.26	31.07	31.10
**8**	45.36	45.14	45.20
**9**	82.37	79.55 (*)	82.20
**10**	79.75	79.17	79.59
**11**	56.31	56.04	56.12
**12**	51.74	51.55	51.58
**13**	213.05	212.96	212.89
**14**	46.40	46.18	46.23
**15**	45.27	45.08	45.11
**16**	22.69	22.51	22.53
**17**	23.45	23.30	23.30
**18**	15.94	15.77	15.79
**19**	25.93	25.73	26.39
**20**	129.86	130.44	129.75
**1’**	165.82	165.65	165.66
**2’**	117.86	117.61	117.68
**3’**	145.66	145.48	145.50

**4’**	134.43	134.18	134.24
**5’, 9’**	128.40	128.25	128.24
**6’, 8’**	129.05	128.87	128.90
**7’**	130.59	130.44	130.44
**2-OAc**	172.09	171.98	171.99
**9-OAc**	171.08	170.94	169.96 (*)
**10-OAc**	170.12	169.97	170.93 (*)
	21.53, 21.26, 21.07	21.42, 21.13, 20.95	21.94, 21.41, 21.12

Note: The spectral data of the synthetic material is in accordance with the reported data, besides from the signals for C9 (82.37 vs. 79.55 ppm in [3]) and C9/C10-OAc (171.08/170.12 vs. 169.96/170.93 ppm in [4]). The acetates were assigned to C10 and C9 *via* HMBC experiments. However, both discrepancies are in turn congruent with the other literature reference, respectively. Unfortunately, no original spectra are available, thereby no reevaluation of the literature was possible.

The synthetic material was fully characterized *via* HSQC, HMBC and NOESY experiments.

4. References

[1] A. F. Holleman, *Org. Synth.* **1927**, *7*, 88.

[2] W. B. R. Jr, C. R. Hauser, *Org. Synth.* **1939**, *19*, 83.

[3] Q.-W. Shi, T. Oritani, T. Horiguchi, T. Sugiyama, R. Murakami, T. Yamada, *Bioscience, Biotechnology, and Biochemistry* **1999**, *63*, 924-929.

[4] K. Kosugi, J.-i. Sakai, S. Zhang, Y. Watanabe, H. Sasaki, T. Suzuki, H. Hagiwara, N. Hirata, K. Hirose, M. Ando, A. Tomida, T. Tsuruo, *Phytochemistry* **2000**, *54*, 839-845.

5. NMR Spectra

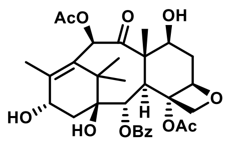

6

^{1}H NMR (400 MHz, $CDCl_3$)

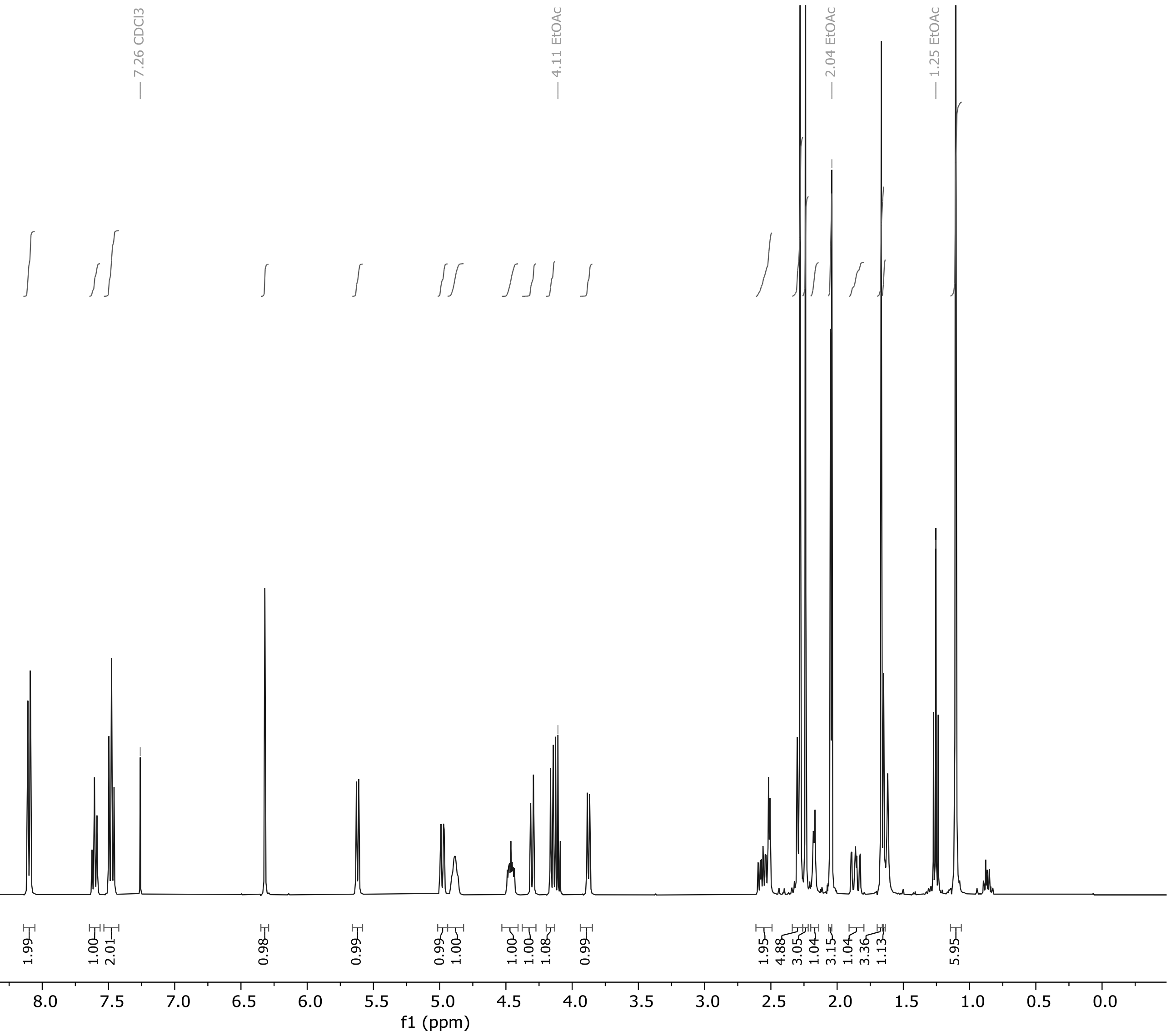

^{13}C NMR (101 MHz, $CDCl_3$)

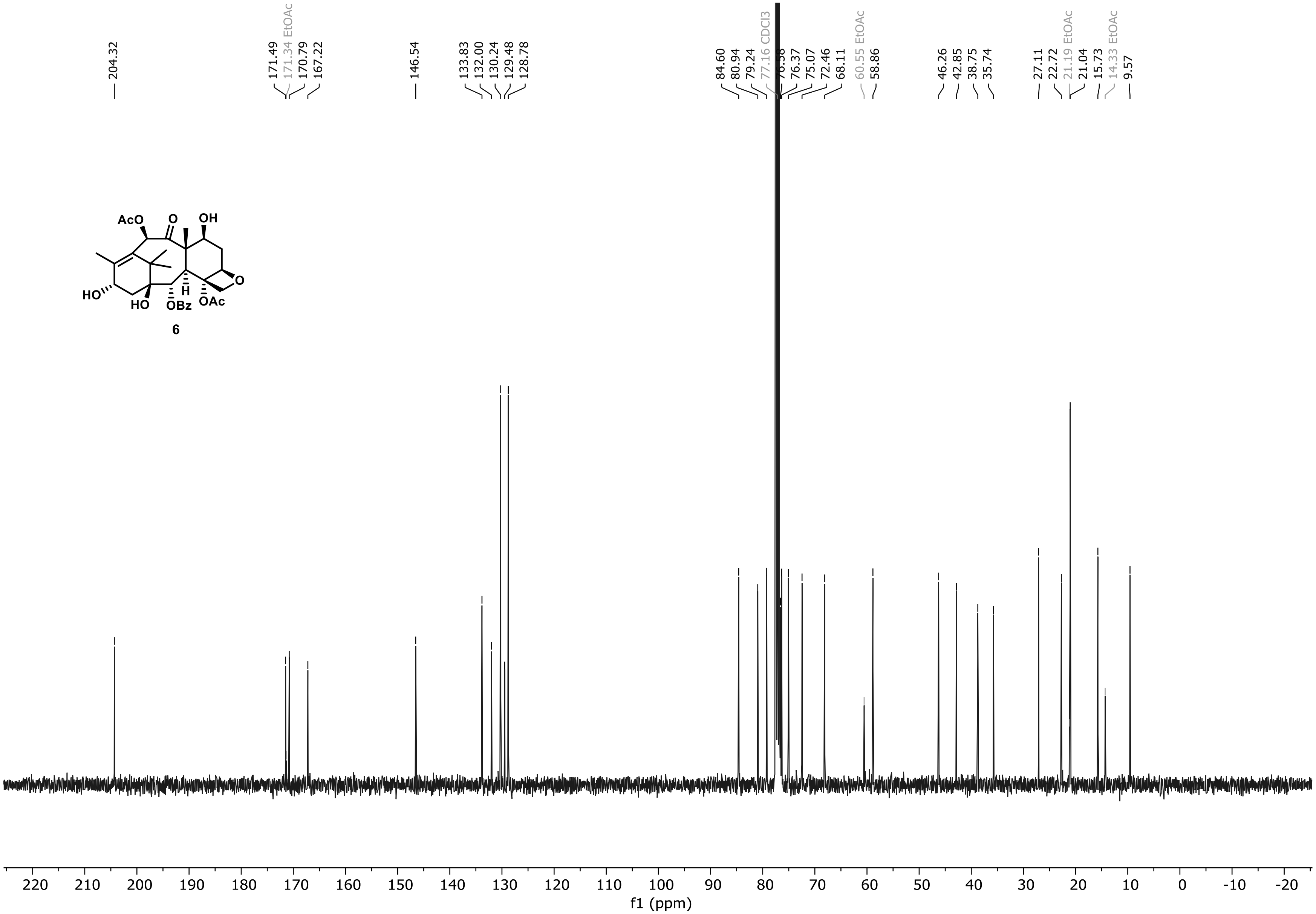

^{1}H NMR (400 MHz, $CDCl_3$)

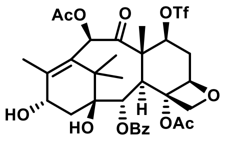

7

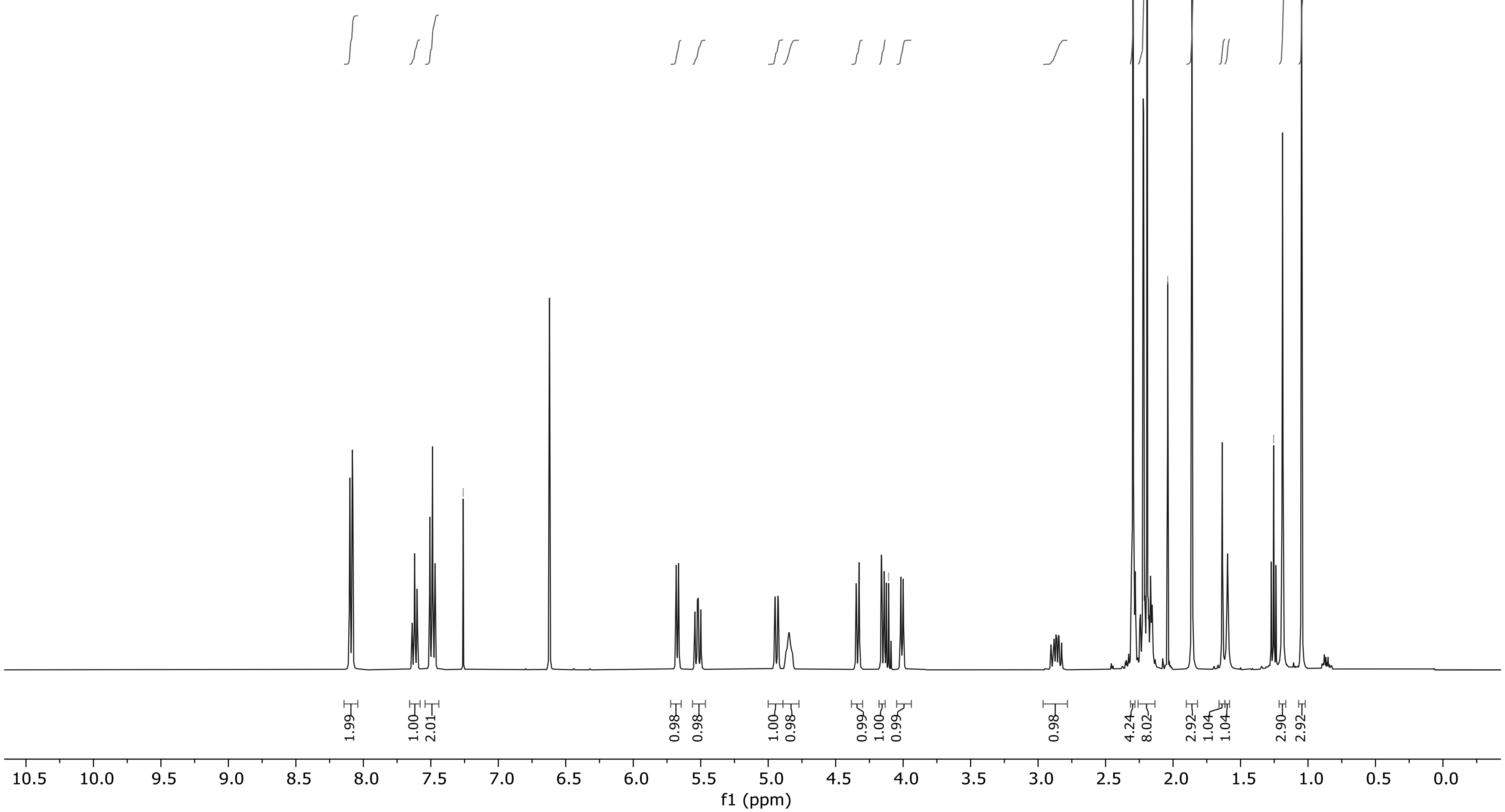

^{13}C NMR (101 MHz, CDCl$_3$)

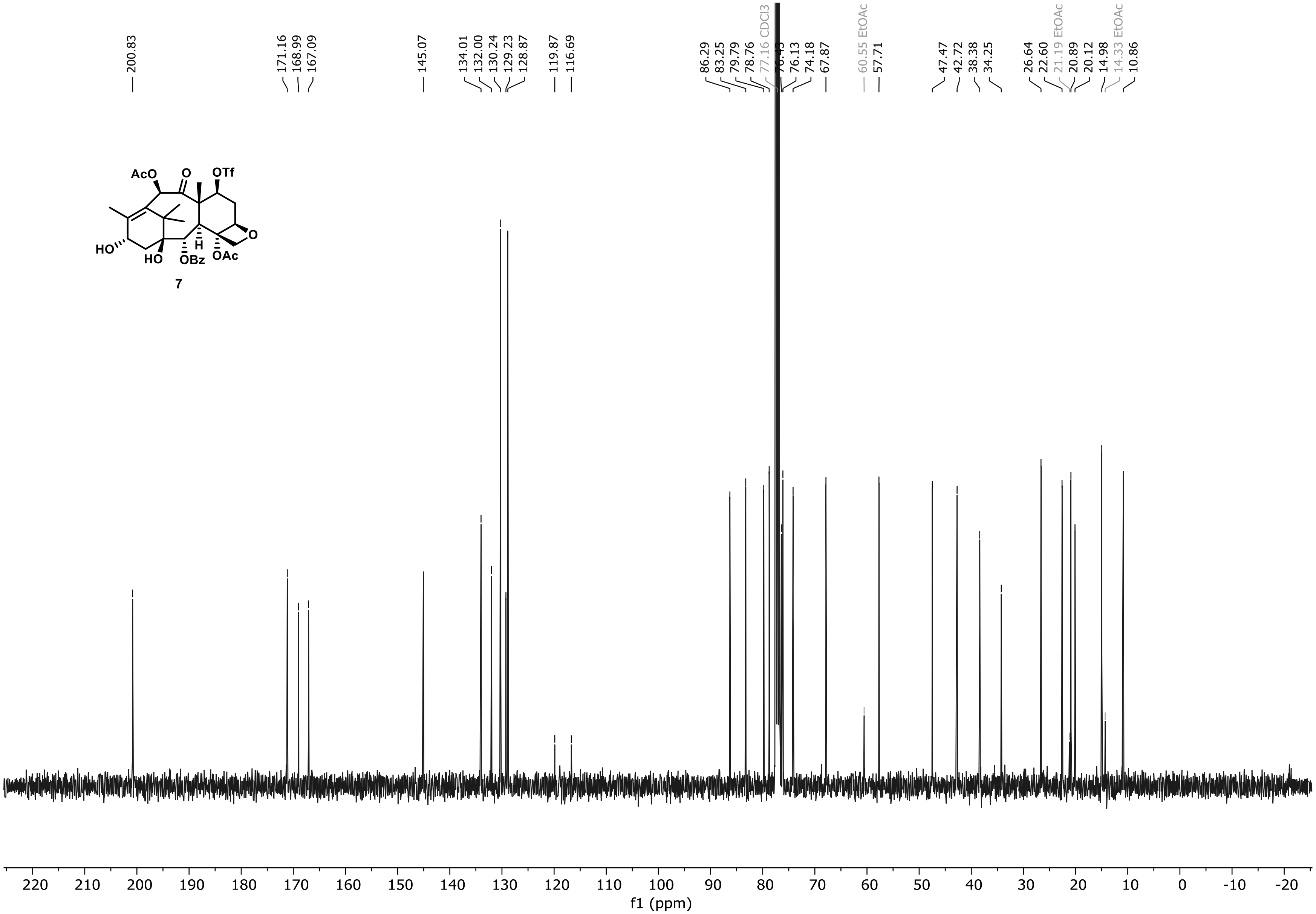

^{1}H NMR (400 MHz, $CDCl_3$)

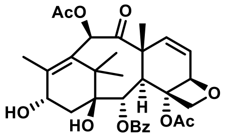

8

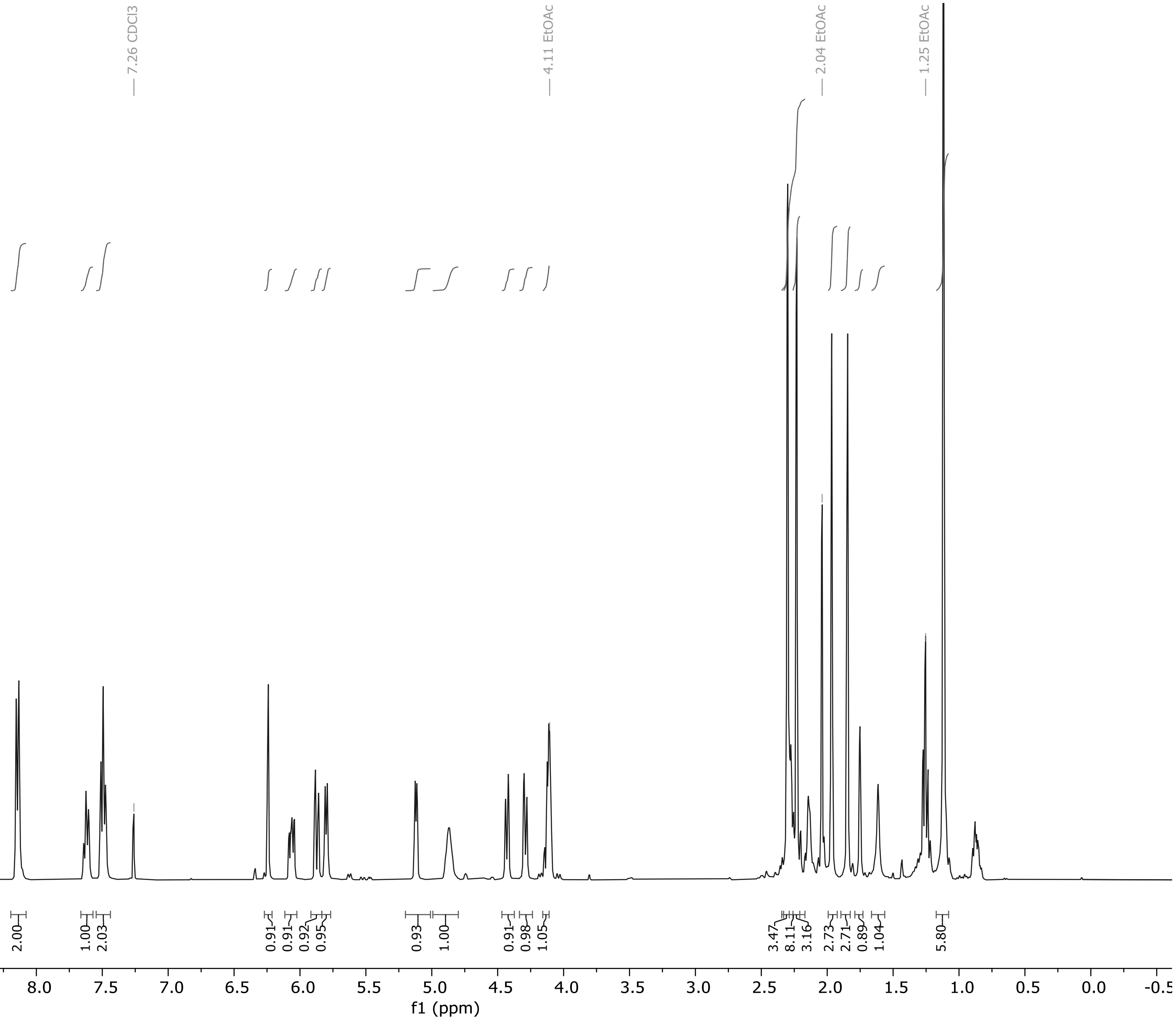

^{13}C NMR (101 MHz, CDCl$_3$)

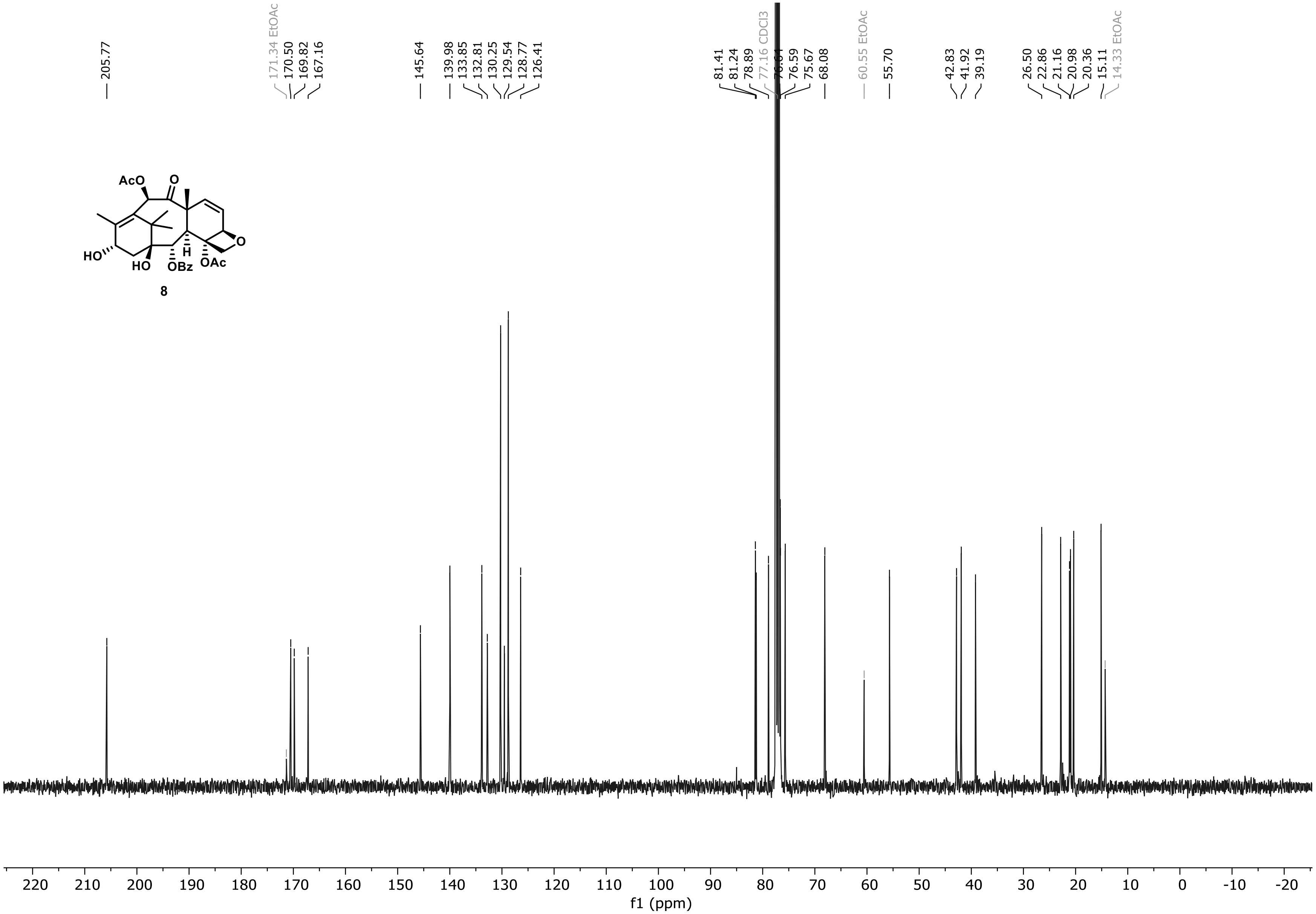

^{1}H NMR (400 MHz, $CDCl_3$)

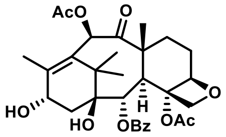

9

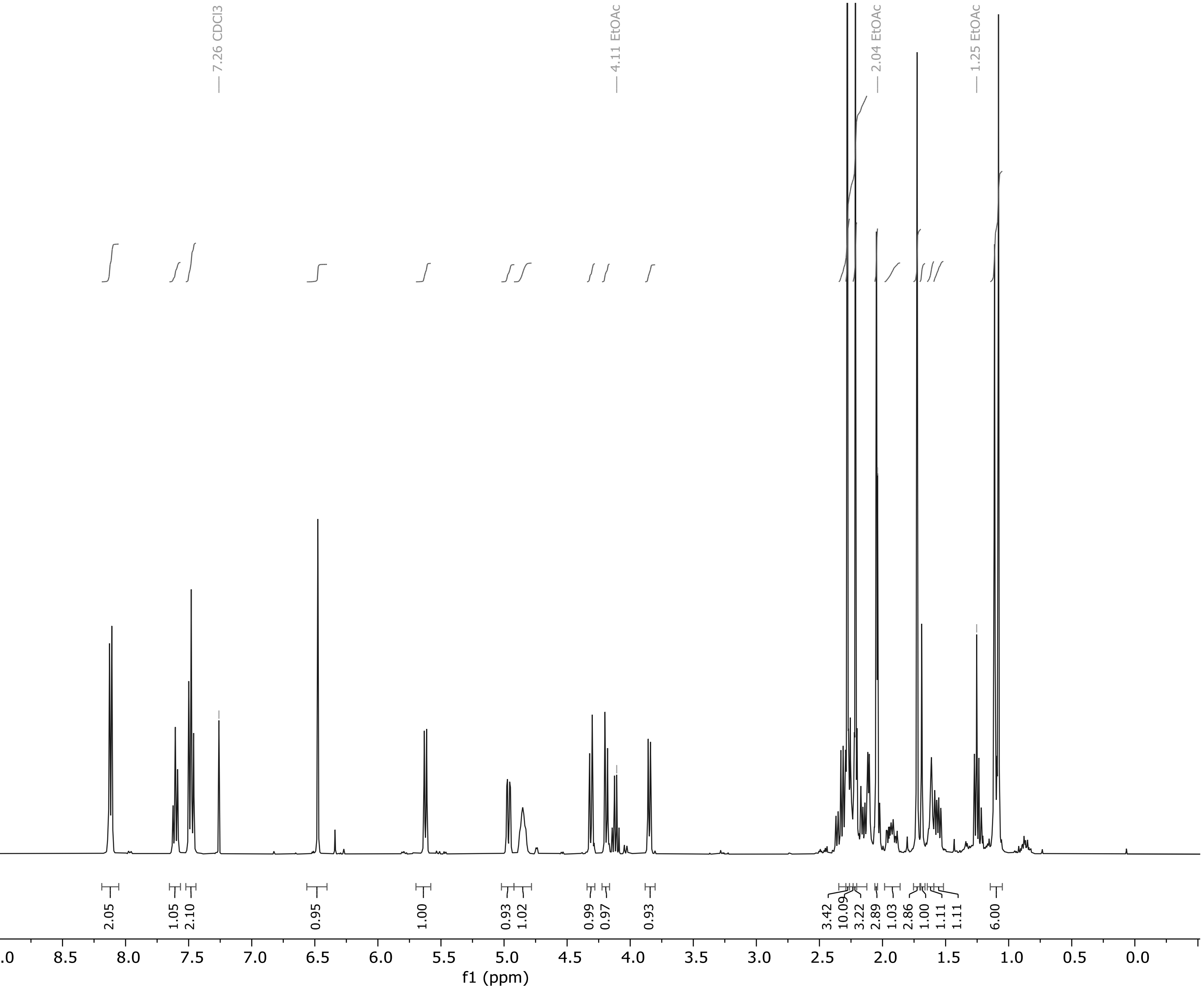

^{13}C NMR (101 MHz, $CDCl_3$)

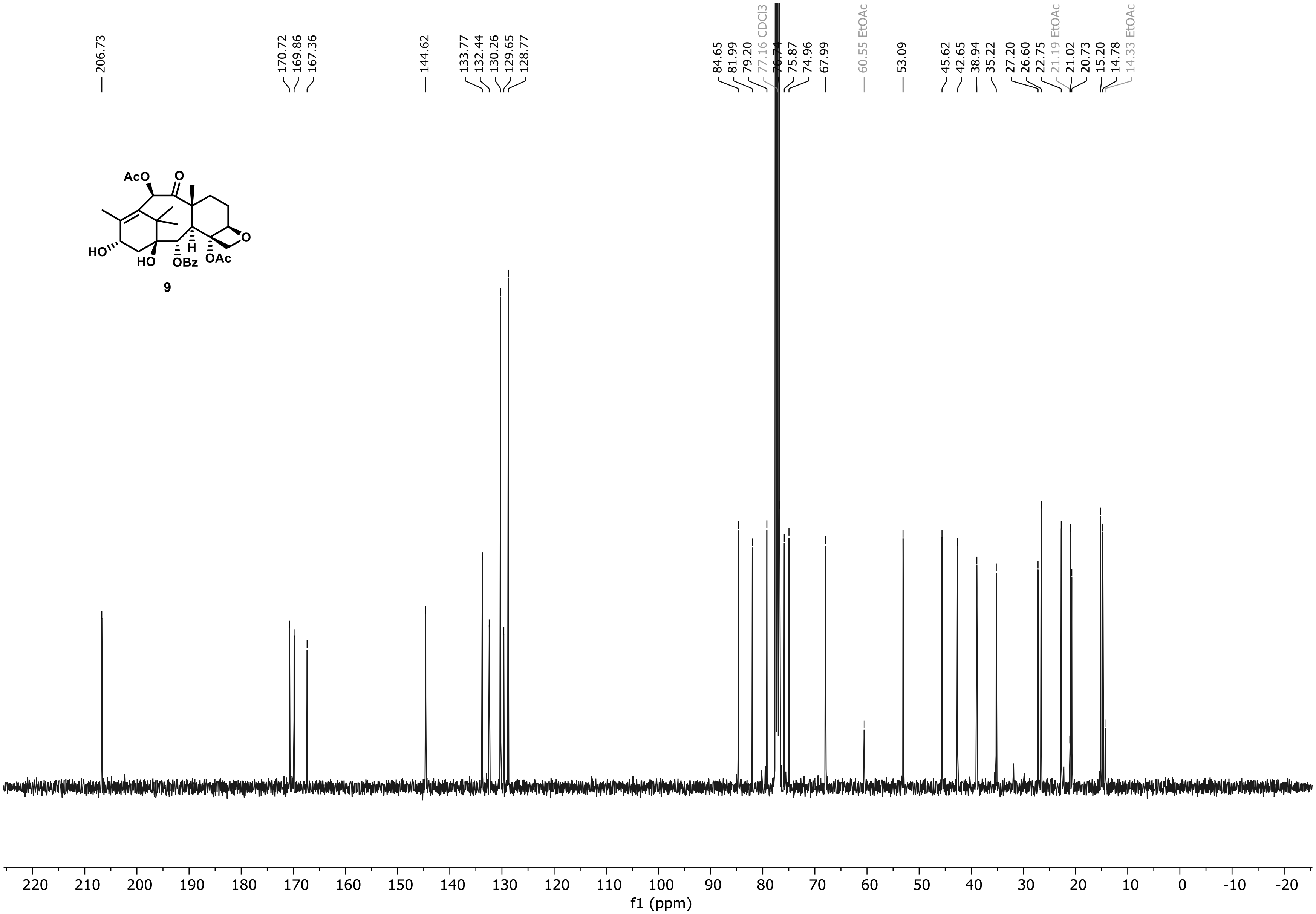

^{1}H NMR (400 MHz, $CDCl_3$)

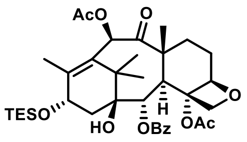

10

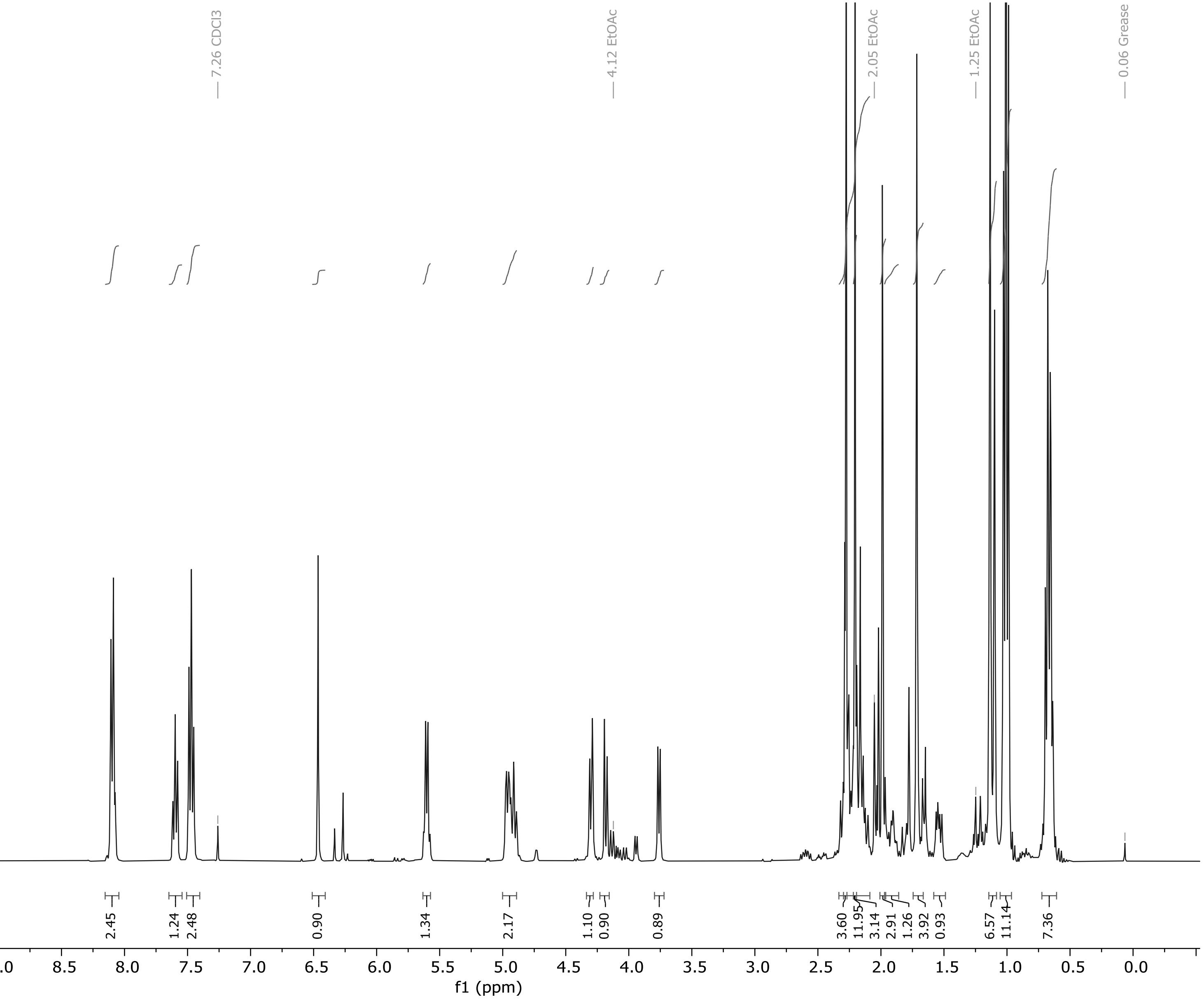

^{13}C NMR (101 MHz, $CDCl_3$)

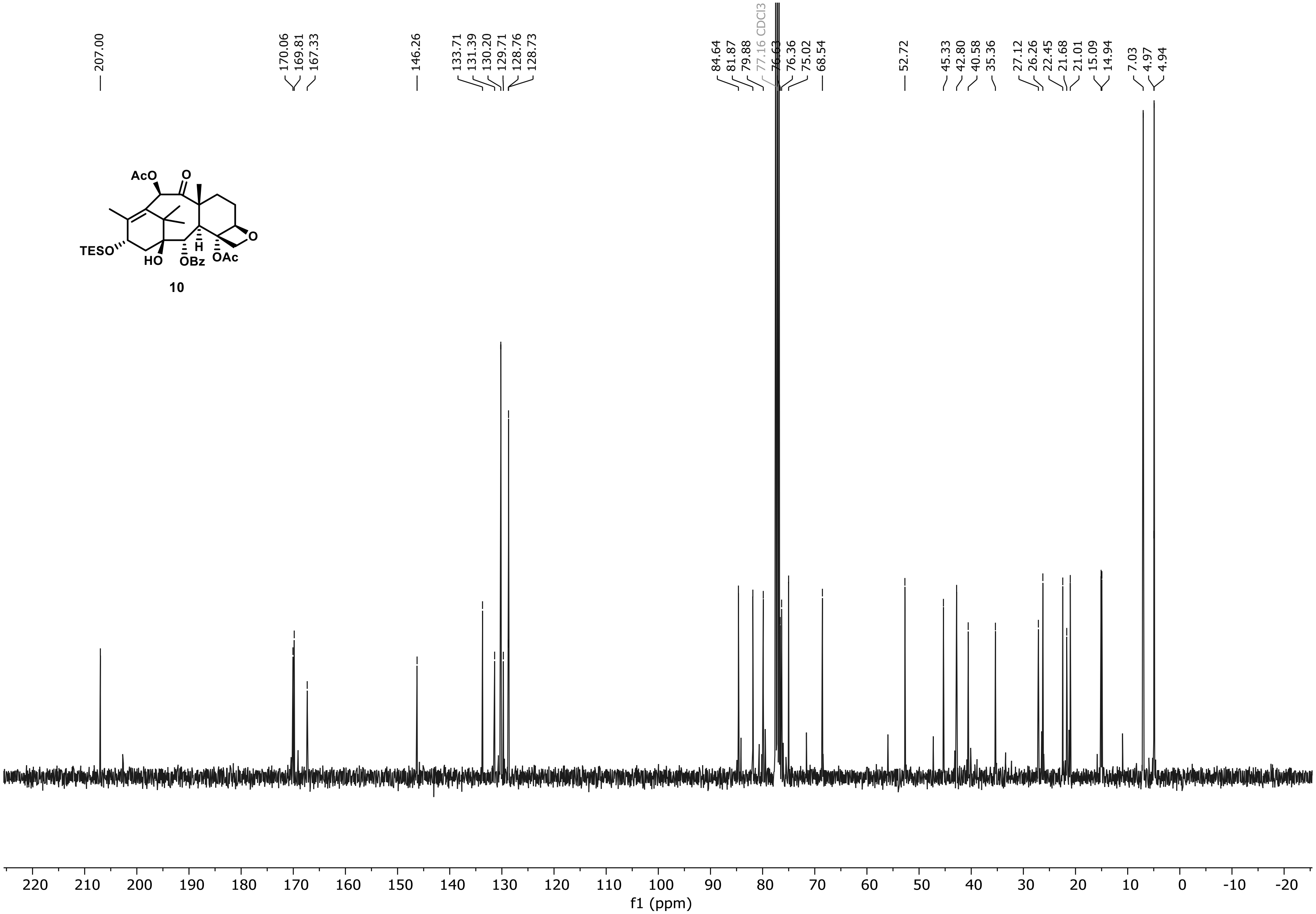

^{1}H NMR (400 MHz, $CDCl_3$)

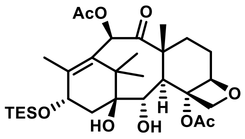

11

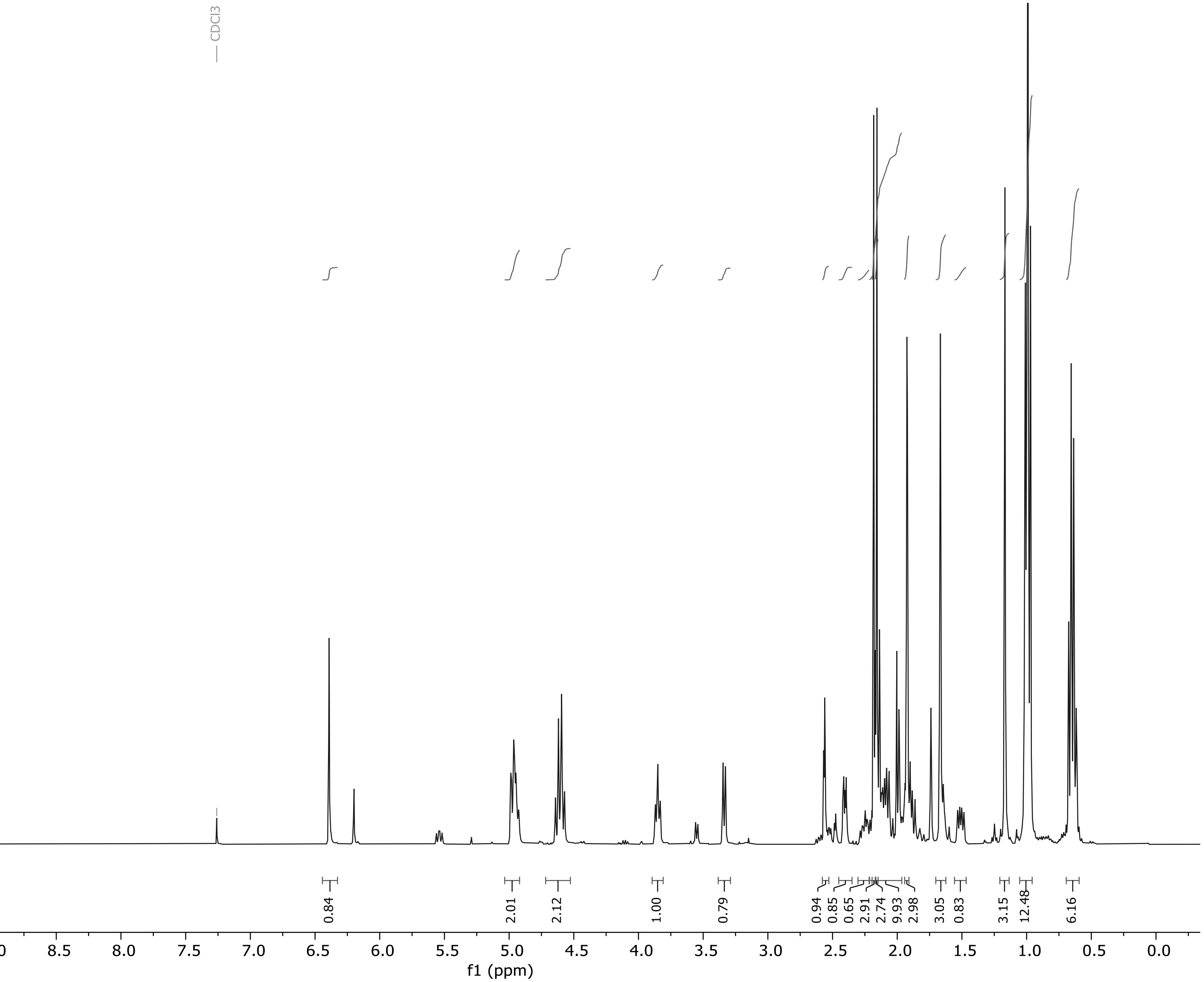

^{13}C NMR (101 MHz, $CDCl_3$)

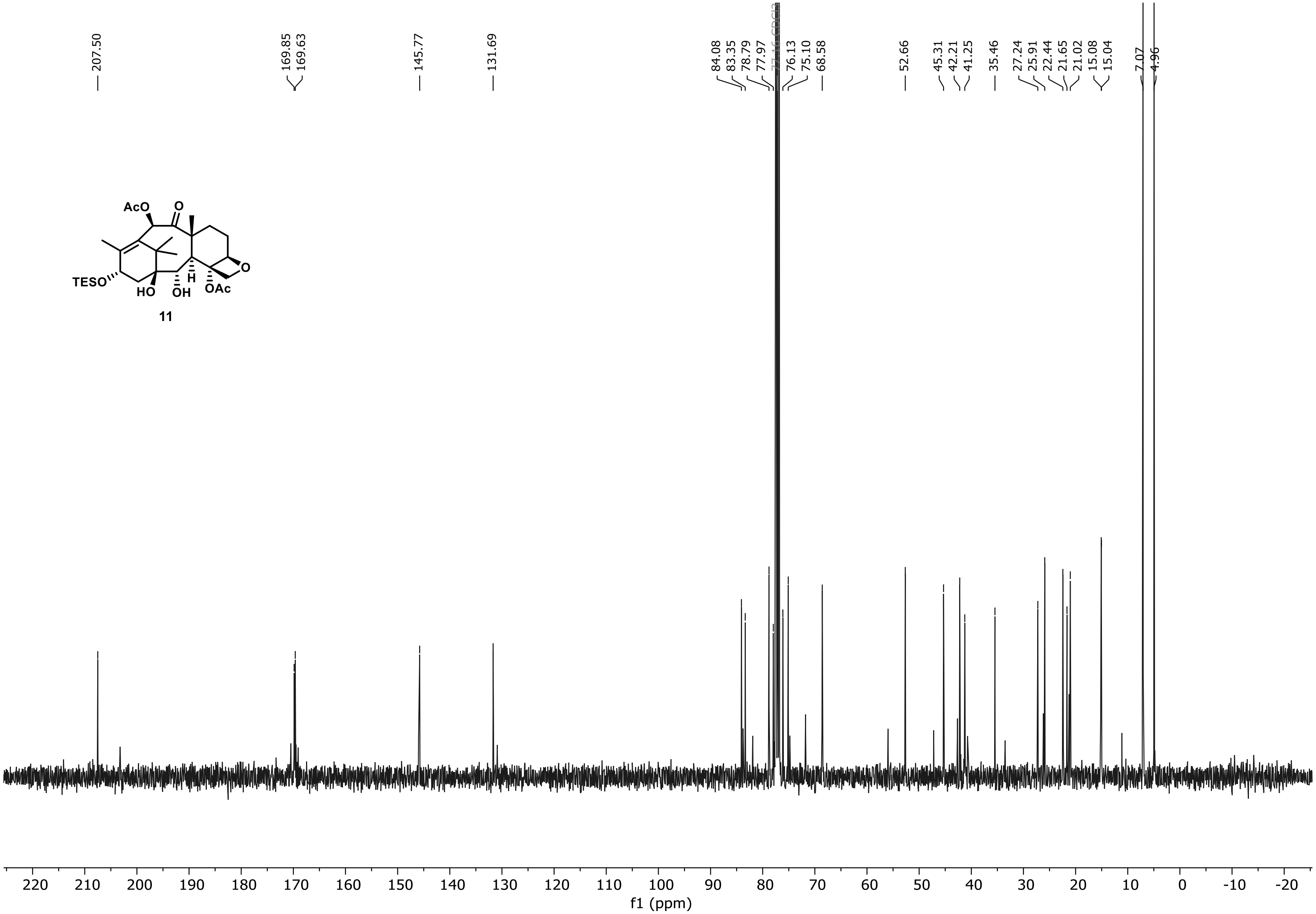

11

^{1}H NMR (400 MHz, $CDCl_3$)

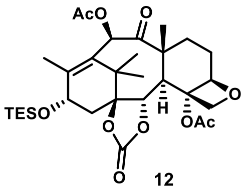

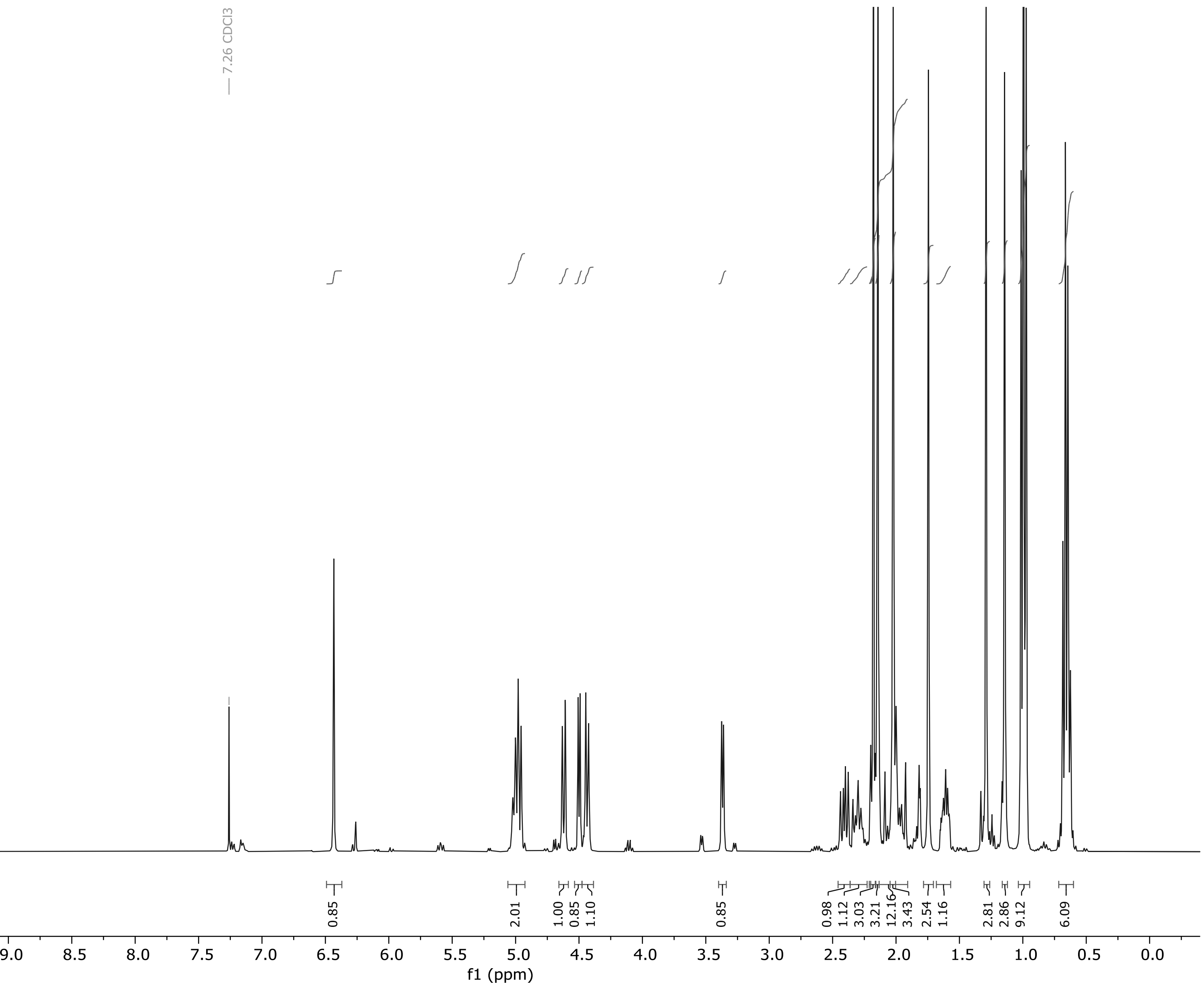

^{13}C NMR (101 MHz, $CDCl_3$)

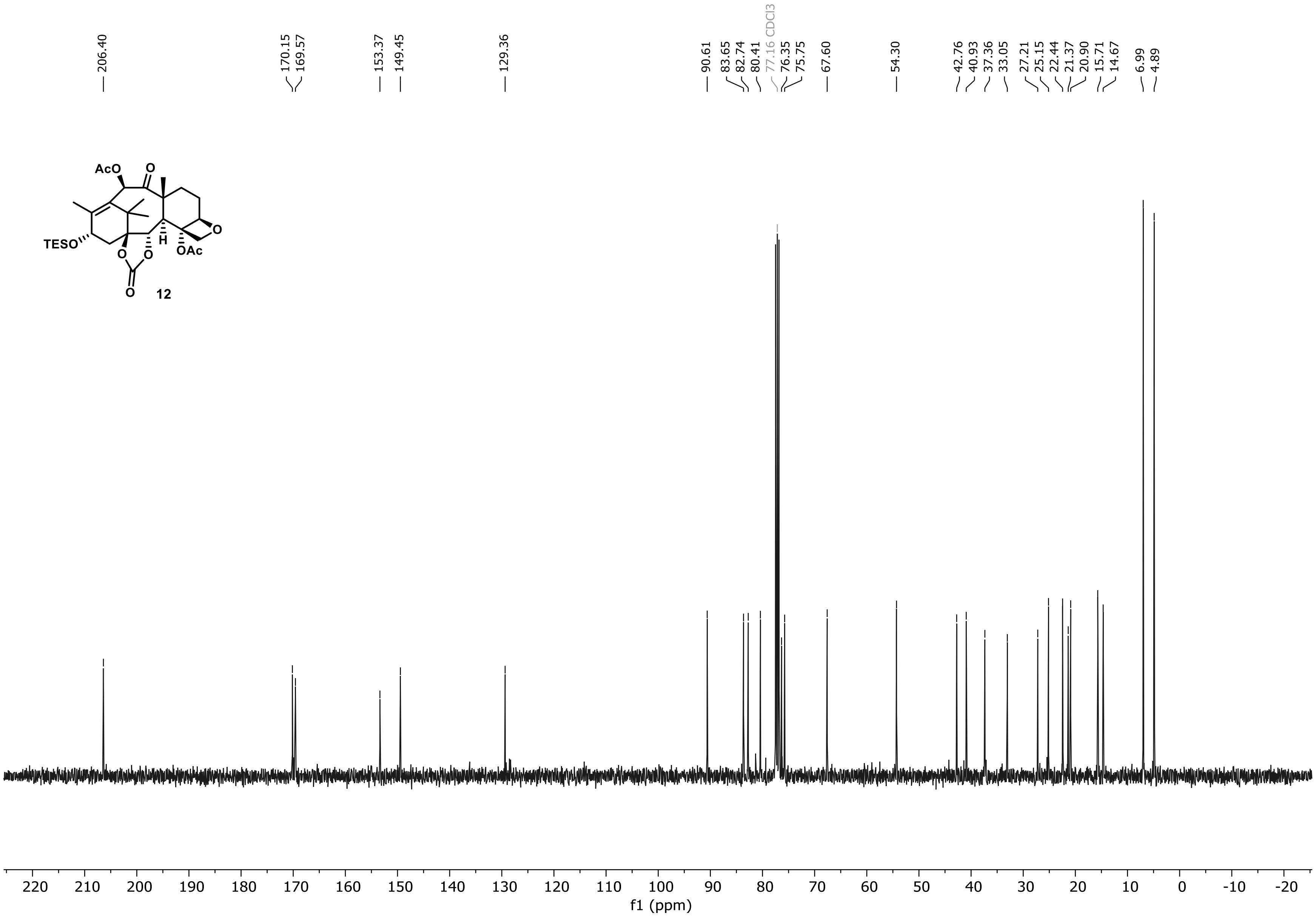

^{1}H NMR (400 MHz, $CDCl_3$)

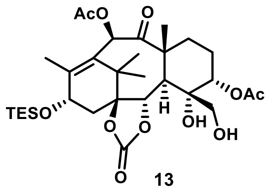

13

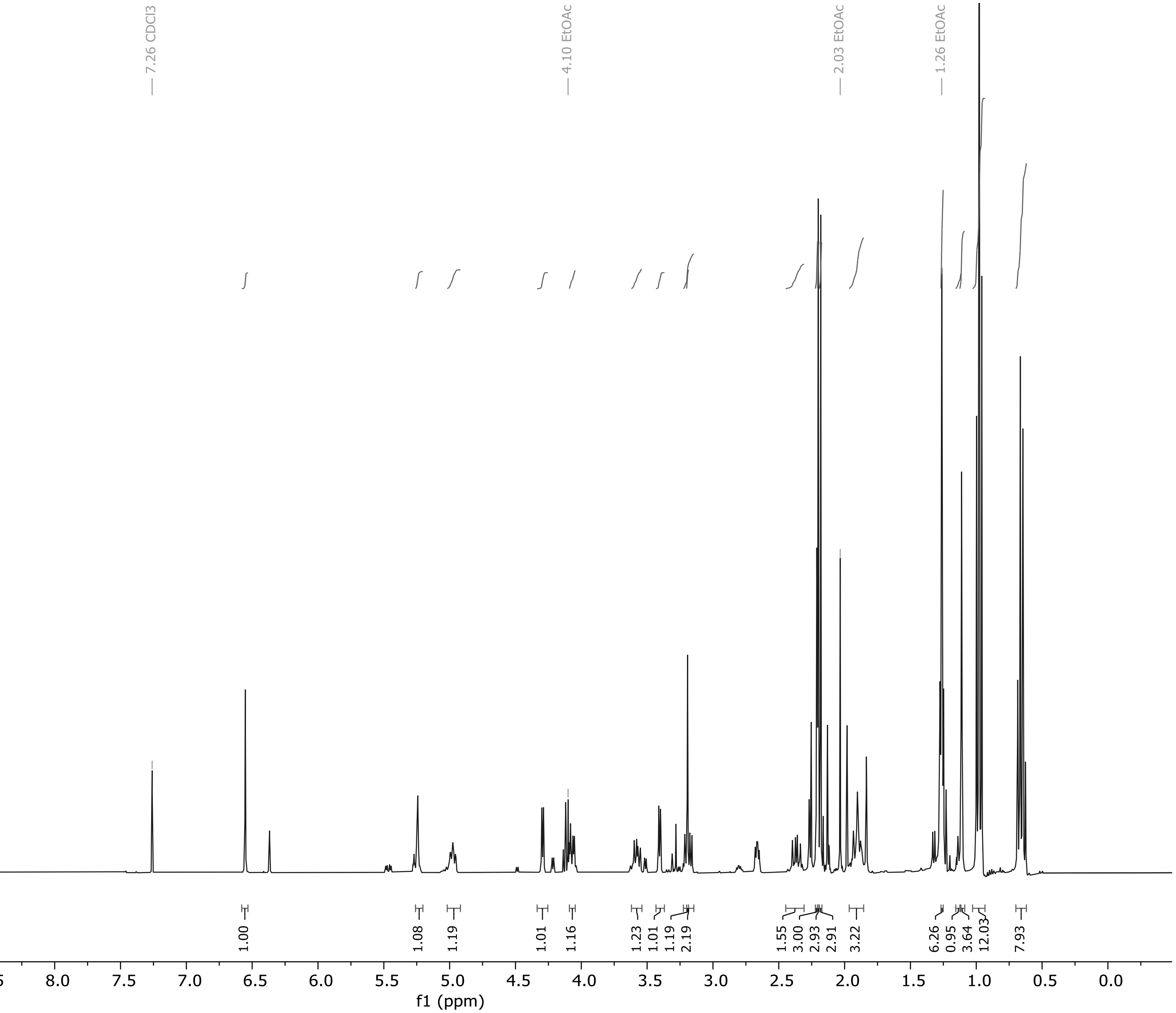

^{13}C NMR (101 MHz, $CDCl_3$)

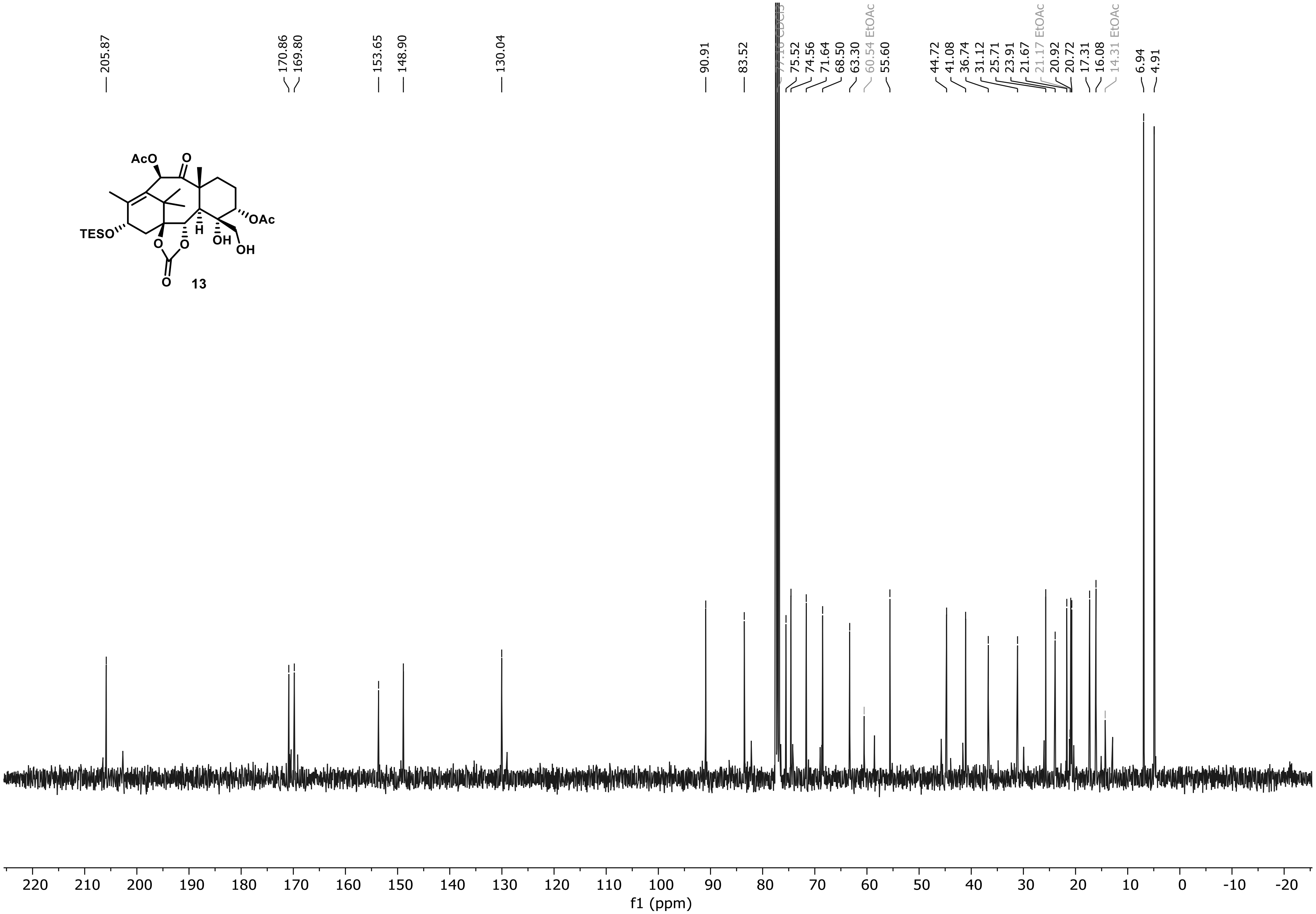

^1H NMR (400 MHz, $CDCl_3$)

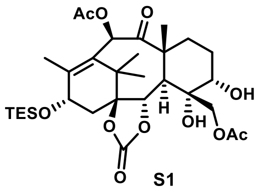

S1

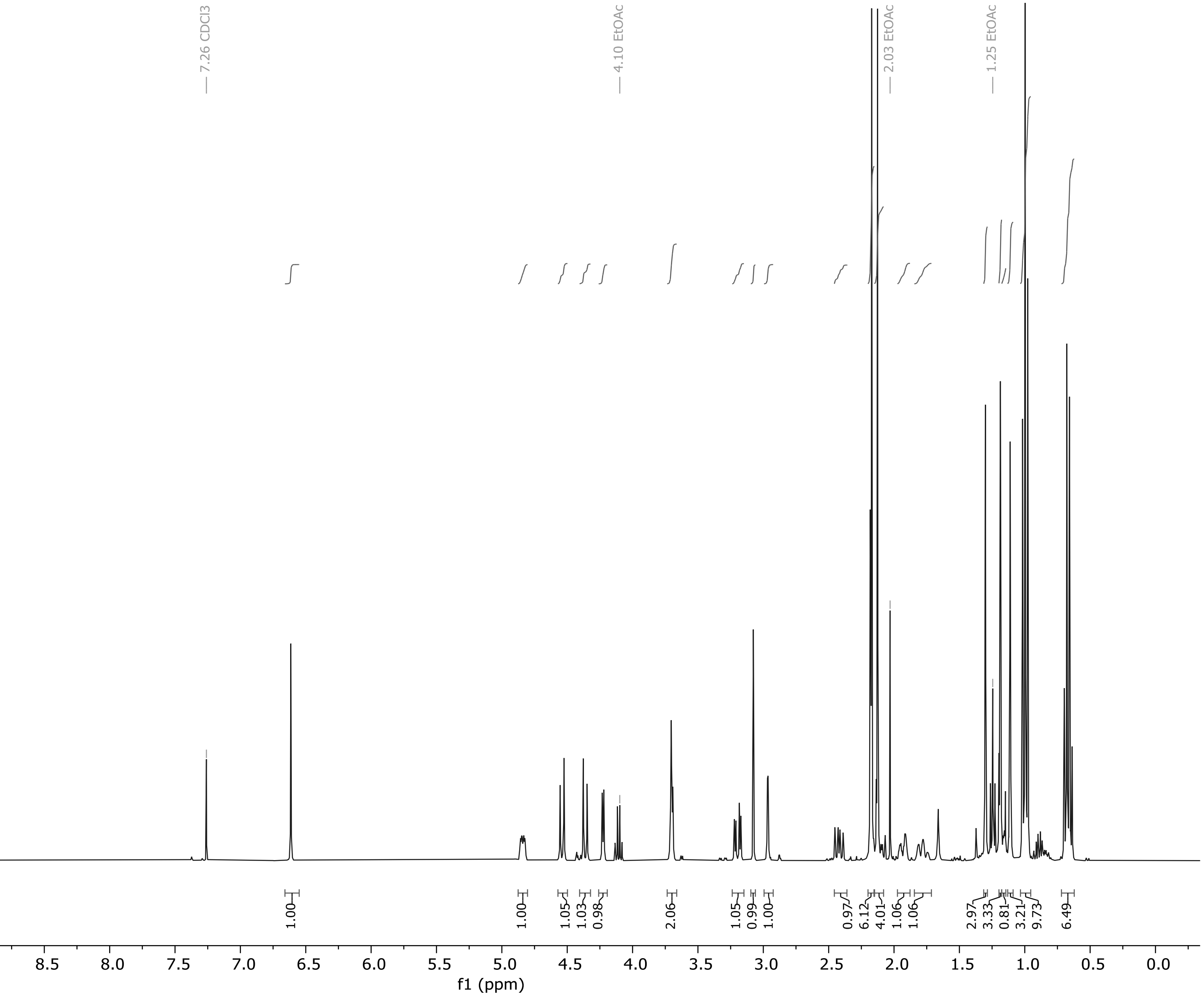

^{13}C NMR (101 MHz, $CDCl_3$)

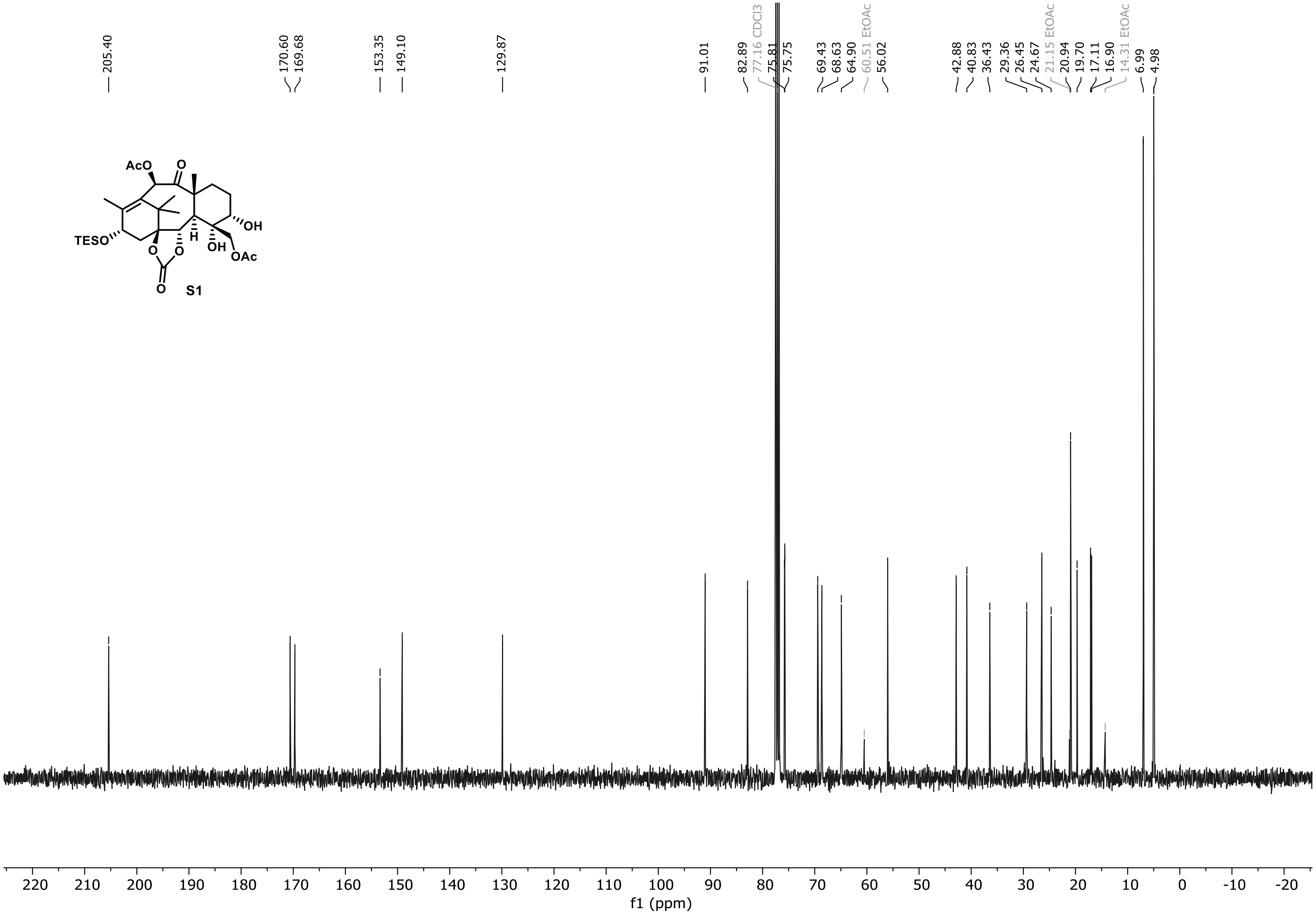

^{1}H NMR (400 MHz, $CDCl_3$)

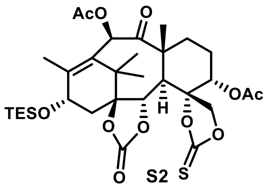

S2

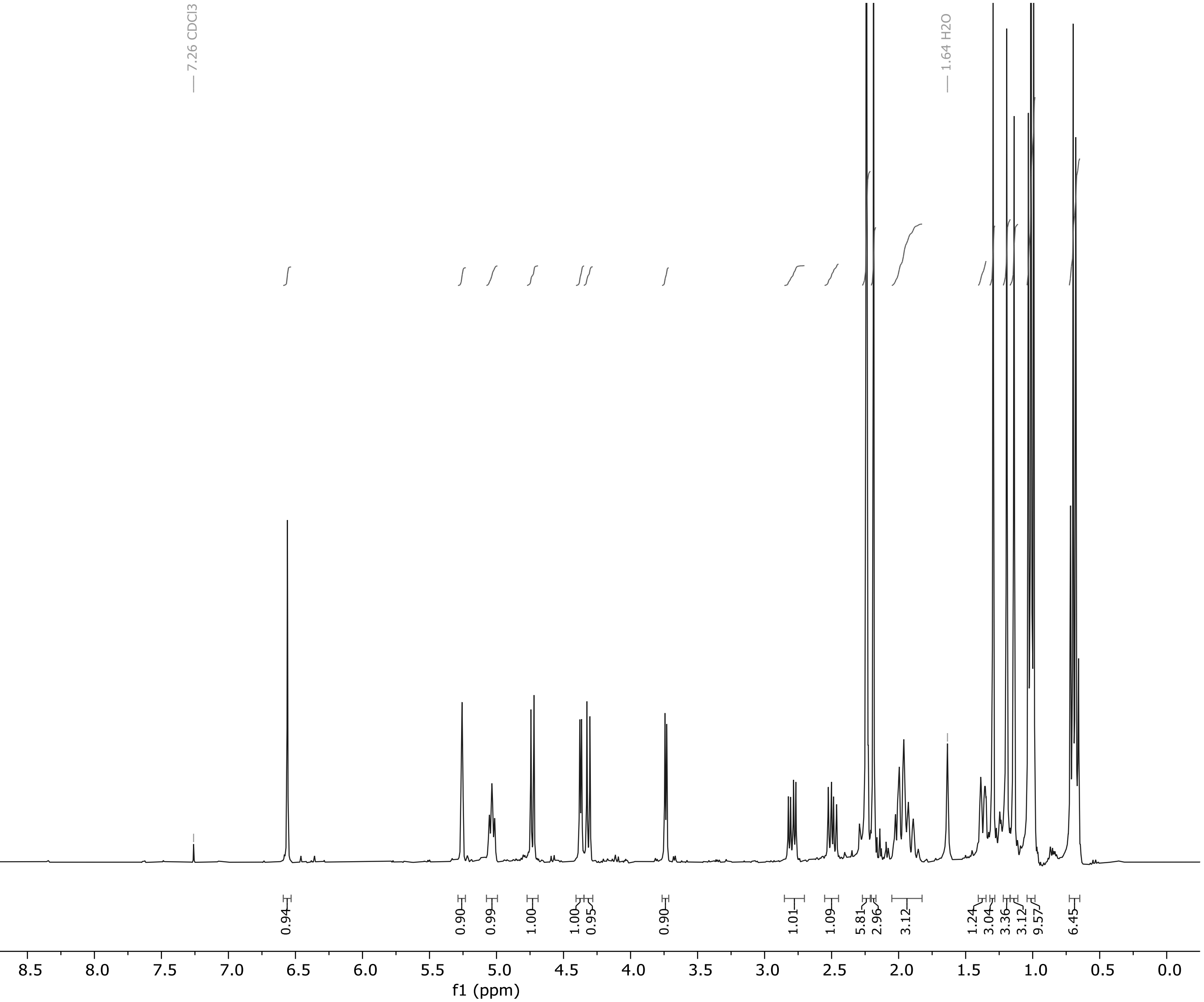

^{13}C NMR (101 MHz, CDCl$_3$)

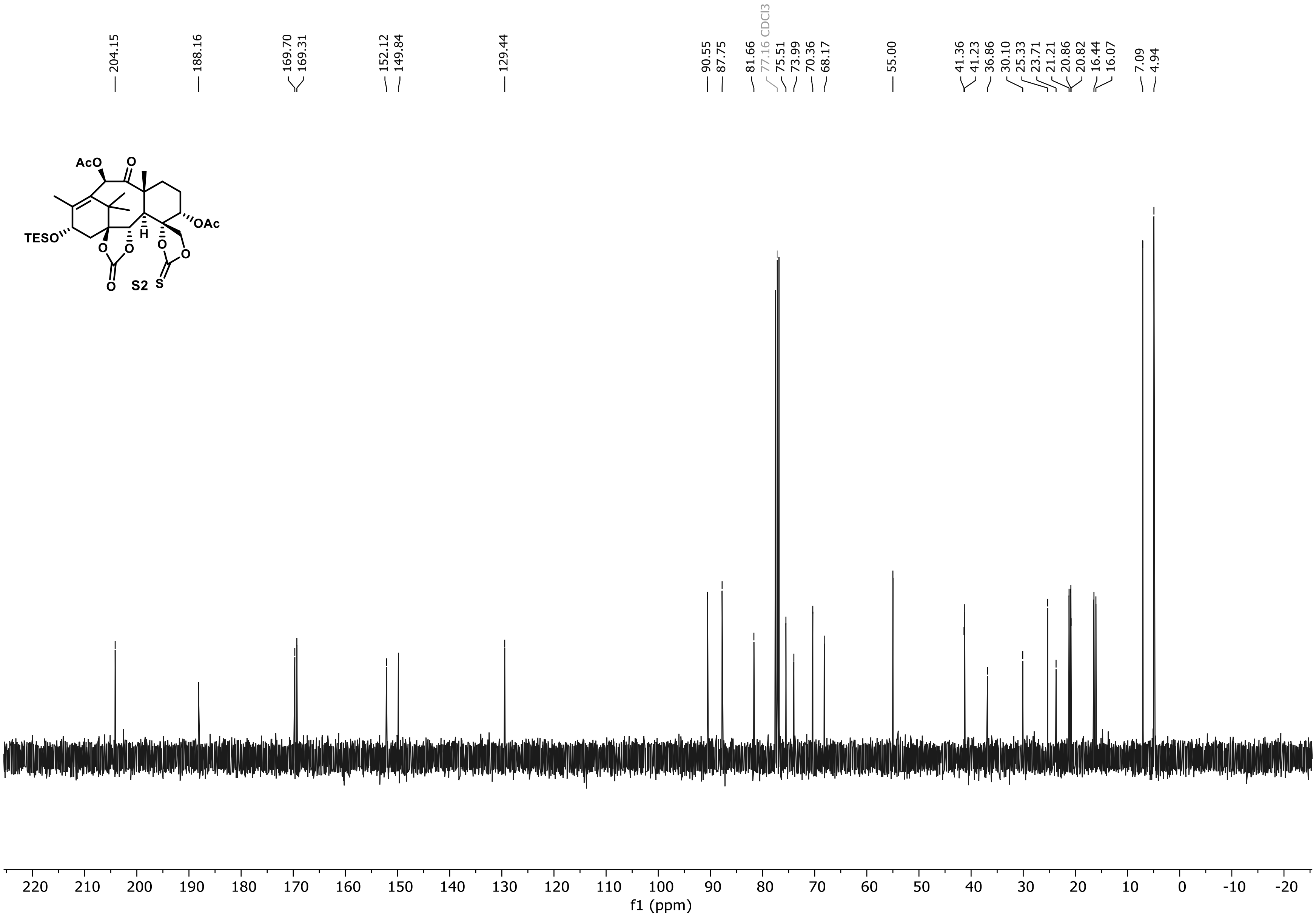

^{1}H NMR (400 MHz, $CDCl_3$)

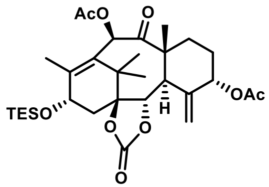
14

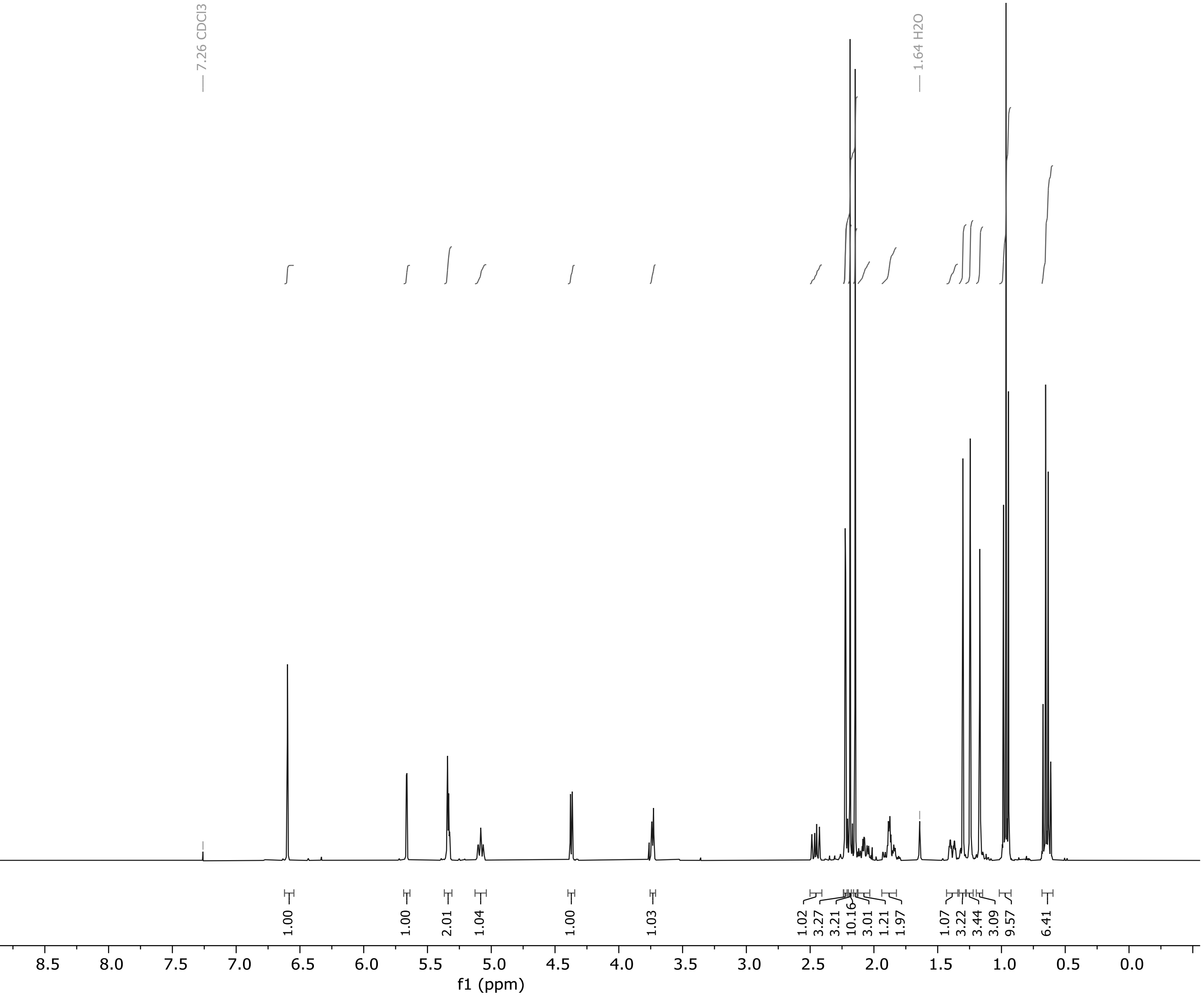

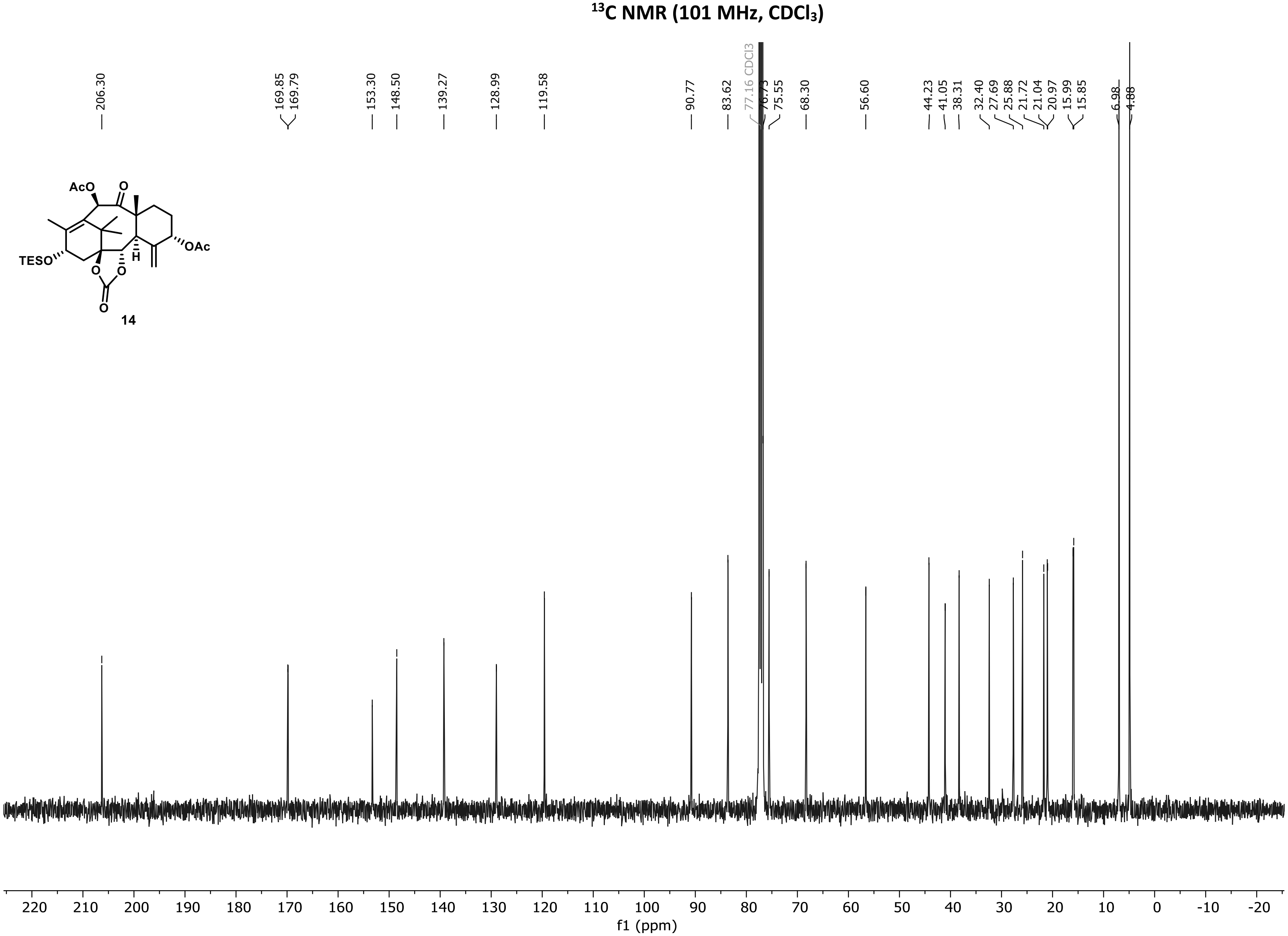

13C NMR (101 MHz, CDCl3)
206.30
169.85
169.79
153.30
148.50
139.27
128.99
119.58
90.77
83.62
77.16 CDCl3
76.73
75.55
68.30
56.60
44.23
41.05
38.31
32.40
27.69
25.88
21.72
21.04
20.97
15.99
15.85
6.98
4.88
AcO
O
OAc
TESO
H
O
O
O
14
220 210 200 190 180 170 160 150 140 130 120 110 100 90 80 70 60 50 40 30 20 10 0 -10 -20
f1 (ppm)

^1H NMR (400 MHz, $CDCl_3$)

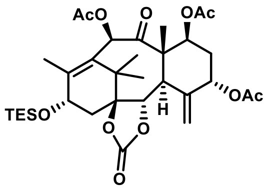

S3

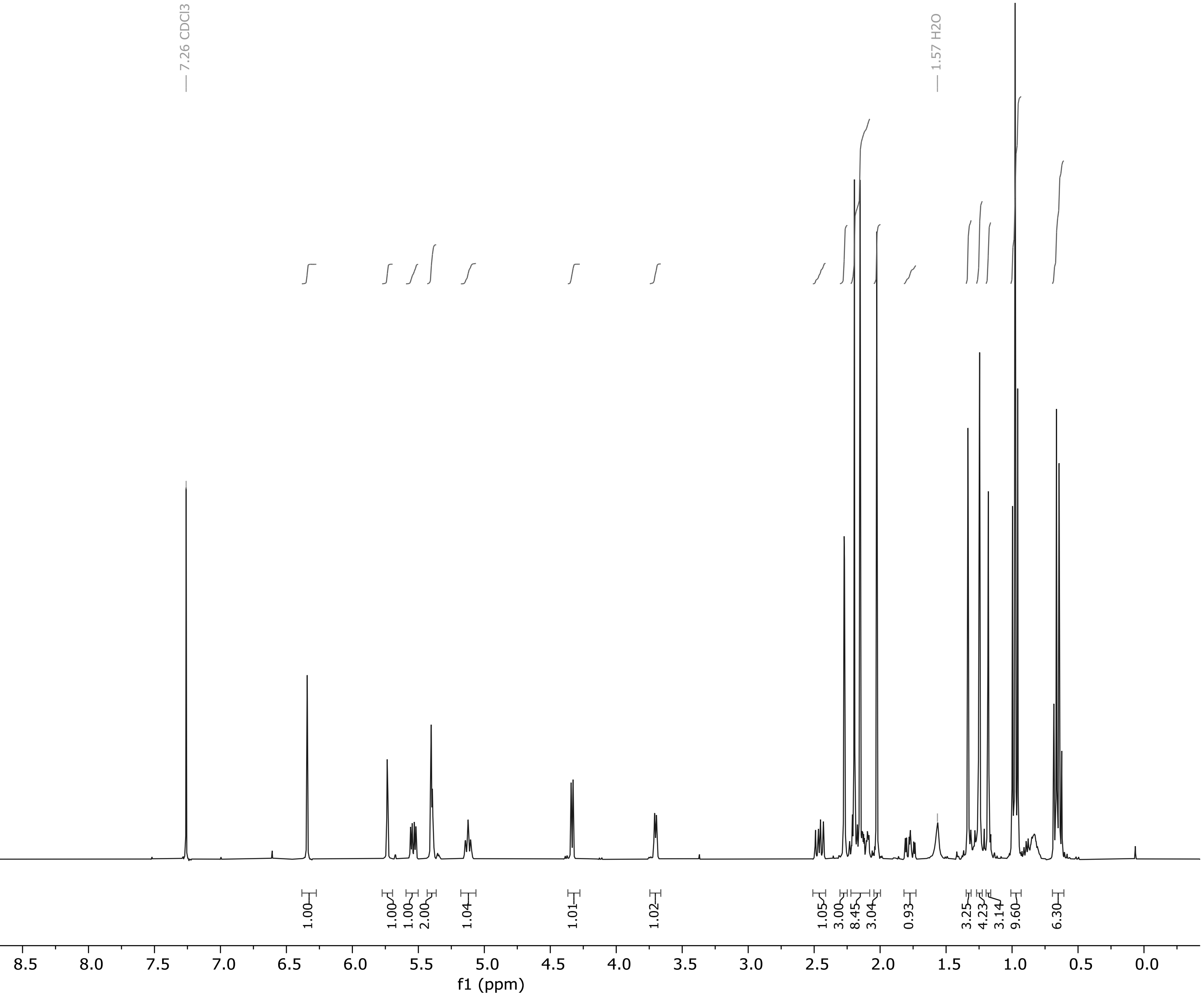

^{13}C NMR (101 MHz, CDCl$_3$)

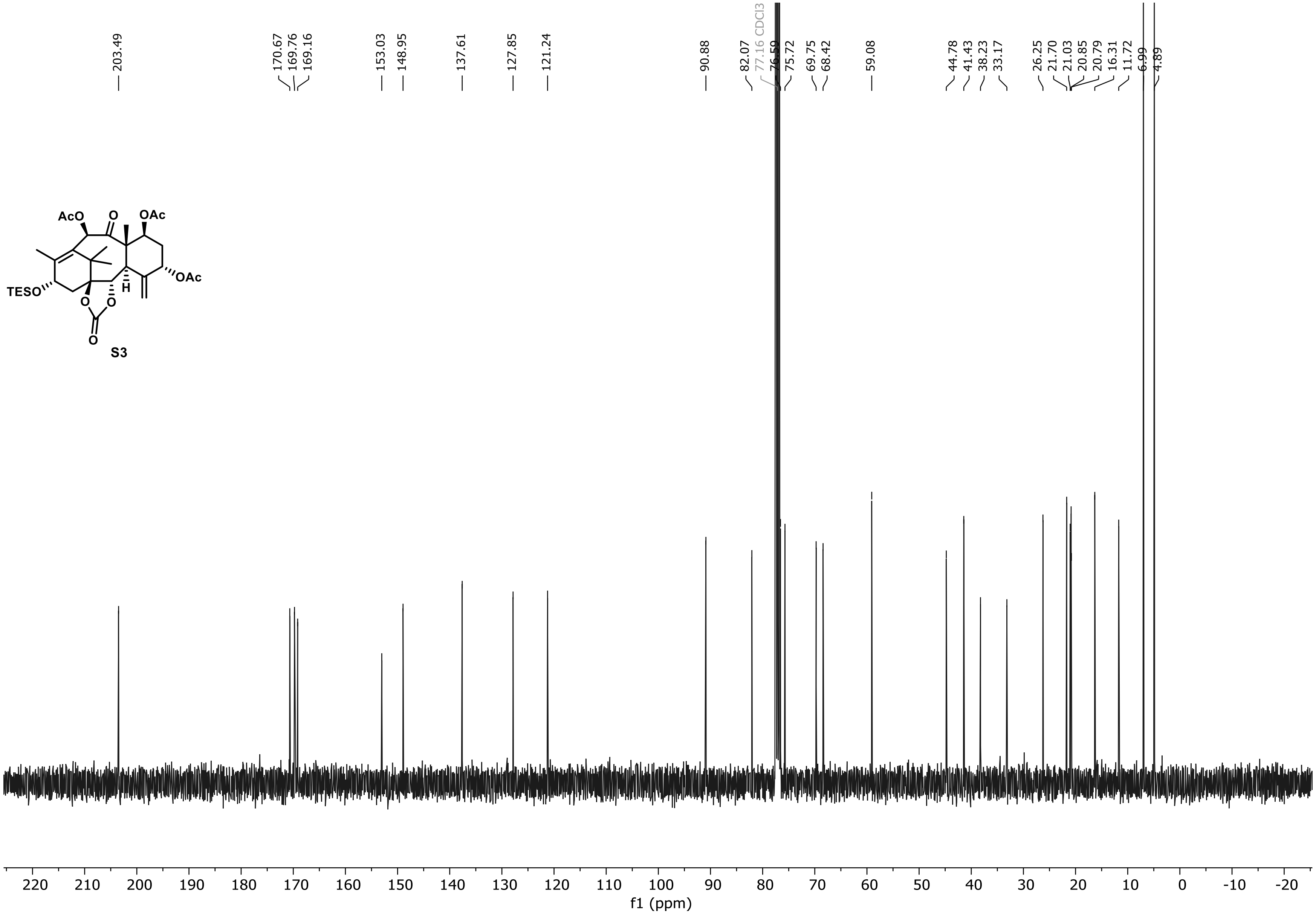

^{1}H NMR (400 MHz, $CDCl_3$)

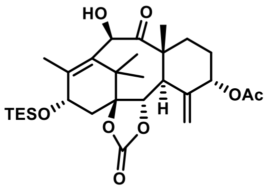

15

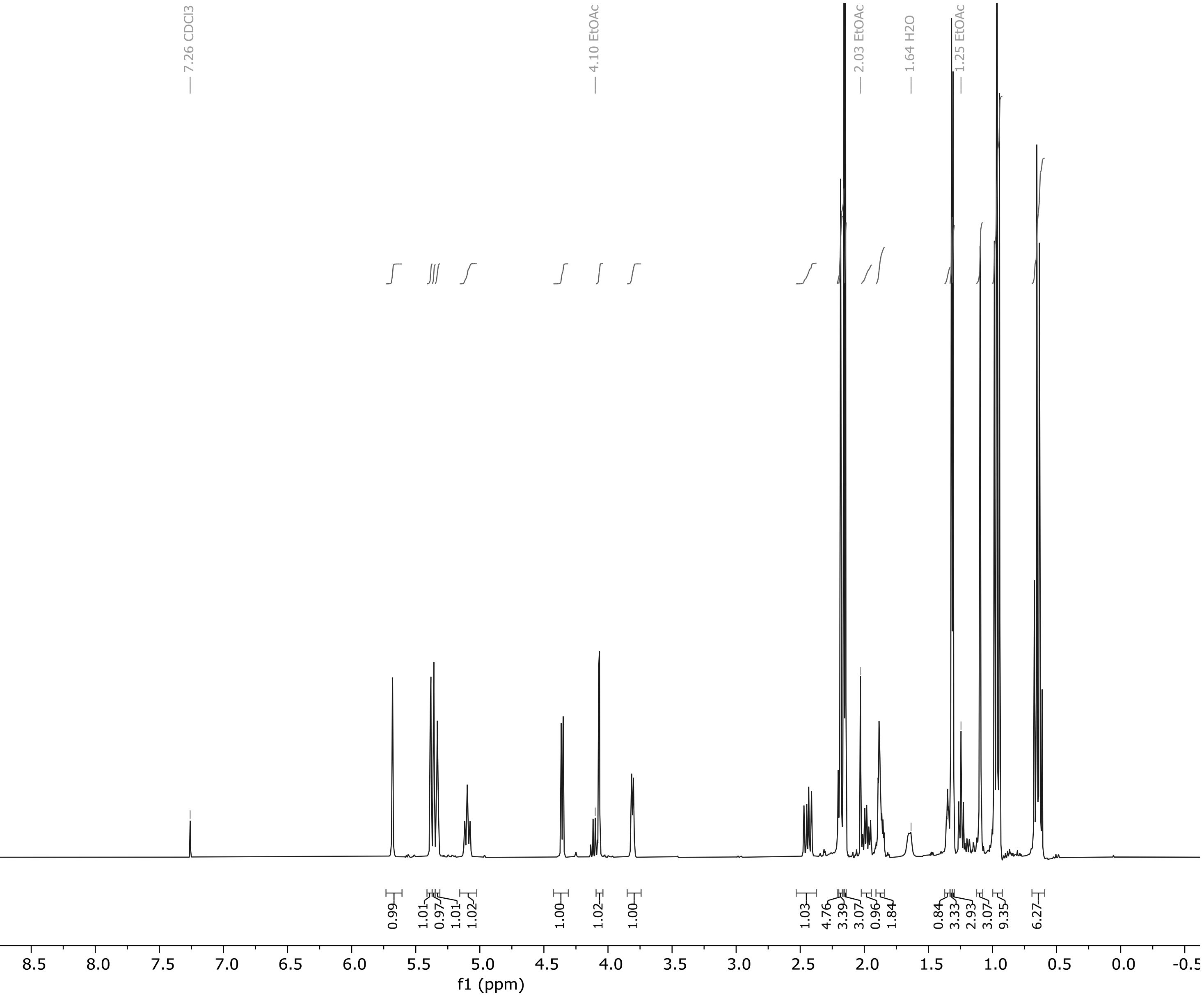

^{13}C NMR (101 MHz, $CDCl_3$)

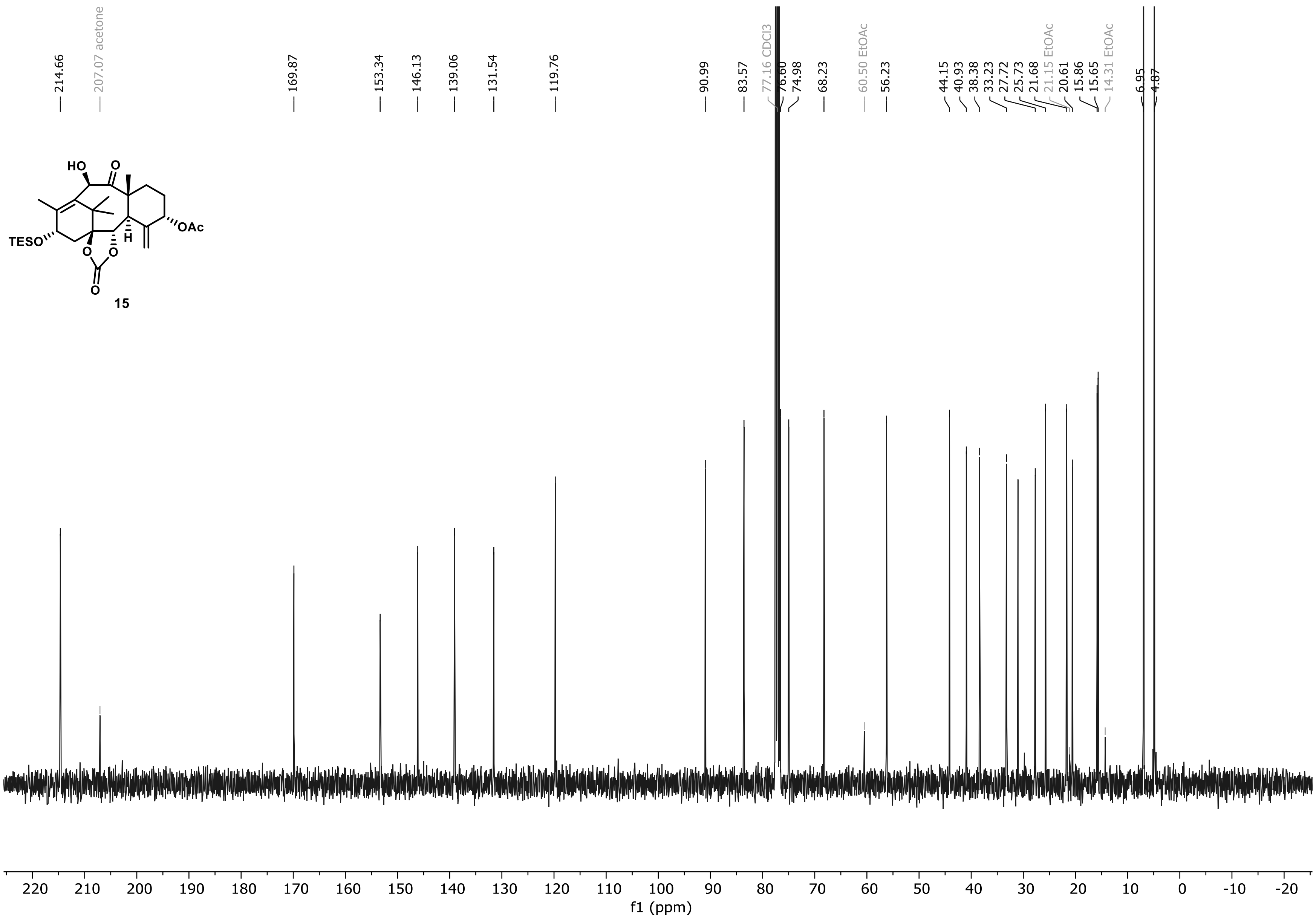

^{1}H NMR (400 MHz, CDCl$_3$)

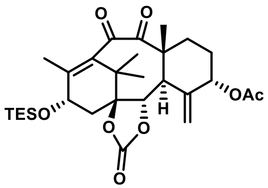
S5

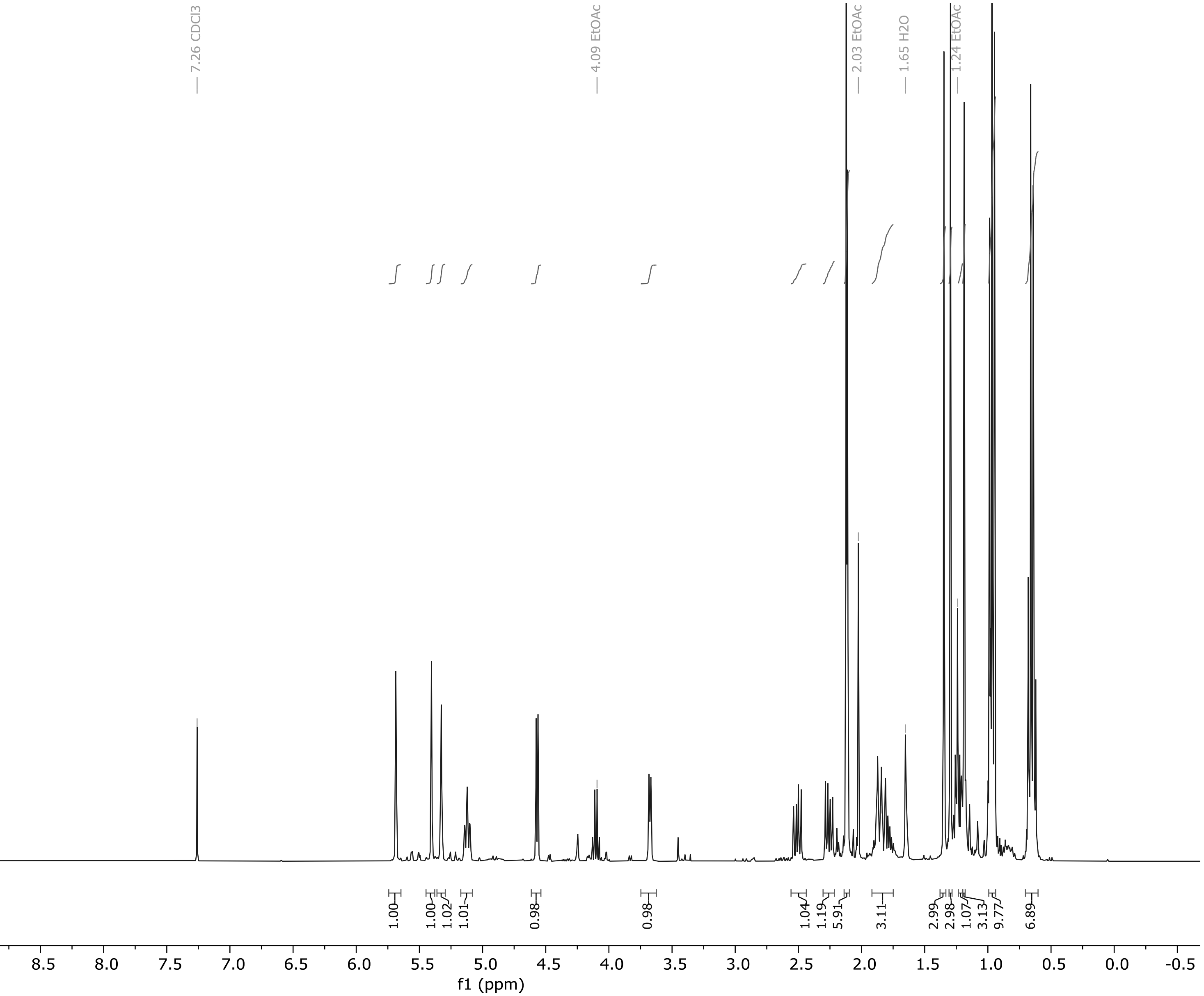

^{13}C NMR (101 MHz, $CDCl_3$)

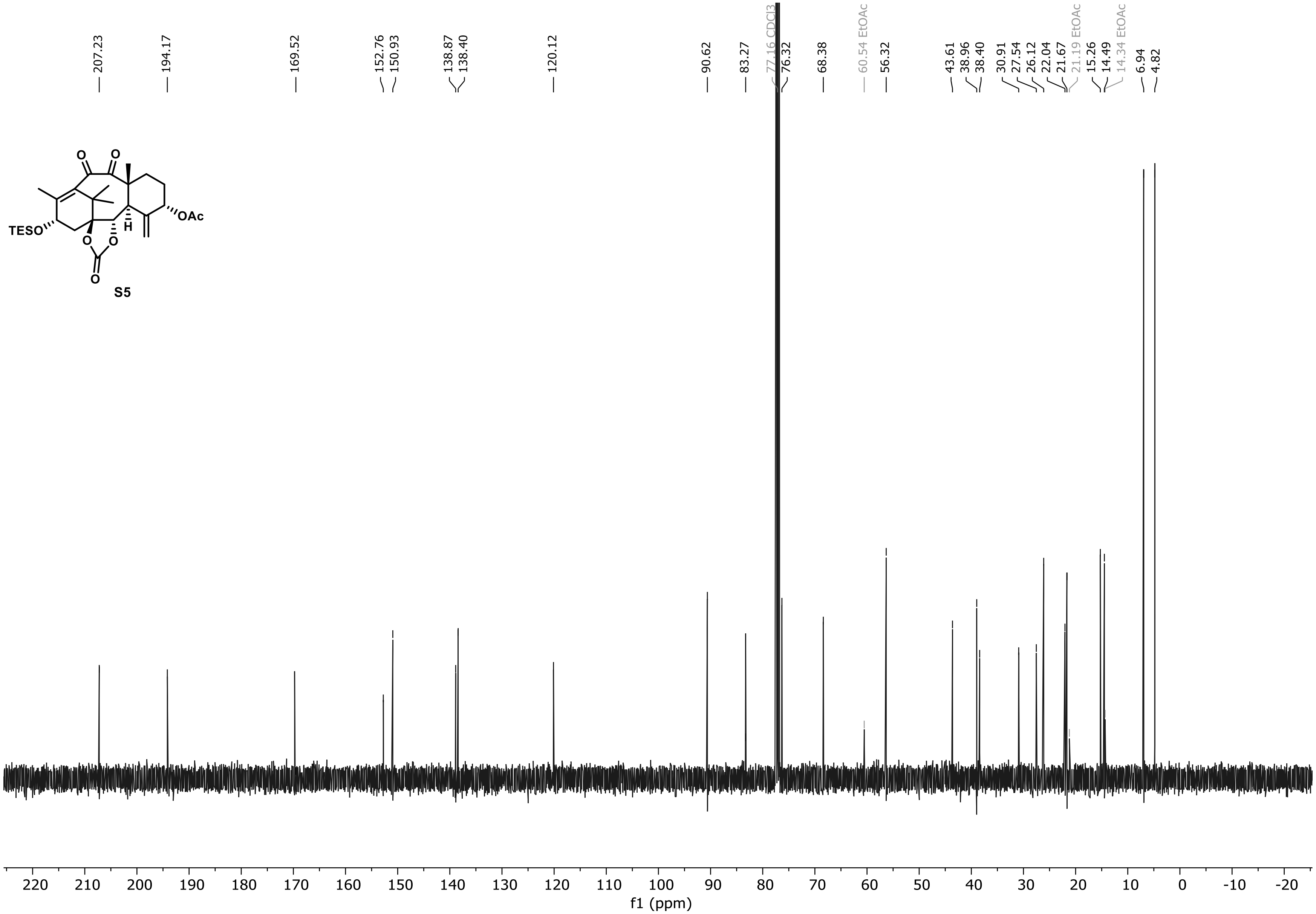

^{1}H NMR (400 MHz, $CDCl_3$)

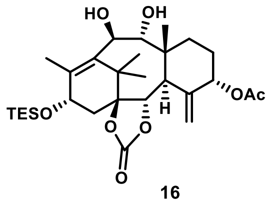

16

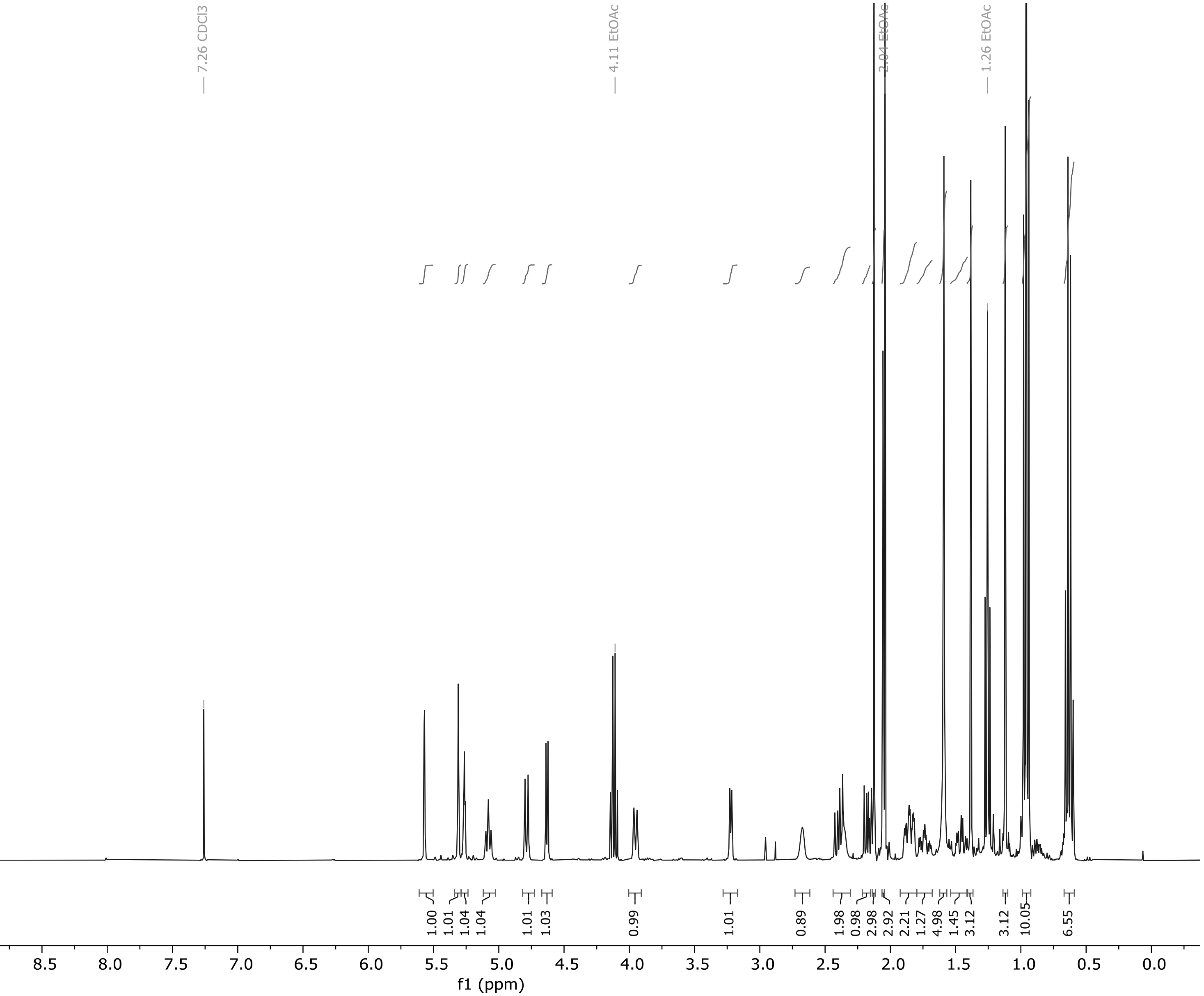

^{13}C NMR (101 MHz, $CDCl_3$)

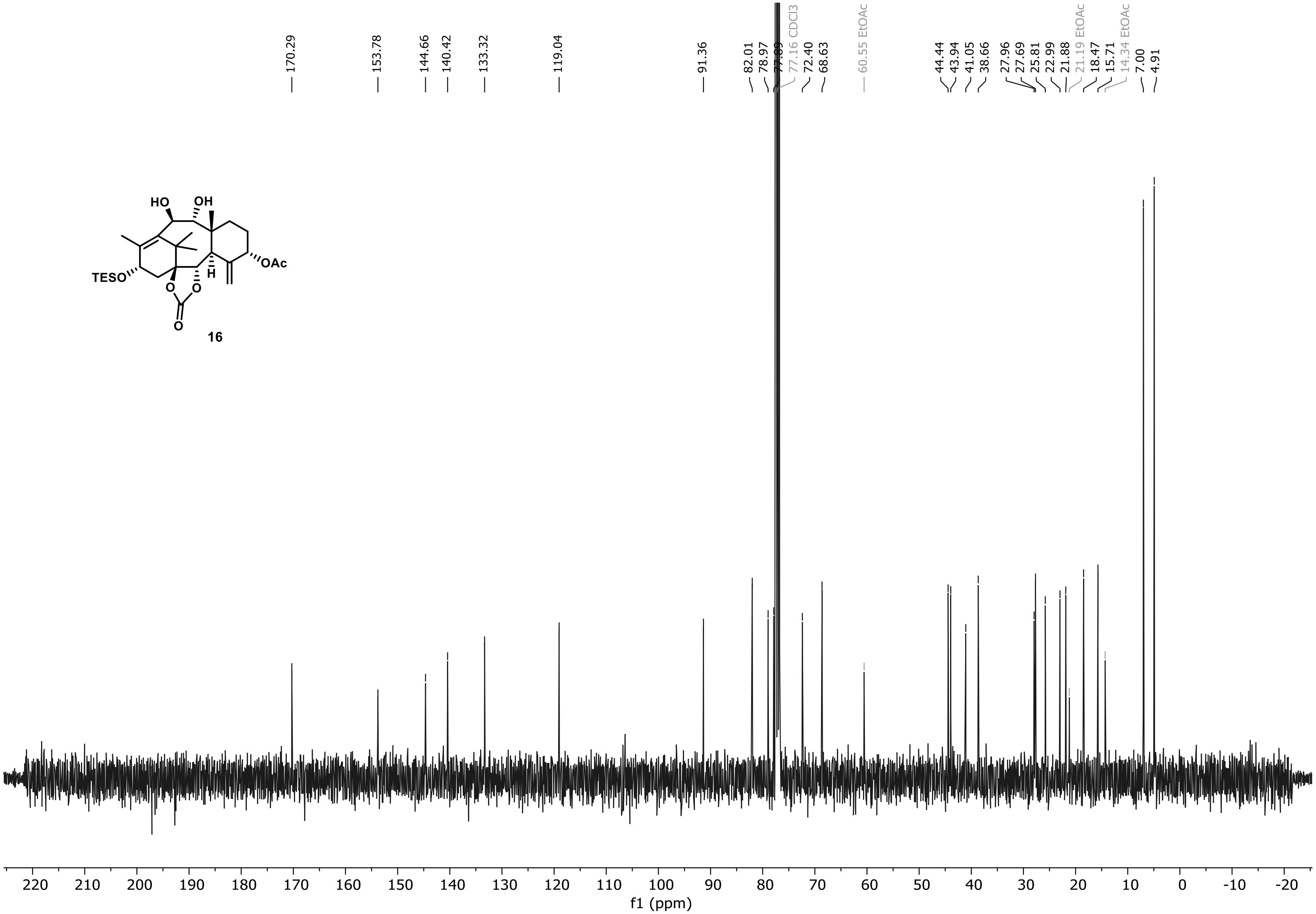

^{1}H NMR (400 MHz, CDCl$_3$)

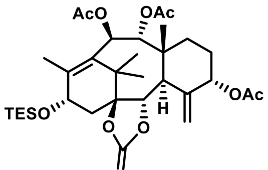

17

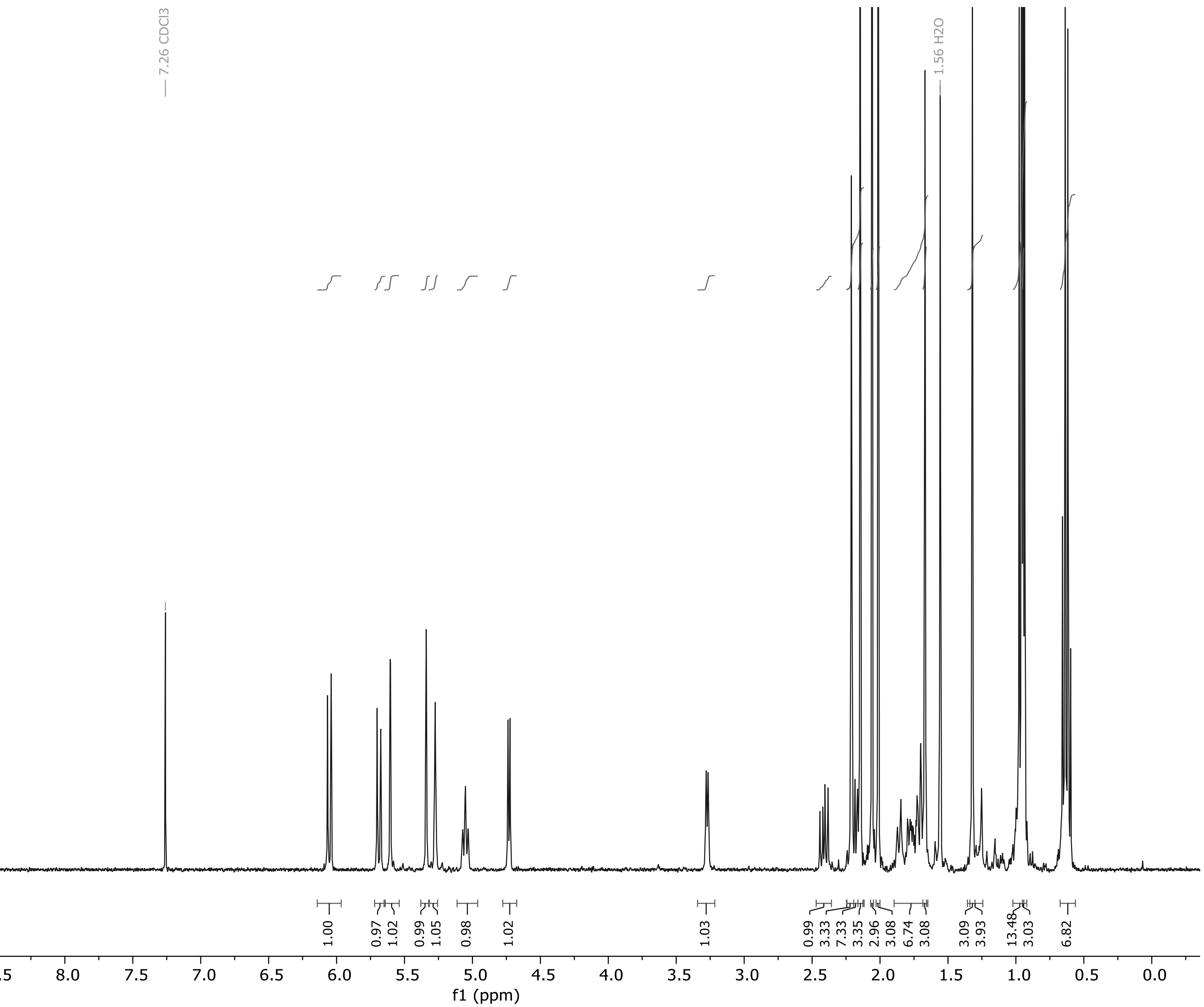

^{13}C NMR (101 MHz, $CDCl_3$)

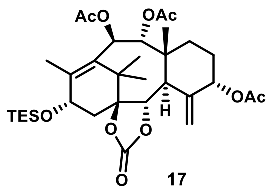

17

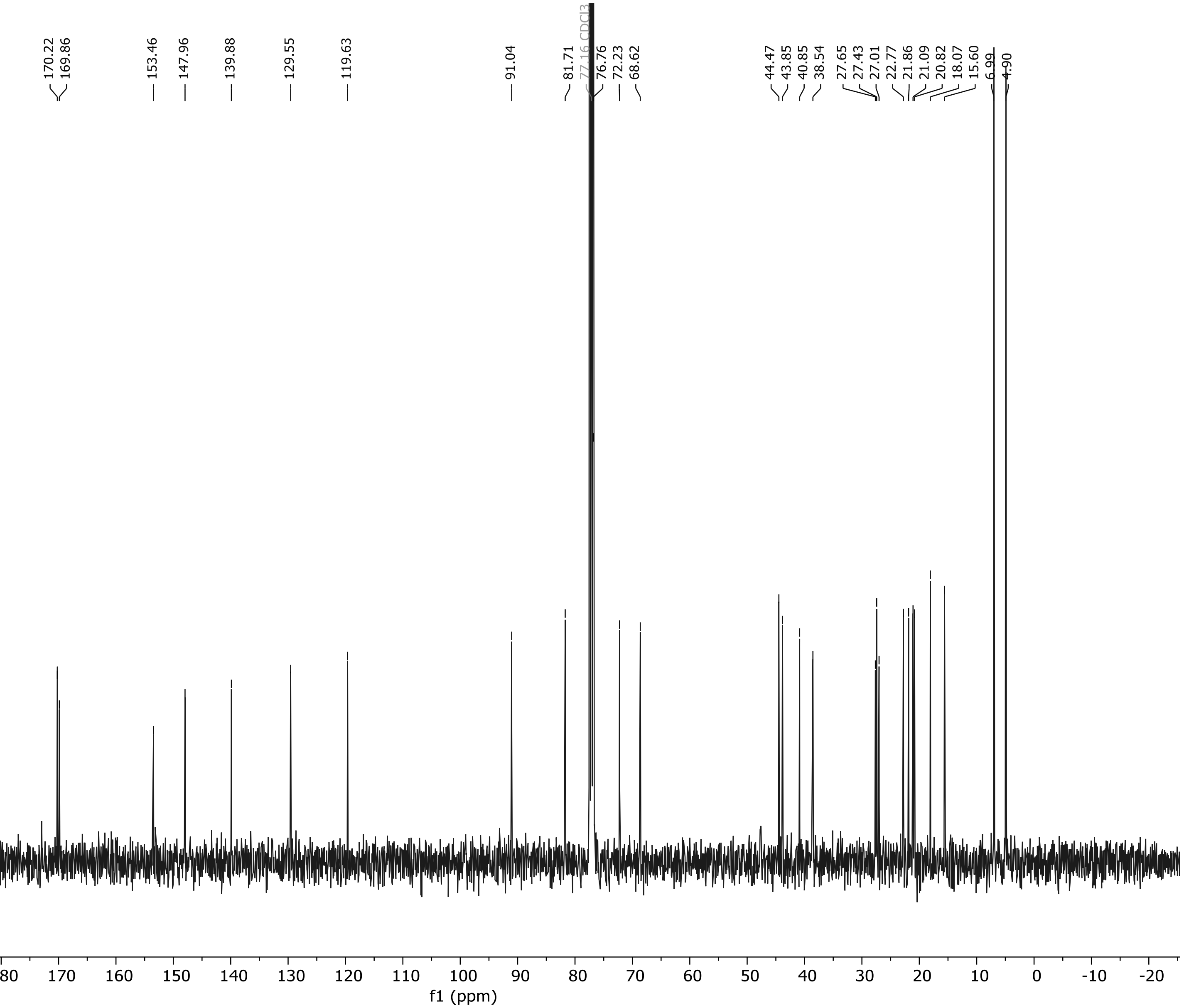

^{1}H NMR (400 MHz, $CDCl_3$)

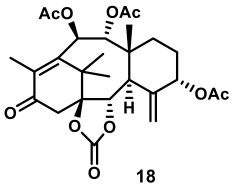

18

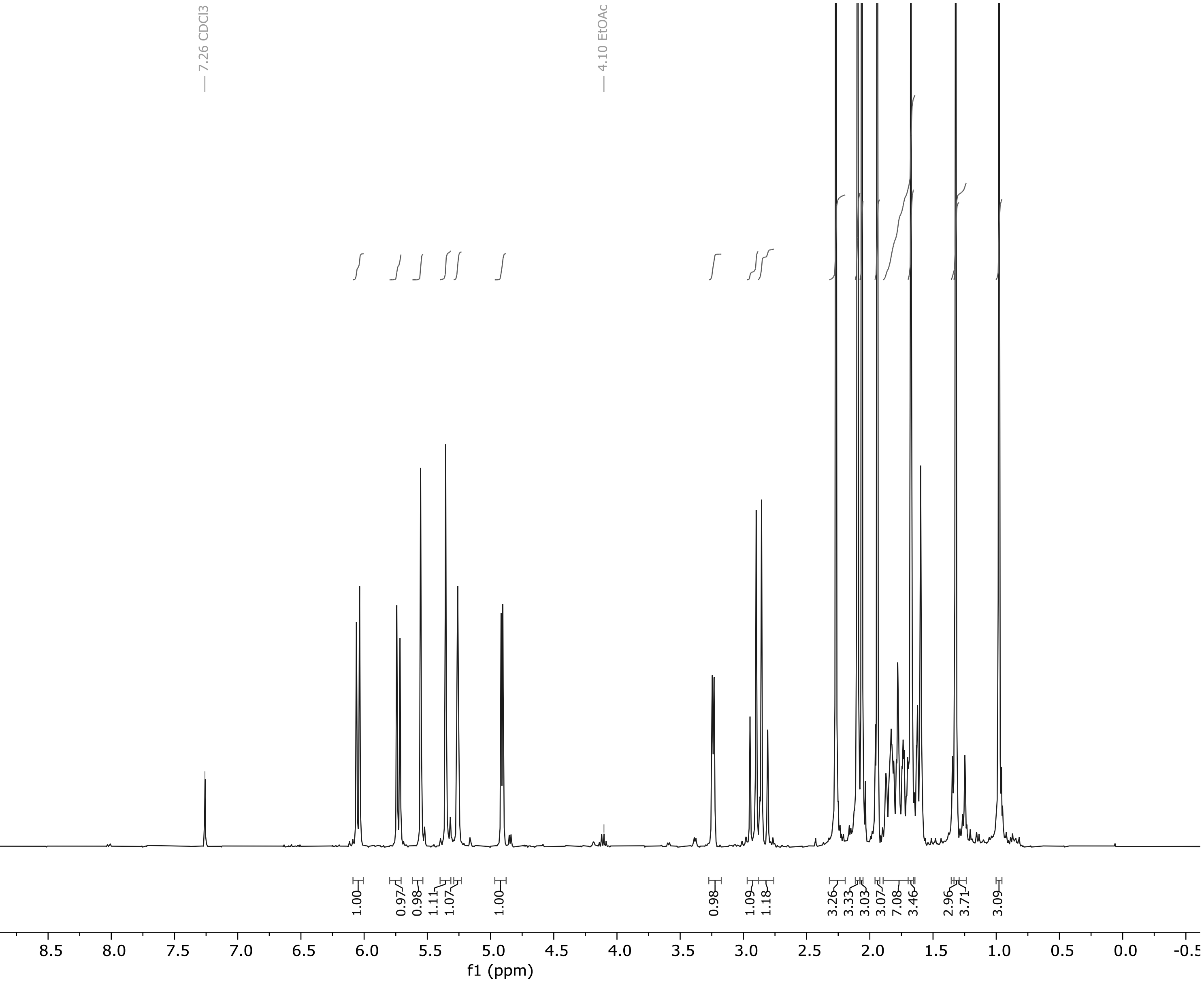

^{13}C NMR (101 MHz, $CDCl_3$)

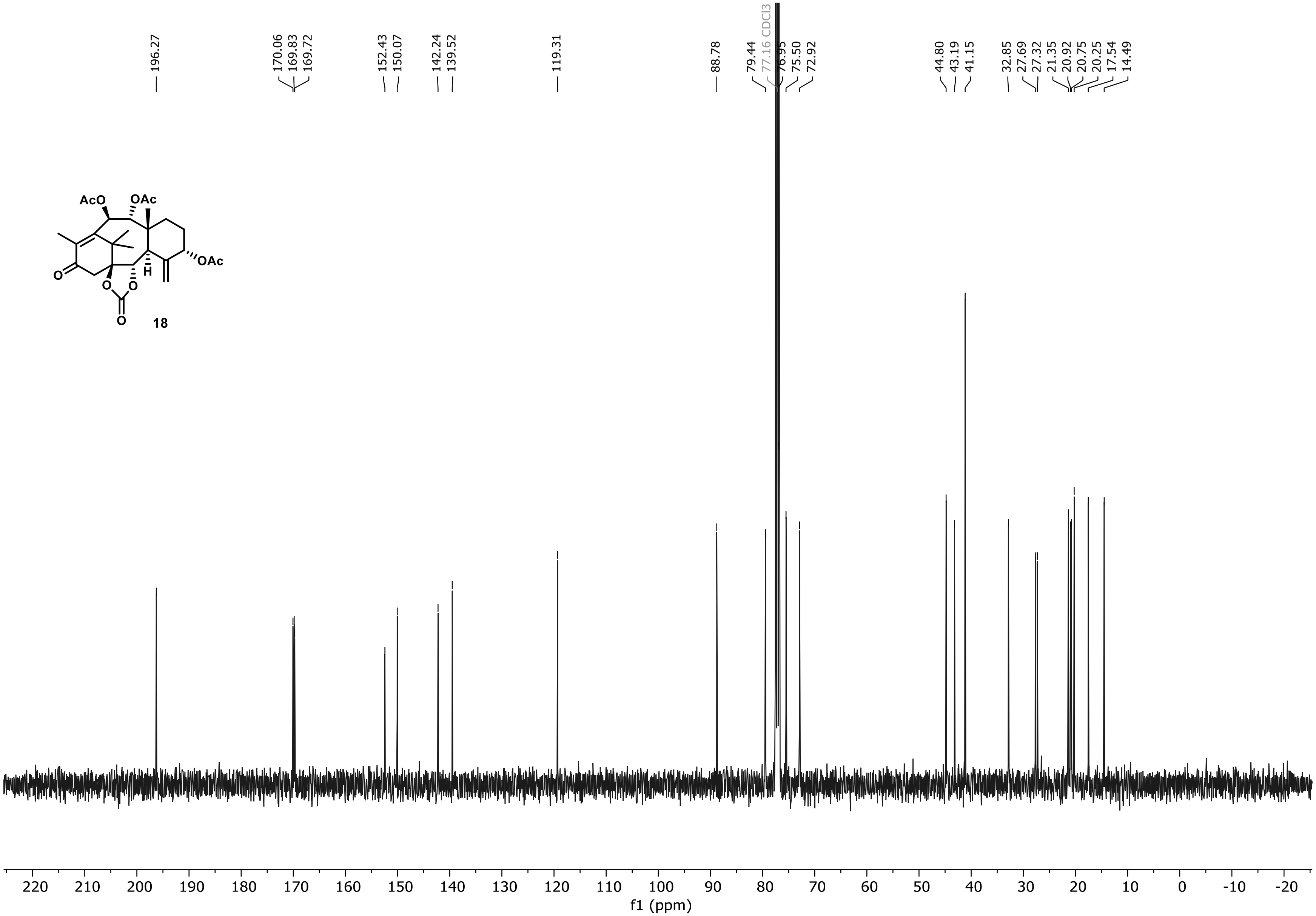

^{1}H NMR (400 MHz, CDCl$_3$)

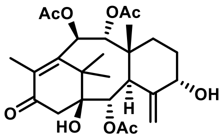

19

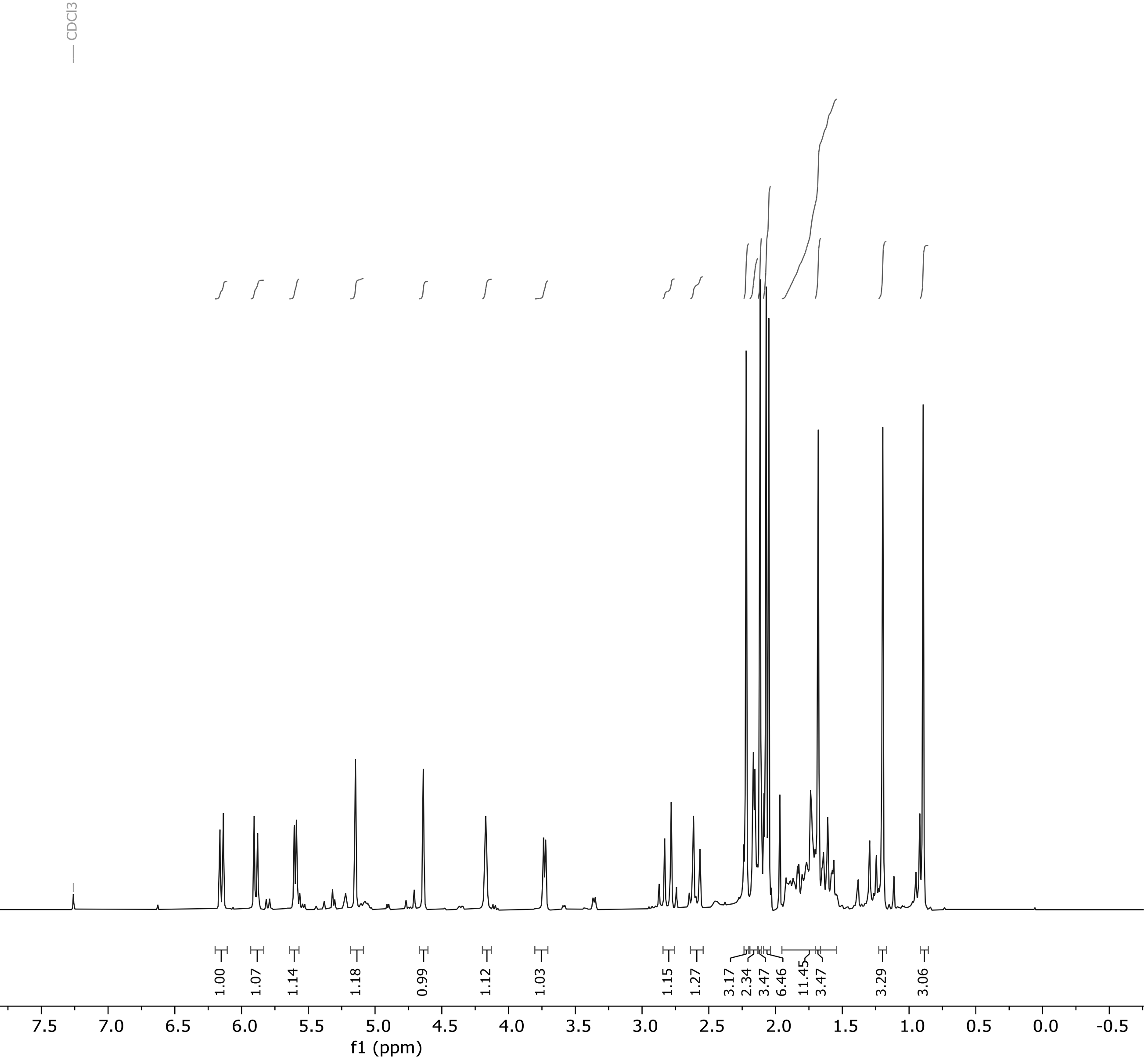

^{13}C NMR (101 MHz, $CDCl_3$)

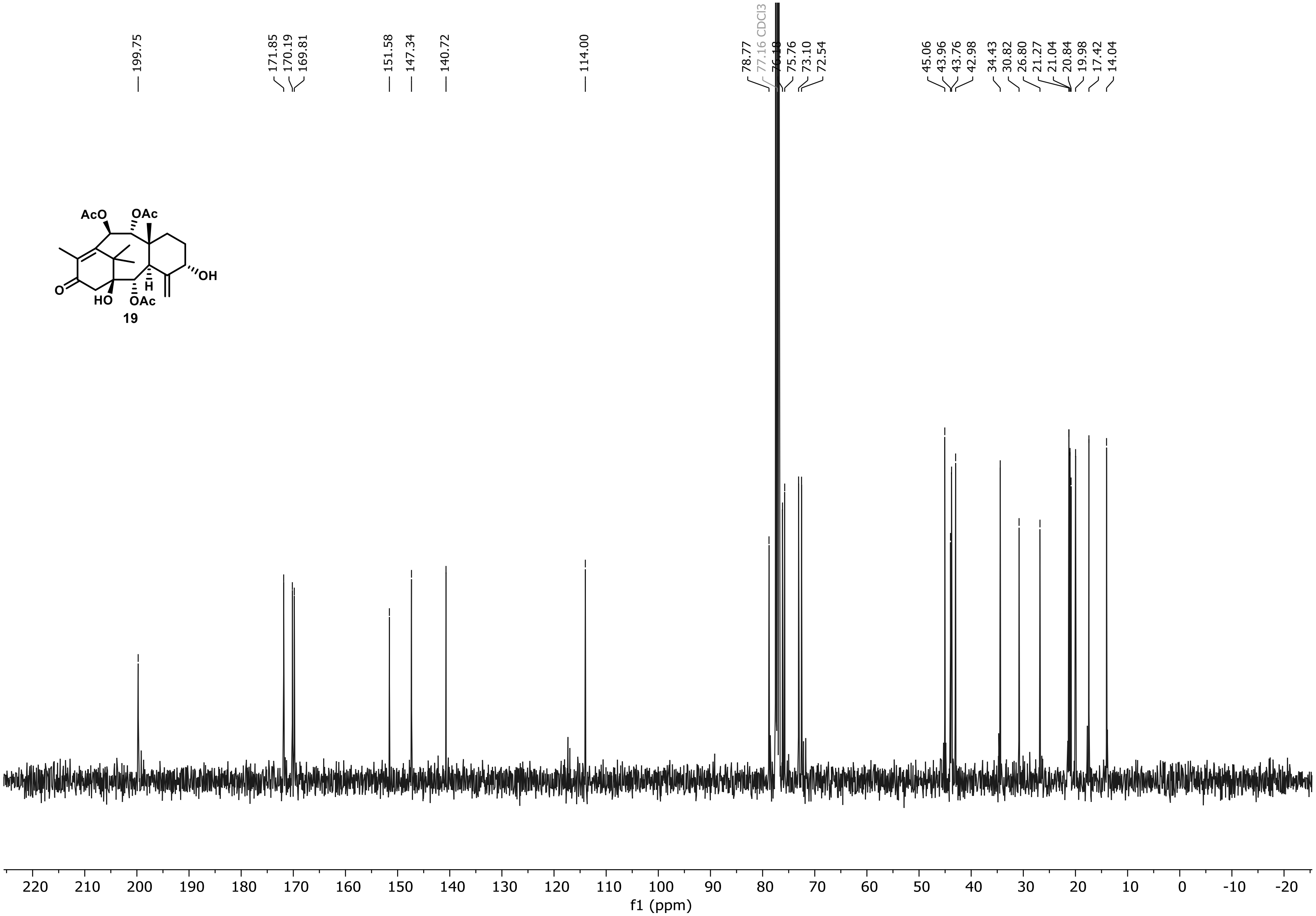

^1H NMR (400 MHz, $CDCl_3$)

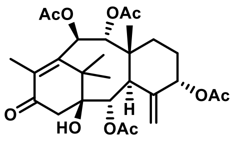

S8

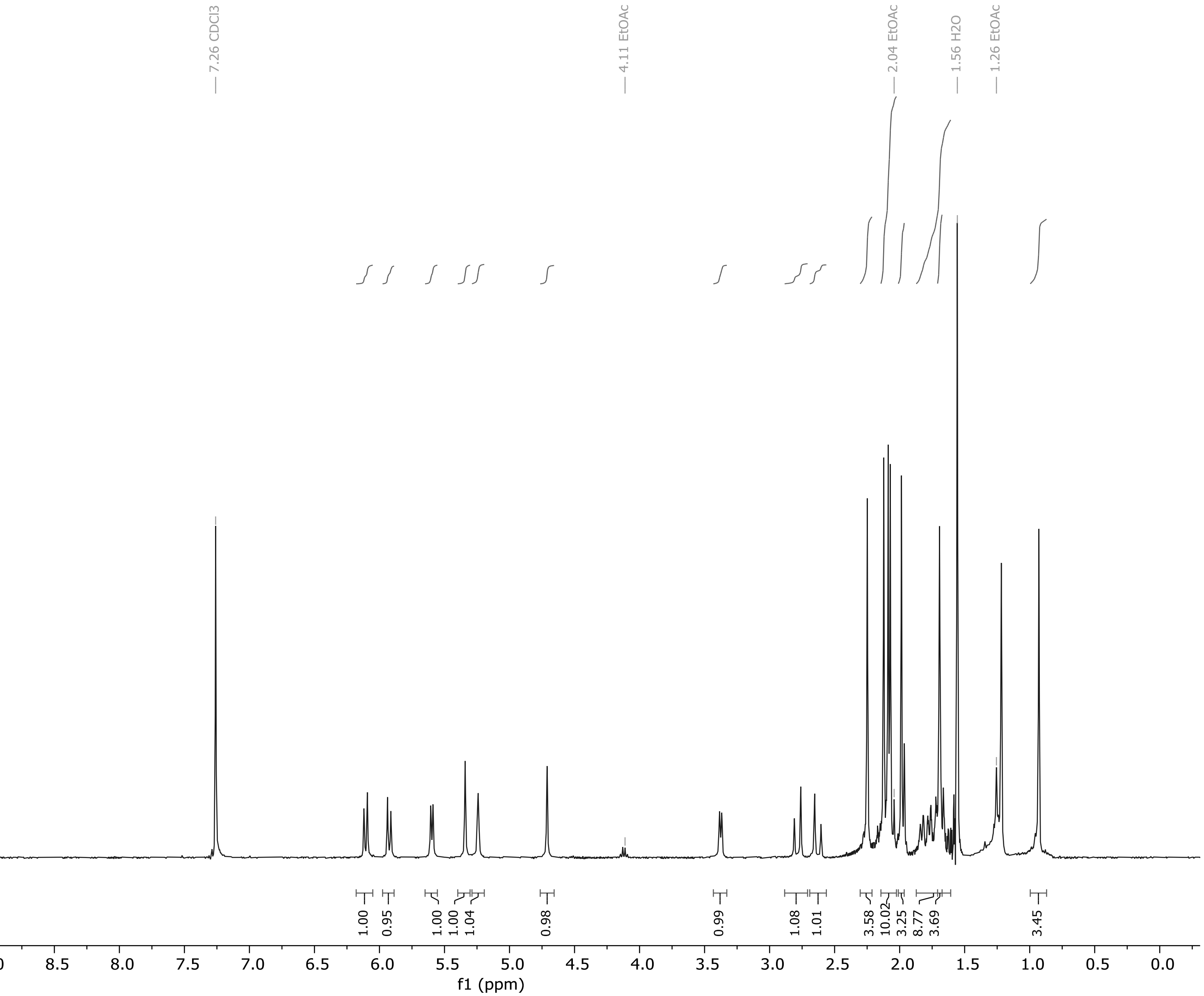

^{13}C NMR (101 MHz, $CDCl_3$)

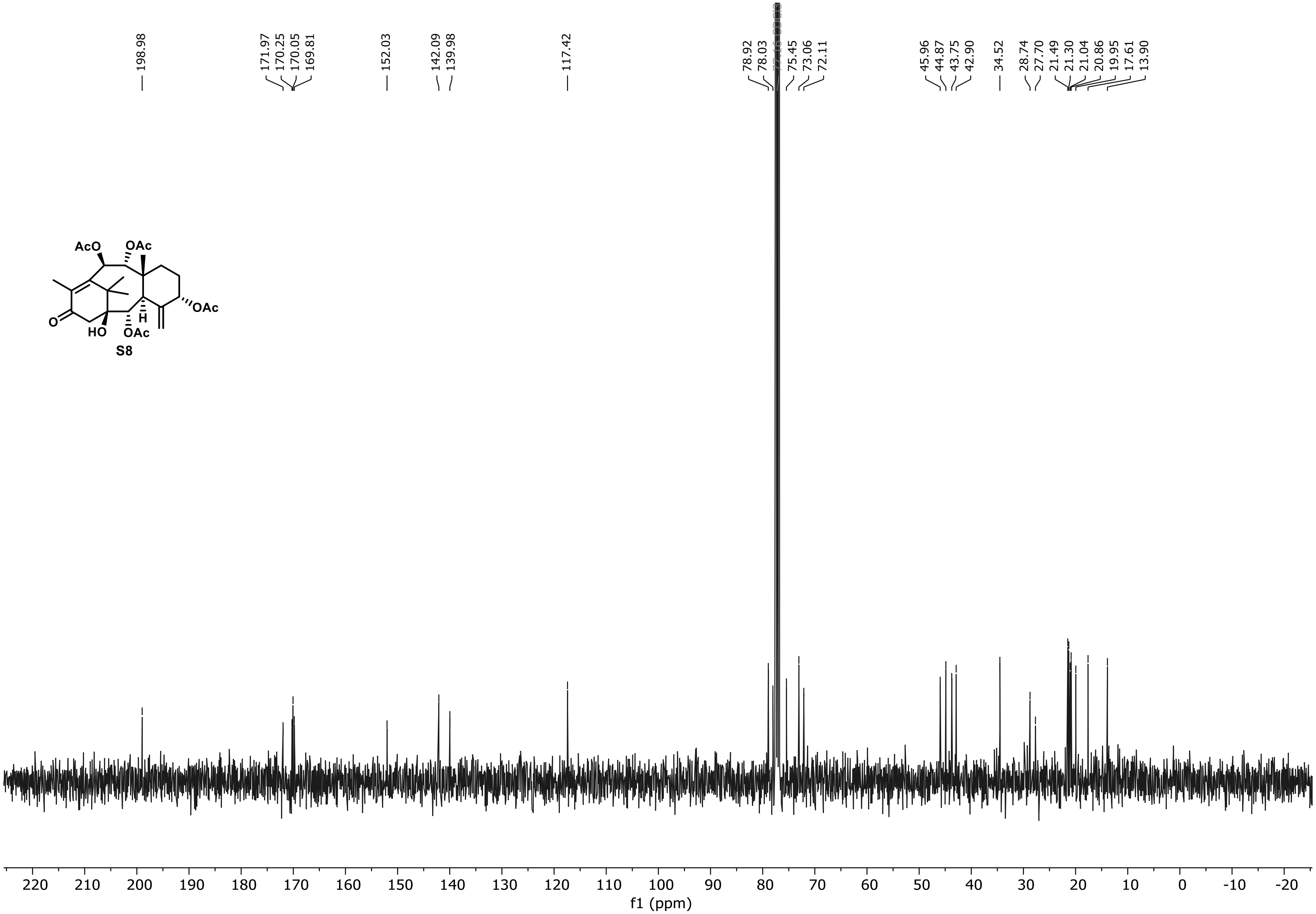

S8

^{1}H NMR (400 MHz, CDCl$_3$)

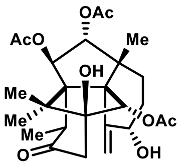

20

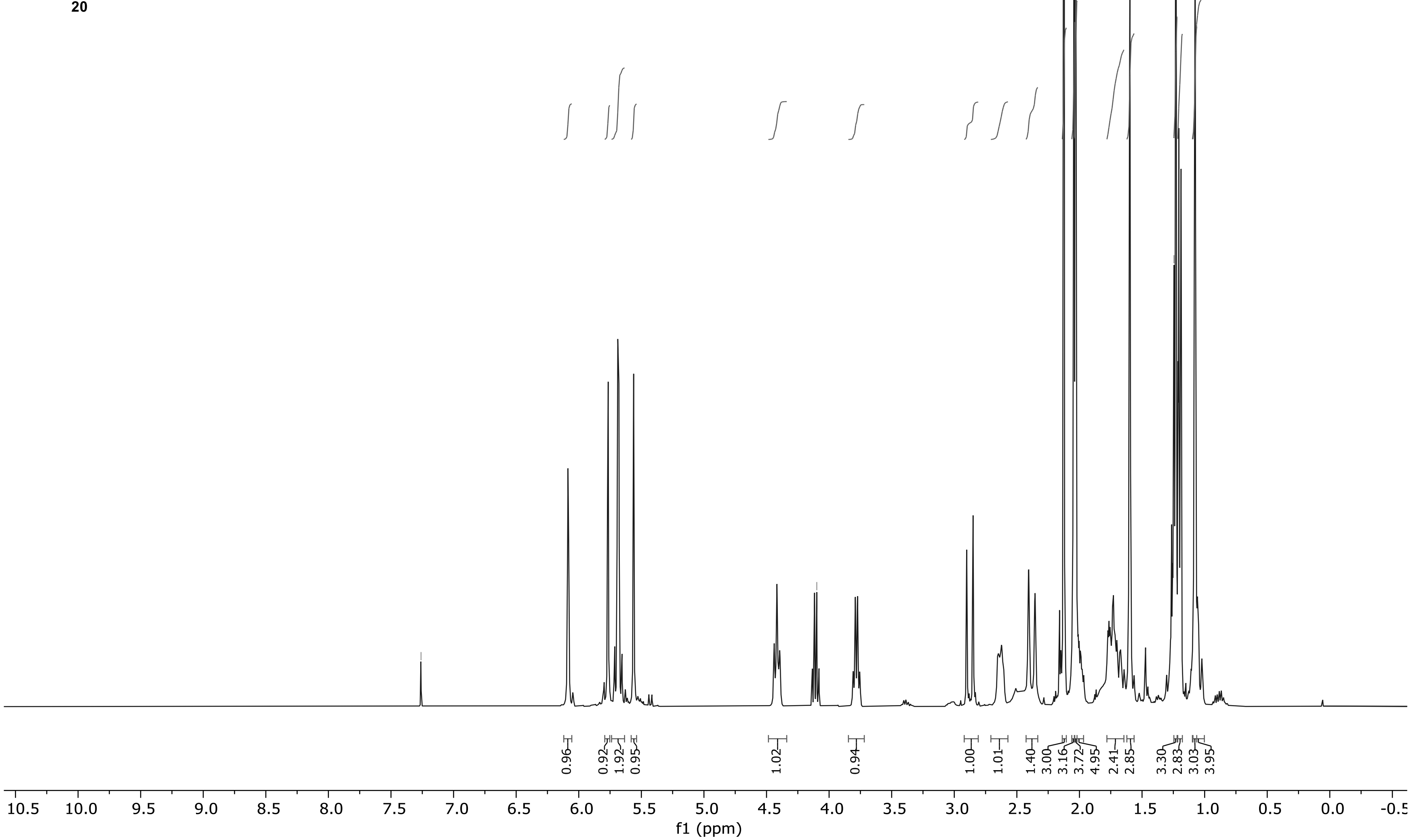

^{13}C NMR (101 MHz, $CDCl_3$)

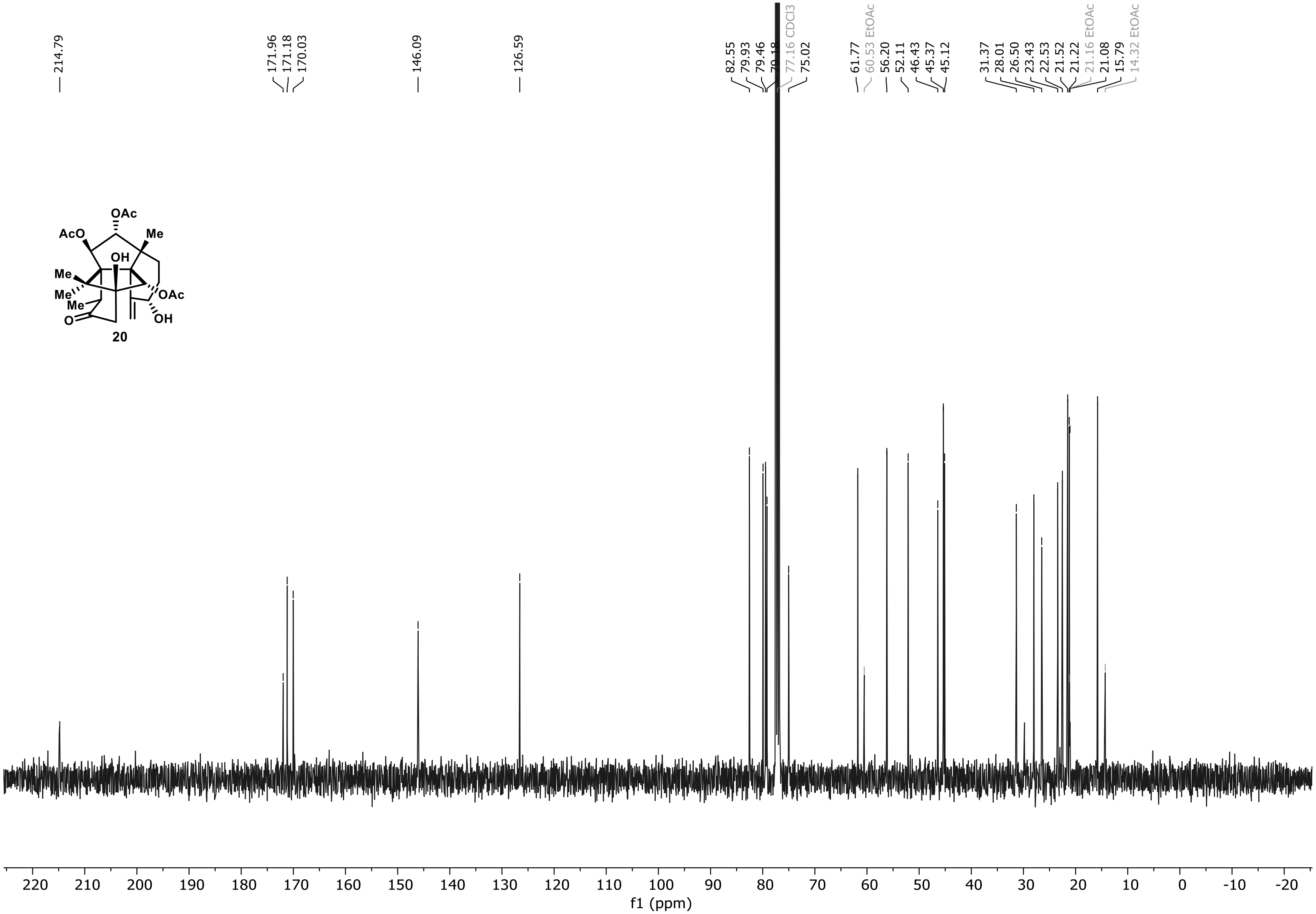

^{1}H NMR (400 MHz, $CDCl_3$)

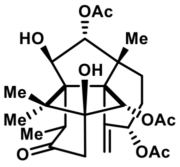

S6

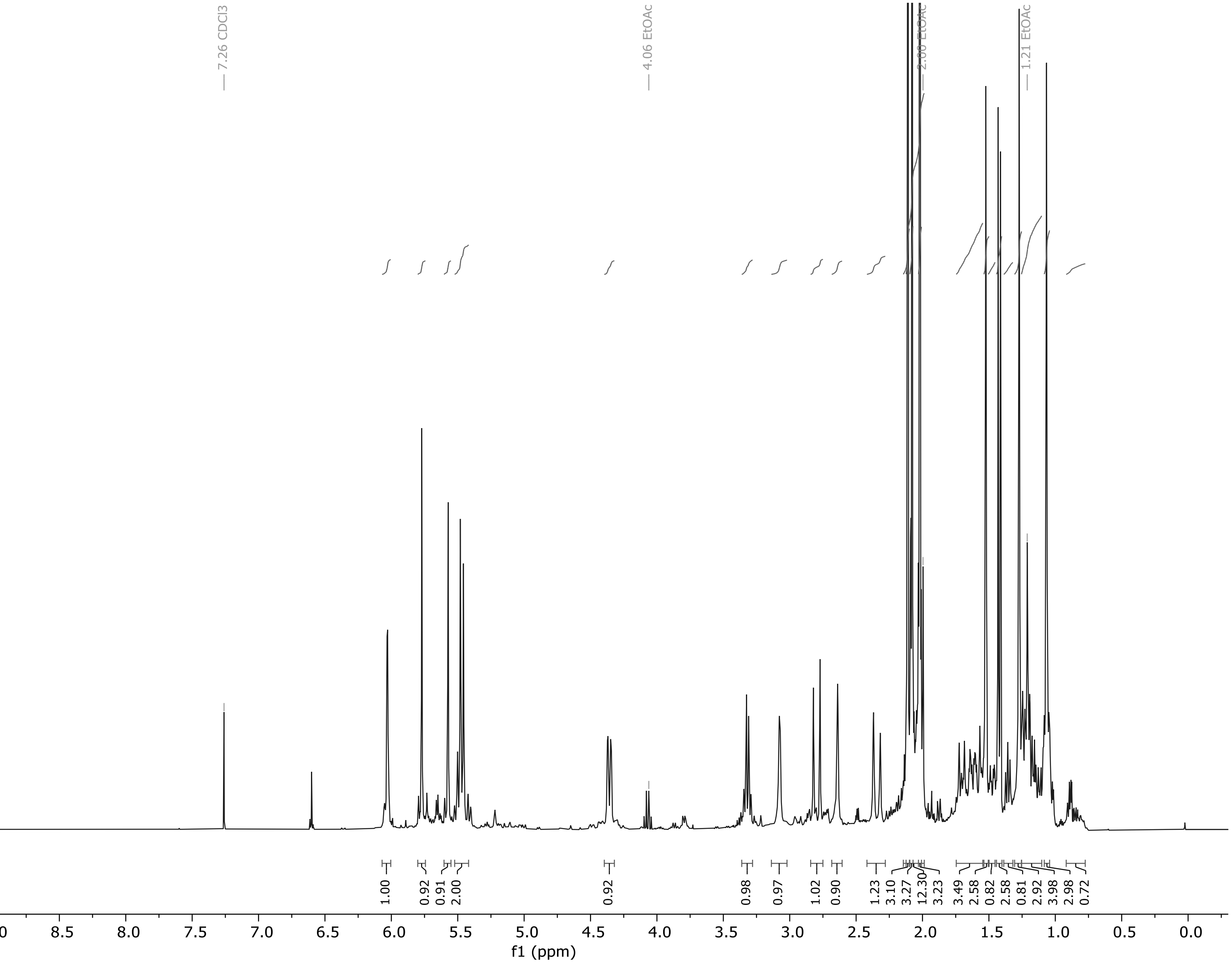

^{13}C NMR (101 MHz, $CDCl_3$)

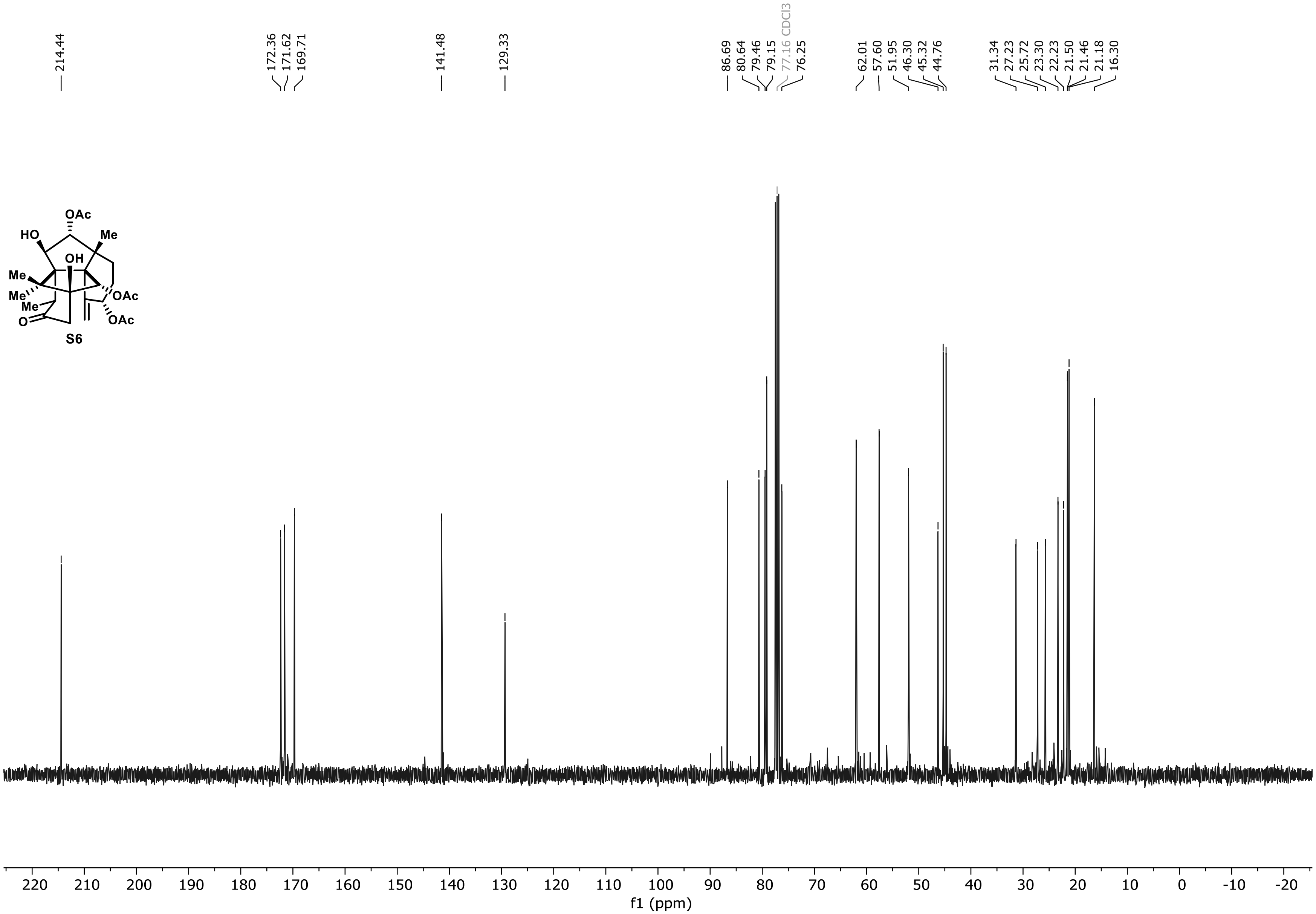

^1H NMR (400 MHz, $CDCl_3$)

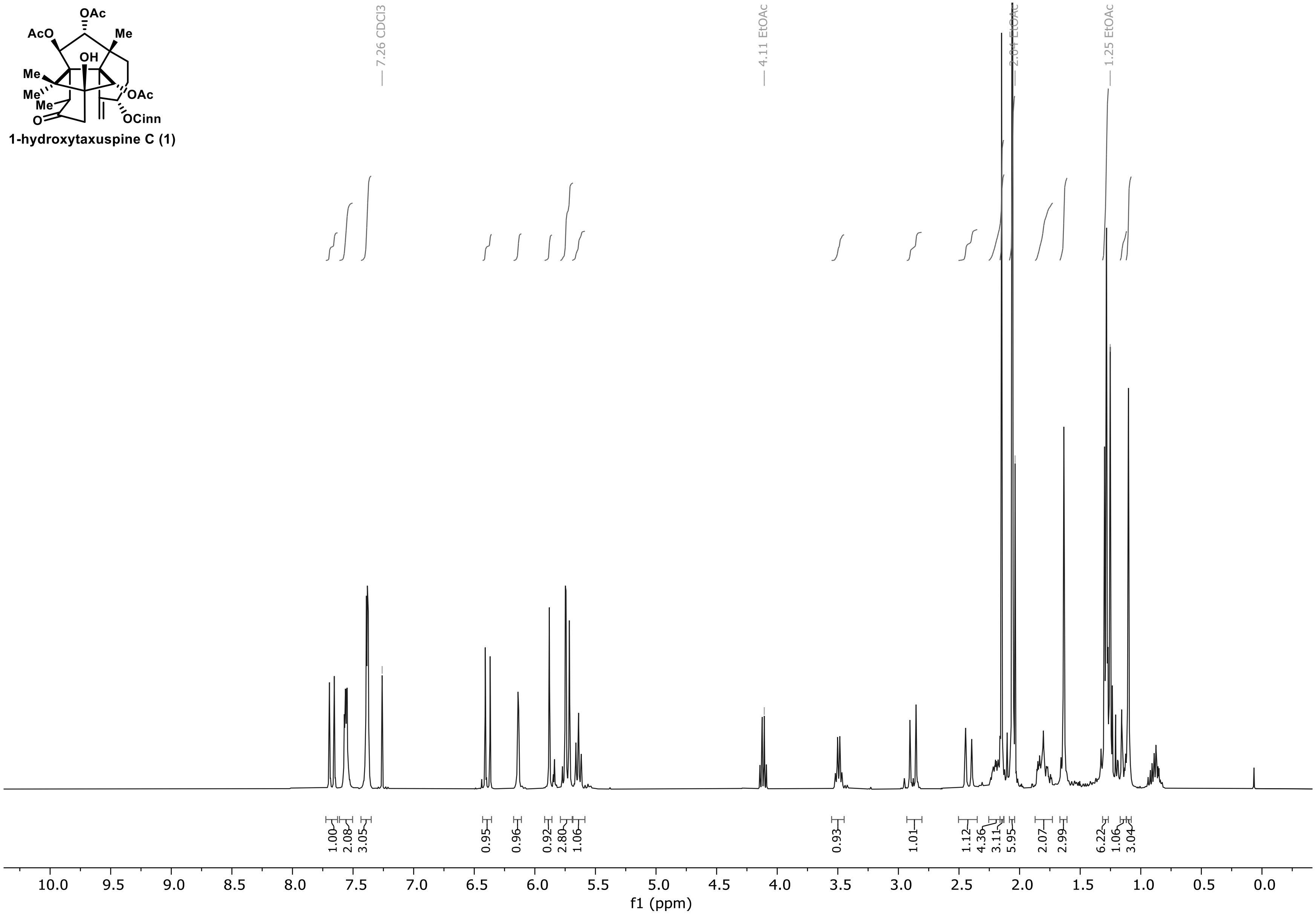

1-hydroxytaxuspine C (1)

^{13}C NMR (101 MHz, $CDCl_3$)

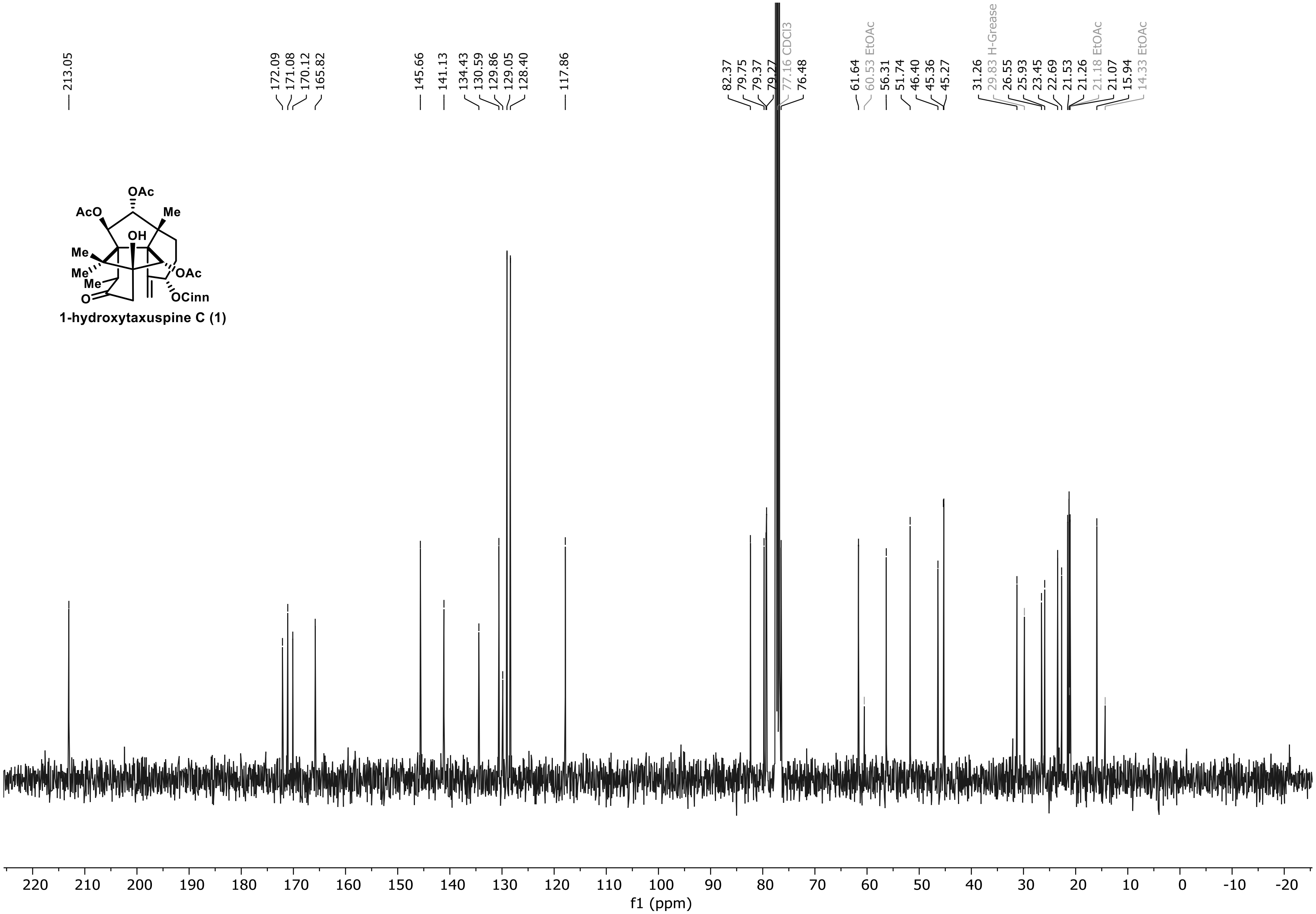

^{1}H-^{1}H COSY ($CDCl_3$)

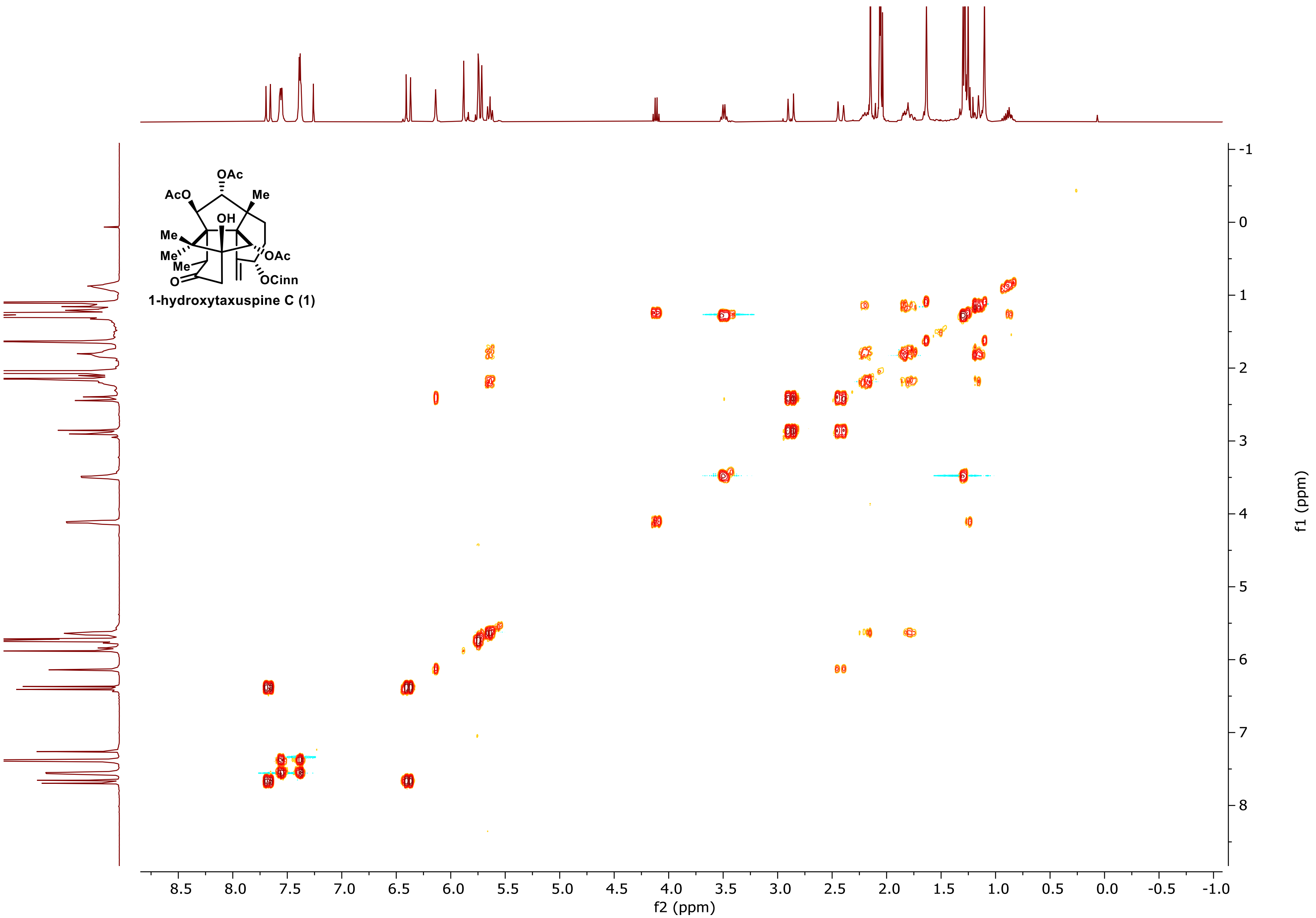

^{1}H-^{13}C HSQC ($CDCl_3$)

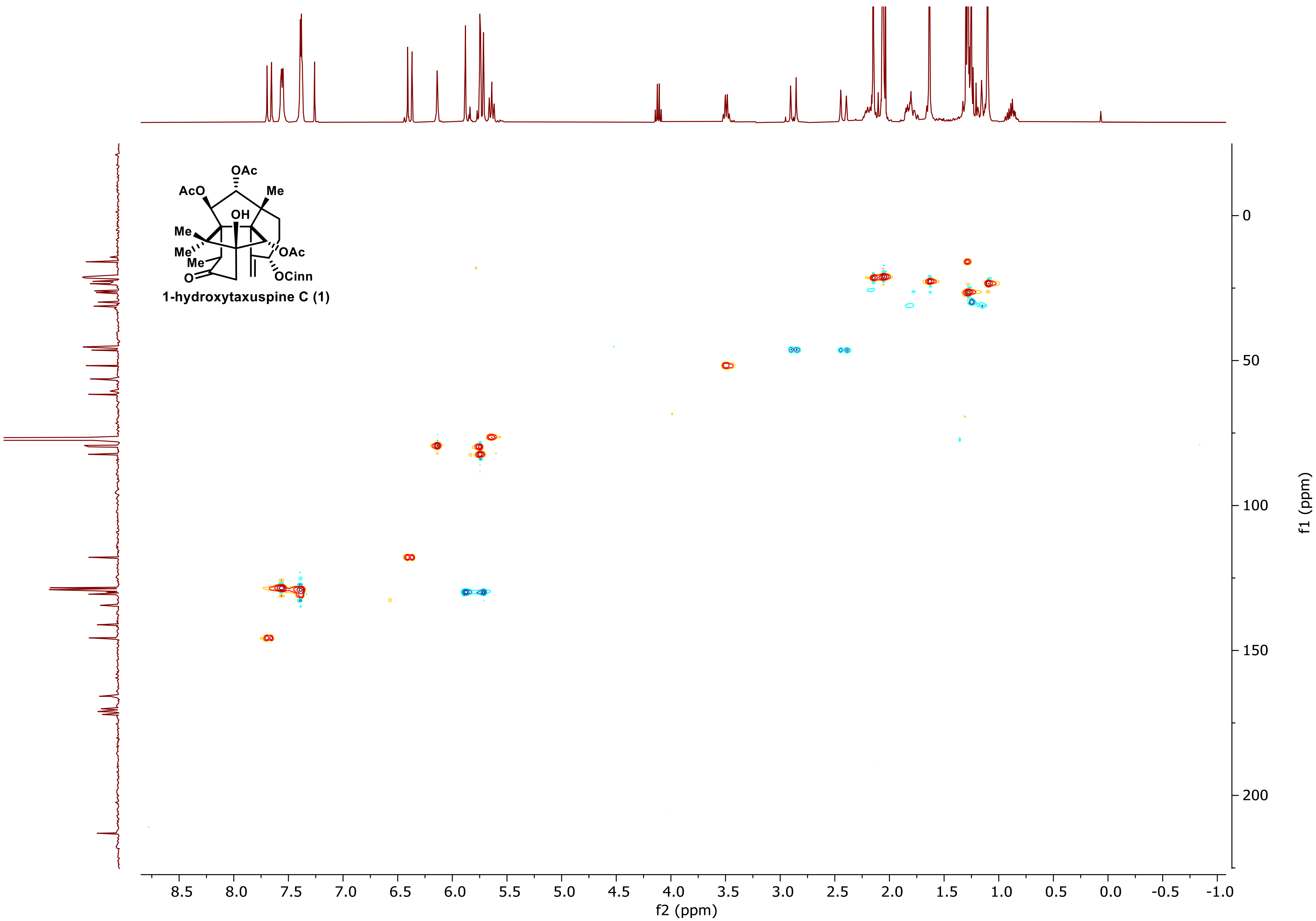

^{1}H-^{13}C HMBC ($CDCl_3$)

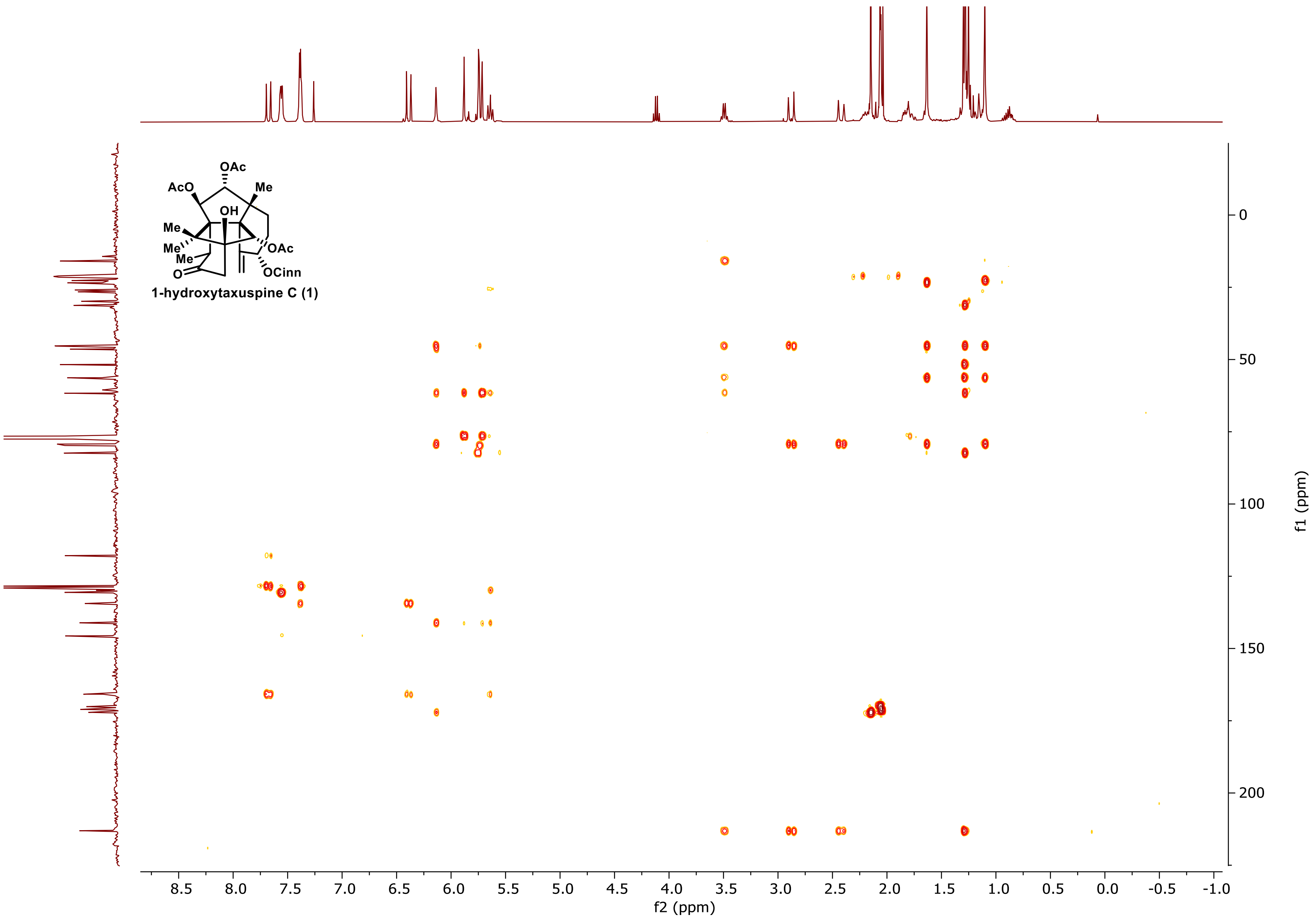

^1H-^1H NOESY ($CDCl_3$)

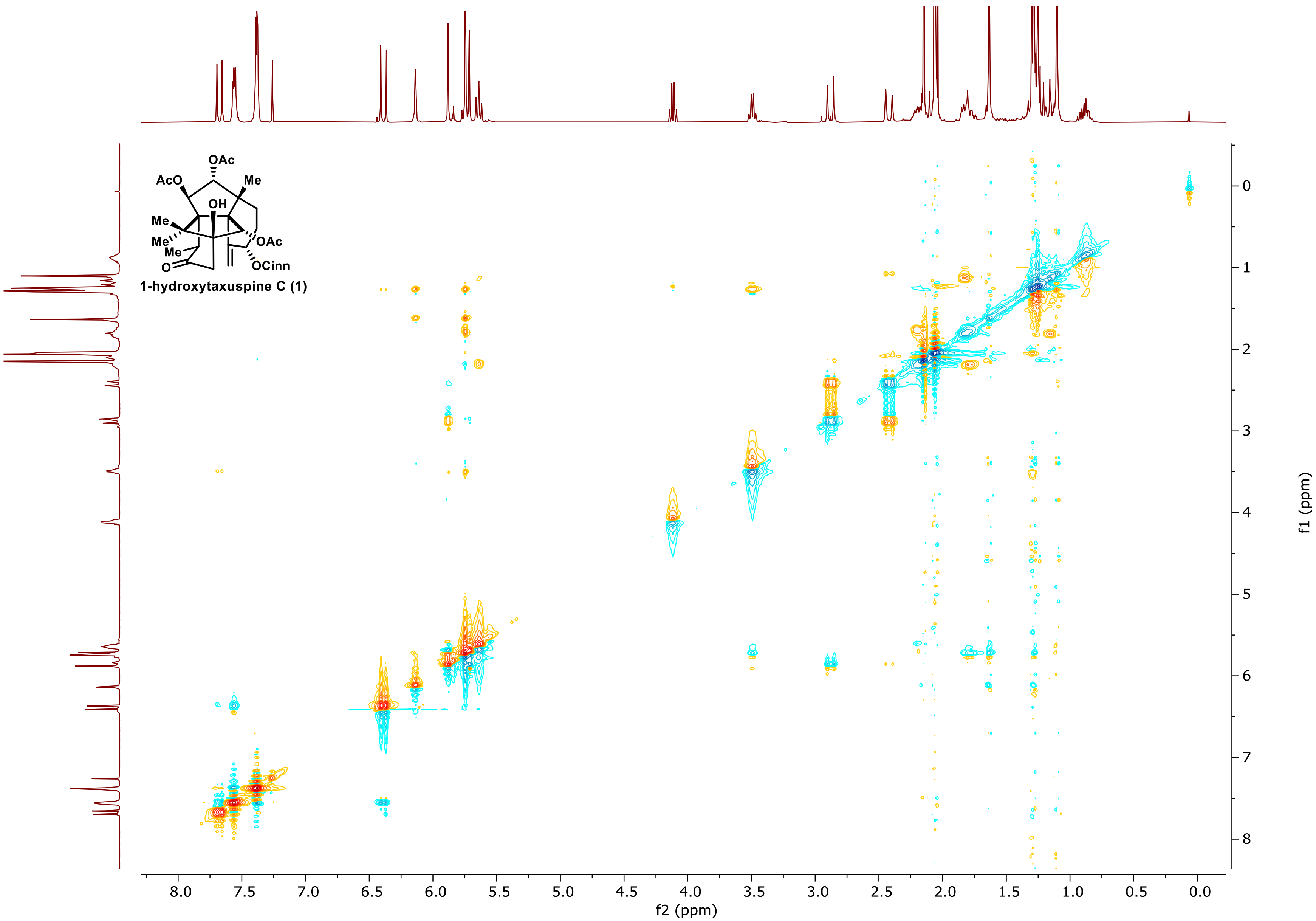

Supplement: Supplementary file 1 — Supporting Information [file ANIE-64-e202506245-s001.pdf]
